# Supplementary material for: Rapid and Focused Maturation of a VRC01-Class HIV Broadly Neutralizing Antibody Lineage Involves Both Binding and Accommodation of the N276-Glycan
Source: Immunity. 2019 Jul 16;51(1):141–154.e6. doi: 10.1016/j.immuni.2019.06.004 (PMC6642152; doi:10.1016/j.immuni.2019.06.004)
Supplement: Document S1. Figures S1–S6, Tables S1–S5, and Supplemental Experimental Procedures [file mmc1.pdf]

Immunity, Volume *51*

Supplemental Information

Rapid and Focused Maturation of a VRC01-Class HIV Broadly Neutralizing Antibody Lineage Involves Both Binding and Accommodation of the N276-Glycan

Jeffrey Umotoy, Bernard S. Bagaya, Collin Joyce, Torben Schiffner, Sergey Menis, Karen L. Saye-Francisco, Trevor Biddle, Sanjay Mohan, Thomas Vollbrecht, Oleksander Kalyuzhniy, Sharon Madzorera, Dale Kitchin, Bronwen Lambson, Molati Nonyane, William Kilembe, The IAVI Protocol C Investigators, The IAVI African HIV Research Network, Pascal Poignard, William R. Schief, Dennis R. Burton, Ben Murrell, Penny L. Moore, Bryan Briney, Devin Sok, and Elise Landais

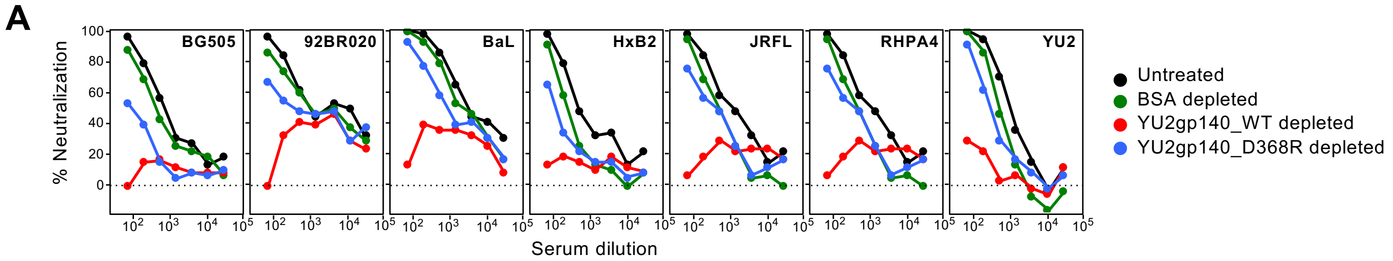

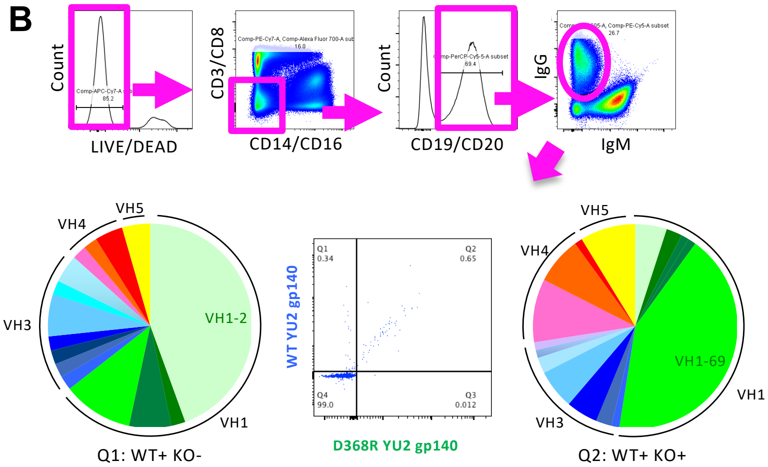

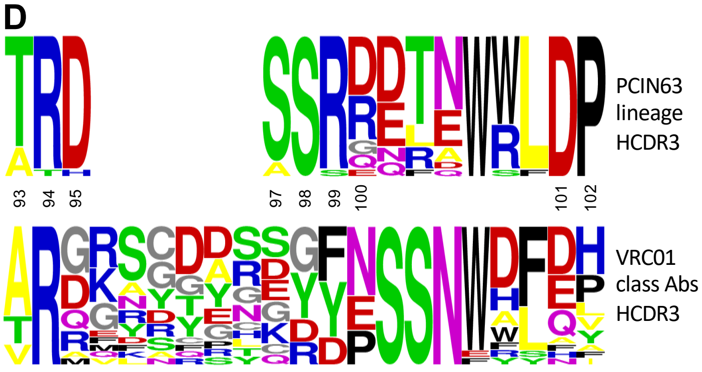

C

		Heavy Chain					Light Chain Chain				
		Putative Germline Genes: IGHV1-2*02 / IGHJ5*02					IGKV1-5*03 / IGKJ1*01				
mpi	mAb name	CDRH3 sequence (15aa)	V_H+J_H % SHM (nt)	V_H+J_H % SHM (aa)	UCA-H % SHM (nt)	UCA-H % SHM (aa)	CDRL3 sequence (5aa)	$V\kappa+J\kappa$ % SHM (nt)	$V\kappa+J\kappa$ % SHM (aa)	UCA-K % SHM (nt)	UCA-K % SHM (aa)
66	66Aa						QVVQW	12.04	15.63	14.56	17.76
66	66B	TRDSSRRDTNWWLDP	9.59	18.42	11.38	20.33	QVLET	12.71	17.71	14.24	18.69
71	71Aa						QVYES	10.70	19.79	12.30	20.56
71	71B	TTHSSSRDFQWSLDP	9.88	16.67	11.92	20.33	QVYES	11.04	16.67	12.94	17.76
71	71D2b	TRDSSRRDTNWWLDP	10.47	21.05	12.20	22.76	QVLET	12.71	19.79	14.24	20.56
71	71C	TRDSSRRDTNWWLDP	10.76	20.18	12.47	21.95	QVLET	12.37	18.75	13.92	19.63
71	71E1	TRDSSRRDTNWWLDP	10.76	20.18	12.47	21.95					
71	71F	TRDSSRQQRDWWLDP	12.50	21.05	13.01	21.14	QVYES	11.04	18.75	12.62	19.63
71	71G	TRDASRDDRAWRLDP	13.08	21.05	15.72	23.58	QVVEW	12.37	15.63	14.56	16.82
71	71H	ARDSSREETNWWLDP	13.08	21.93	14.36	23.58	QVLET	13.04	20.83	14.24	21.50
71	71I1a	TRDASRDDRAWRLDP	13.66	21.05	16.26	23.58	QVFEW	13.04	17.71	14.56	18.69
71	71J2a	TRDASRDDRAWRLDP	10.76	20.18	12.47	21.95	QVLET	12.71	18.75	14.24	19.63
71	71K	TRDSSRDETNWWLDP	14.24	23.68	15.45	25.20	QVLET	13.04	18.75	14.56	19.63
71	71L	TRDSSRDETNWWLDP	14.24	24.56	15.45	26.02	QVLET	13.04	18.75	14.56	19.63
71	71M1a	ARDSSRDETNWWLDP	14.53	21.05	15.72	22.76	QVLET	12.37	18.75	13.92	19.63
71	71N1a	TRDSSRGNTEWRLDP	14.53	21.05	15.72	22.76	QVYQT	12.71	18.75	14.89	20.56
71	71O	TRDSSRDNLEWRLDP	15.99	23.68	17.07	23.58	QVFEW	12.71	15.63	14.56	16.82
71	71P	TRDSSRGDTEWRLDP	15.99	23.68	17.89	24.39	QVFEW	13.04	16.67	14.89	17.76
77	77A	TTHSSRRDFQWSLDP	10.47	17.54	12.20	20.33					
77	77B1b	TRDSSRDETNWWLDP	11.34	23.68	13.82	25.20	QVLET	12.71	19.79	14.24	20.56
77	77C	TRDSSRRDLEWRLDP	11.92	21.05	12.74	21.14	QVLET	13.71	19.79	15.53	20.56
77	77D	ARDSSRDETNWWLDP	13.08	20.18	14.36	21.95	QAYES	12.37	19.79	14.24	20.56
77	77E1	TRDSSRDETNWWLDP	13.95	23.68	15.18	25.20					
77	77F1	TRDSSRDETNWWLDP	14.53	23.68	15.72	25.20					

Figure S1: Isolation of CD4bs-specific antibodies in participant PC063 – Related to Figure 1.
(A) Neutralization of HIV-1 pseudoviruses by titrated amount of PC063 month-66 (M66) untreated plasma or after adsorption on BSA- or rgp140-coated beads. This data is from a single experiment.
(B) Fluorescence Activated Cell Sorting (FACS) cell plots highlighting cell selection strategy for CD4bs specific B-cell isolation from PC063. Pie charts detail the distribution of VH-gene IMGT assignment of IgG amplicons generated from rgp140 WT+D368R- and rgp140 WT+D368R+ single sorted cells in (Quadrant #1, Q1, left) and (Quadrant #2, Q2, right).
(C) Statistics for the PCIN63 heavy chain and light chain sequences obtained from the VH1-2+ single B-cell isolated in (B). The percent of somatic hyper mutations at the nucleotide (nt) and amino-acid (aa) levels compared to the IMGT database germline gene sequences and NGS-identified unmutated common ancestor (UCA) (see Figure 3A), were calculated for V and J heavy chain (VH+JH) and light chain (VK+JK) segments. The CDR3 definitions are based on IMGT database guidelines.
(D) Logogram of the HCDR3 amino acid sequences for PCIN63 and VRC01-class Abs.

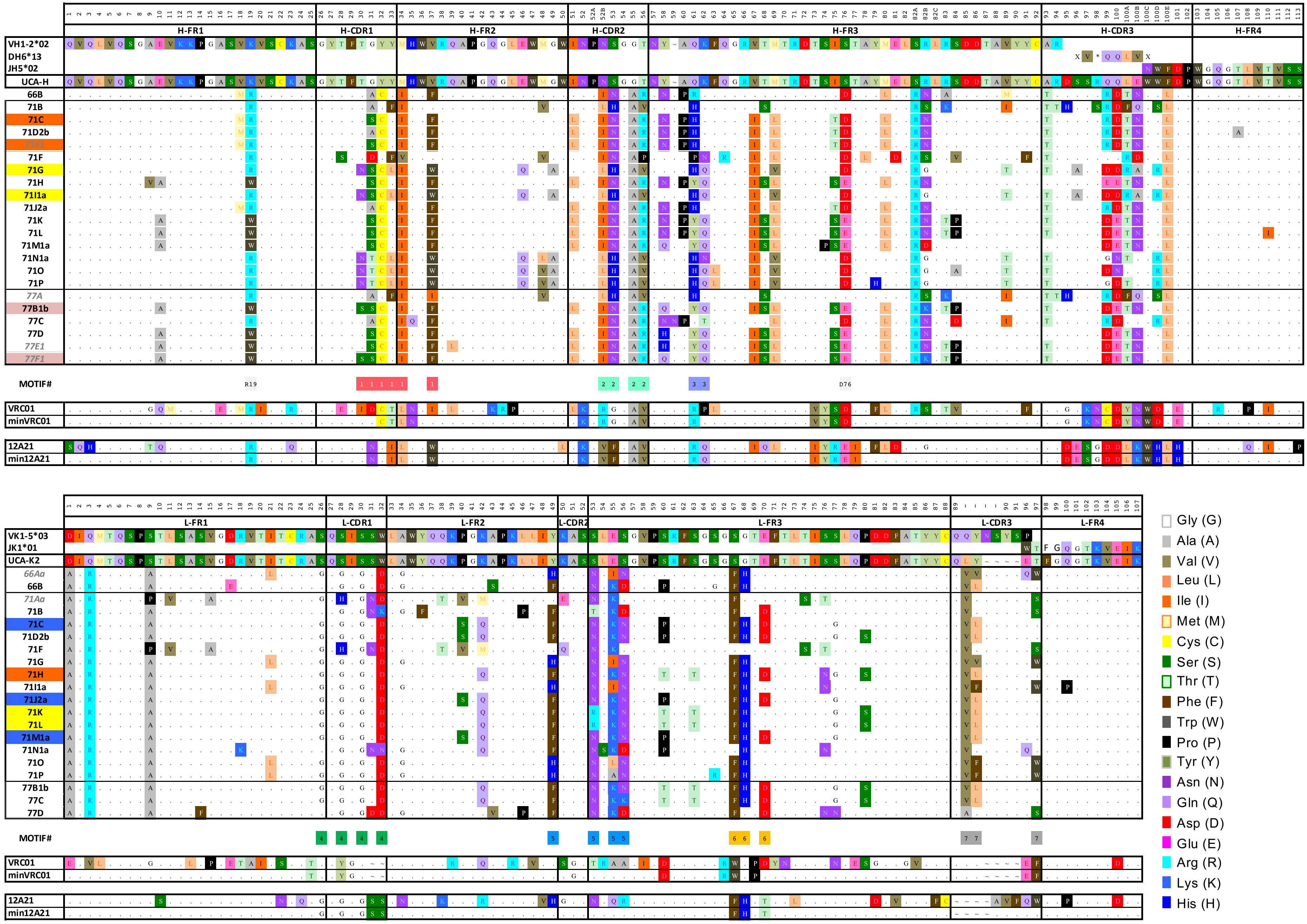

Figure S2. PCIN63 mAbs heavy chain and light chain amino acid alignments with putative germline genes – Related to Figure 1.
PCIN63 mAbs HC (top) and LC (bottom) amino-acid sequences are aligned to the IMGT database V_H1-2*02, D_H6-13, J_H5*02, Vκ1-5*03 and Jκ1*01 sequences. Identical residues are marked as a dot and somatically mutated residues are specified using one-letter coding. Deletions are marked by a ~ symbol. CDR and FRW regions are indicated. Sequences from different mAbs sharing perfect amino-acid identity are highlighted in colors. Unpaired sequences are showed in grey and italic

Clade	Time point / Ab name	PCIN 66B	PCIN 71B	PCIN 71C	PCIN 71D2b	PCIN 71F	PCIN 71G	PCIN 71H	PCIN 71I1a	PCIN 71J2a	PCIN 71K	PCIN 71L	PCIN 71M1a	PCIN 71N1a	PCIN 71O	PCIN 71P	PCIN 77B1b	PCIN 77C	PCIN 77D	PC63 M33 Serum	VRC01 min	VRC01 MAT	12A21 min	12A21 MAT
	SHM (V_H+J_H)	9.59	9.88	10.76	10.76	12.50	13.08	13.08	13.66	13.37	14.24	14.24	14.53	14.53	15.99	15.99	11.34	11.92	13.08		7.22	21.31	3.44	12.37
	SHM (V_K+J_K)	12.71	11.04	12.37	12.71	11.04	12.37	13.04	13.04	12.71	13.04	13.04	12.37	12.37	12.71	13.04	13.04	13.71	12.37		6.48	29.32	8.33	21.91
Ctrl	MLV	>100	>100	>100	>100	>100	>100	>100	>100	>100	>100	>100	>100	>100	>100	>100	>100	>100	>100	6	>100	>100	>100	>100
	VSV	>100	>100	>100	>100	>100	>100	>100	>100	>100	>100	>100	>100	>100	>100	>100	>100	>100	>100		>100	>100	>100	>100
	92RW020	0.79	>100	0.67	0.59	>100	0.86	0.26	0.39	1.1	0.20	0.096	0.20	>100	0.58	2.2	9.5	0.94	2.4	491	0.11	0.50	0.09	0.06
	94UG103	>100	>100	>100	>100	>100	4.3	10	3.1	>100	7.1	5.7	2.4	>100	7.0	18	>100	>100	>100	1	0.20	0.35	>100	0.46
A	BG505	1.3	>100	5.2	>100	>100	1.4	1.1	0.49	0.76	0.72	>100	139	>100	0.75	1.5	15	1.1	29	564	0.12	0.09	0.12	0.09
	KNH1144	1.2	>100	0.98	1.5	>100	5.1	0.45	1.3	1.1	0.32	0.42	0.66	>100	1.9	23	37	4.3	48	248	1.56	0.37	0.59	0.54
	KNH1207	0.38	>100	0.23	0.47	>100	1.2	0.33	0.36	0.25	0.29	0.29	0.62	>100	0.57	3.0	68	1.3	>100	.nt.	63	0.88	0.68	0.32
AE	92TH021	0.36	>100	0.12	9.1	>100	0.60	0.11	0.13	0.23	0.12	0.33	6.8	>100	0.30	0.36	0.94	0.24	>100	456	0.28	0.24	0.09	0.06
	89.6	>100	>100	3.0	1.8	>100	0.24	>100	0.22	2.3	14	>100	>100	>100	1.4	0.60	>100	>100	>100	1	0.78	0.63	>100	1.96
	6535	0.62	>100	0.18	0.22	>100	1.9	2.0	0.19	0.16	0.42	0.16	0.13	>100	0.74	0.54	>100	62	>100	263	0.29	0.32	>100	9.15
	92BR020	0.26	>100	0.15	0.25	>100	0.64	0.20	0.23	0.32	0.14	0.13	0.18	>100	0.59	0.58	10	0.69	>100	834	0.81	0.04	0.11	0.08
	ADA	45	>100	2.1	5.1	>100	2.3	2.6	1.5	3.2	1.3	0.54	0.88	>100	5.6	16	50	>100	>100	192	0.32	0.48	>100	0.82
	BaL	0.11	0.041	0.025	0.044	0.71	0.093	44	0.017	0.068	1.9	1.0	21	>100	0.25	0.085	0.0074	>100	2.8	1790	0.01	0.02	>100	0.12
	CAAN	2.2	>100	0.93	0.49	>100	0.58	3.2	2.4	1.6	1.9	1.2	10	>100	9.4	7.9	8.4	10	>100	1	0.25	0.42	8.86	0.46
	DH12	46	>100	2.6	4.3	>100	3.8	32	2.6	3.4	21	8.3	5.6	>100	9.7	10	38	4.1	>100	823	0.12	0.40	>100	48
	HxB2	0.75	>100	0.067	0.24	>100	0.098	0.72	0.043	0.12	0.26	0.17	0.10	>100	0.083	0.13	0.48	>100	>100	926	0.01	0.05	>100	>100
	JRCSF	4.6	>100	0.60	0.91	>100	1.5	1.3	0.43	1.6	0.56	0.70	0.92	>100	1.4	2.7	1.6	3.3	>100	182	0.12	0.14	>100	0.43
	JRFL	0.064	>100	0.022	0.055	>100	0.14	0.082	0.053	0.052	0.053	0.037	0.070	>100	0.11	0.42	0.35	0.13	2.0	2275	71.60	0.61	0.05	0.04
B	MN	0.15	>100	0.12	0.088	>100	0.075	>100	0.072	0.13	>100	>100	>100	>100	0.29	0.16	0.49	>100	>100	496	0.03	0.09	>100	20
	PVO4	1.1	>100	0.28	0.32	4.48	2.6	0.35	0.47	0.30	0.23	0.19	0.68	>100	0.89	2.4	7.2	2.4	28	402	0.19	0.35	0.29	0.41
	QHO692	6.0	>100	2.3	2.1	>100	12	1.4	1.8	1.3	1.4	1.0	1.9	>100	6.0	35	16	8.0	>100	198	3.23	1.84	7.20	4.83
	REJO	0.13	>100	0.039	0.058	>100	0.13	0.050	0.034	0.065	0.15	0.094	0.086	>100	0.13	0.15	0.096	0.42	>100	273	0.04	0.09	>100	0.79
	RHPA4	0.20	>100	0.048	0.12	>100	0.15	0.030	0.050	0.074	0.029	0.022	0.052	>100	0.089	0.23	1.8	0.16	3.4	705	21	0.04	1.37	0.04
	SC422	0.50	>100	0.12	0.36	>100	0.52	0.13	0.081	0.15	0.19	0.11	0.39	>100	0.24	0.96	1.1	>100	>100	222	0.30	0.09	>100	2.70
	SF162	>100	>100	2.4	22	>100	0.39	>100	0.19	0.99	44	12	0.89	>100	0.45	0.45	6.0	>100	>100	834	0.04	0.14	>100	0.67
	SS1196	>100	>100	8.9	35	>100	1.1	>100	0.37	9.0	19	13	3.6	>100	1.3	2.7	>100	>100	>100	378	0.09	0.22	>100	>100
	TRJO	>100	>100	272	>100	>100	0.59	0.24	0.11	62	0.080	0.080	0.12	>100	0.19	0.16	>100	>100	>100	246	0.12	0.08	>100	1.25
	TRO11	0.37	>100	0.24	0.28	>100	0.47	0.080	0.18	0.18	0.049	0.035	0.068	>100	0.31	0.79	27	0.46	99	439	2.84	0.52	0.26	0.22
	WITO	0.82	>100	0.13	0.12	>100	0.24	0.092	0.086	0.085	0.051	0.048	0.062	>100	0.078	0.078	>100	0.24	>100	496	1.07	0.17	2.89	0.22
	YU2	0.13	>100	0.058	0.10	>100	0.65	0.085	0.18	0.087	0.067	0.056	0.11	>100	0.60	0.92	7.4	0.17	15	1170	0.05	0.09	0.15	0.05
	93IN905	0.73	>100	0.17	0.36	>100	0.34	0.063	0.090	0.21	0.035	0.014	0.056	>100	0.17	0.58	40	0.33	0.59	701	0.06	0.13	2.26	0.06
	CAP210	>100	>100	>100	>100	>100	>100	>100	45	>100	>100	>100	>100	>100	>100	>100	>100	>100	>100	1	>100	>100	>100	>100
	CAP45	>100	>100	>100	>100	>100	>100	>100	0.053	>100	>100	>100	>100	>100	0.13	0.053	>100	>100	>100	1	>100	0.27	>100	0.06
	DU156	>100	>100	7.9	21	>100	0.12	0.51	0.034	6.6	0.11	0.067	1.7	>100	0.070	0.15	>100	0.014	>100	654	0.05	0.06	6.78	0.03
	DU172	4.8	>100	0.37	0.75	>100	0.75	0.25	0.29	0.60	0.27	0.16	0.21	>100	0.38	0.76	67	1.5	10	589	>100	>100	0.56	0.29
C	DU422	>100	>100	>100	>100	>100	>100	>100	>100	>100	>100	>100	>100	>100	>100	>100	>100	>100	>100	1	>100	>100	>100	3.37
	IAVIC22	>100	>100	20	52	>100	0.066	2.6	2.6	40	2.4	2.0	2.6	>100	7.9	21	>100	>100	>100	177	0.28	0.54	>100	67
	ZM53	1.1	>100	0.45	0.79	>100	1.1	0.39	0.35	0.36	0.26	0.52	0.36	>100	0.69	3.6	>100	0.77	24	.nt.	0.54	0.79	0.63	0.25
	ZM109	2.2	>100	0.98	1.0	>100	1.3	0.49	0.62	1.4	0.40	0.44	1.0	>100	7.4	9.9	>100	>100	>100	.nt.	28	0.11	>100	5.05
	ZM135	3.2	>100	1.3	1.9	>100	7.5	4.5	1.5	1.3	1.3	1.6	3.1	>100	7.4	14	>100	48	>100	1	63	1.17	>100	>100
	ZM214	0.17	>100	0.12	0.12	0.12	0.013	0.084	0.14	0.16	0.080	0.012	0.015	>100	0.19	0.14	0.74	0.021	0.46	1	0.18	0.31	0.15	0.21
	ZM249	0.43	>100	0.16	0.43	>100	0.15	0.055	0.080	0.14	0.046	0.044	0.058	>100	0.11	0.58	5.9	>100	>100	330	0.08	0.09	22	0.10

Table S1. Neutralization IC50s of PCIN63 Abs on a medium cross-clade pseudovirus panel. – Related to Figure 1 and Table S2

IC50 is defined as the Ab concentration in µg/mL allowing 50% loss of infectivity.
PCIN63 Abs are organized from least to most mutated.
Data are representative of at least 2 experiments.

Breadth (viruses neutralized)	Potency mAb (GeoMean IC_{50})	Potency Plasma (GeoMean ID_{50})
0-10%	10-50 µg/mL	/
10-25%	1-10 µg/mL	50-100
25-50%	0.1-1 µg/mL	100-300
50-75%	0.01-0.1 µg/mL	300-900
75-100%	<0.01 µg/mL	>900

Ab name	Breadth (% viruses) IC_{50} <0.1µg/mL	IC_{50} <1µg/mL	IC_{50} <10µg/mL	IC_{50} <50µg/mL	GeoMean IC_{50} (µg/mL)	SHM (V_H+J_H)	SHM (V_K+J_K)
66B	3%	45%	70%	75%	0.8	9.6	12.7
71B	3%	3%	3%	3%	0.04	9.9	11.0
71C	15%	63%	85%	88%	0.50	10.8	12.4
71D2b	10%	55%	75%	83%	0.73	10.5	12.7
71F	0%	5%	8%	8%	0.72	12.5	11.0
71G	13%	58%	90%	93%	0.57	13.1	12.4
71H	23%	55%	75%	83%	0.48	13.1	13.0
71I1a	30%	75%	95%	98%	0.28	13.7	13.0
71J2a	15%	55%	85%	88%	0.56	10.8	12.7
71K	23%	63%	80%	90%	0.42	14.2	13.0
71L	30%	63%	80%	85%	0.27	14.2	13.0
71M1a	20%	58%	80%	85%	0.55	14.5	12.4
71N1a	0%	0%	0%	0%	/	14.5	12.7
71O	10%	65%	95%	95%	0.630	16.0	12.7
71P	8%	55%	78%	95%	1.1	16.0	13.0
77B1b	5%	18%	40%	58%	4.2	11.3	12.7
77C	5%	33%	53%	58%	0.92	11.9	13.7
77D	0%	5%	15%	30%	7.3	13.1	12.4
PC63M33	25%	65%	83%	88%	125		
min VRC01	25%	68%	78%	83%	0.37	6.5	7.2
VRC01	30%	88%	93%	93%	0.21	29.3	21.3
min 12A21	8%	33%	48%	50%	0.64	8.3	3.4
12A21	25%	65%	83%	88%	0.47	21.9	12.4

Table S2. Neutralization breadth and potency of PCIN63 Abs on a medium cross-clade pseudovirus panel. – Related to Figure 1 and Table S1

PCIN63 Abs are listed vertically by increasing frequency of somatic hypermutations.

Subtype	HIV.1 Isolate	Neutralization IC_{50} (mg/mL)									
		PCIN 66B	PCIN 71G	PCIN 71I	PCIN 71L	PCIN 77D	PC63 M33 serum	Min VRC01	Mat VRC01	Min 12A21	Mat 12A21
-	aMLV	>100	>100	>100	>100	>100	<20	>100	>100	>100	>100
	0260.v5.c1	1.5	1.7	1.7	0.58	0.95	NT	0.85	0.33	0.51	0.19
	0330.v4.c3	0.45	0.29	0.19	0.077	74	735	NT	NT	NT	NT
	191084 B7.19	0.86	0.21	0.17	0.19	>100	634	0.057	0.043	7.3	0.042
	191955 A11	>100	1.2	0.036	6.3	>100	2018	>100	>100	>100	>100
	9004SS A3 4	>100	>100	>100	>100	>100	NT	>100	>100	>100	>100
A	MS208.A1	0.73	0.28	0.24	0.19	>100	530	>100	0.024	>100	6.0
	Q168ENVa2	NT	NT	NT	NT	NT	NT	NT	NT	NT	NT
	Q23ENV17	0.36	0.18	0.15	0.080	0.17	736	0.074	0.042	0.12	0.026
	Q259ENVd2.17	>100	0.77	0.073	>100	>100	93	0.20	0.029	>100	0.10
	Q461ENVe2	6.8	7.7	7.1	2.2	>100	57	1.2	0.27	0.29	0.087
	Q769ENVd22	0.22	0.094	0.070	0.079	>100	1355	0.036	0.019	0.035	0.0089
	Q842ENVd12	>100	>100	>100	>100	>100	NT	>100	>100	>100	>100
	3301.v1.c24	0.68	0.49	0.39	0.33	>100	558	0.11	0.14	>100	0.049
AC	6041.v3.c23	0.063	0.066	0.057	0.022	0.097	<20	1.8	0.013	0.014	0.0069
	6540.v4.c1	>100	>100	>100	>100	>100	24	>100	>100	>100	>100
	6545.v4.c1	>100	>100	>100	>100	>100	2	>100	>100	>100	>100
ACD	0815.v3.c3	0.12	0.057	0.062	0.032	>100	1019	0.056	0.0100	0.033	0.012
	3103.v3.c10	>100	4.0	6.4	>100	>100	<20	4.1	1.3	>100	0.47
	620345.c01	>100	>100	>100	>100	>100	170	>100	>100	>100	>100
	BJOX009000.02.4	>100	4.2	2.9	11	>100	<20	2.9	0.64	>100	>100
	BJOX010000.06.2	>100	16	8.5	9.9	>100	57	6.5	3.9	>100	8.0
	BJOX015000.11.5	>100	1.4	0.99	0.44	>100	36	2.0	0.19	0.60	0.13
	BJOX025000.01.1	>100	0.58	0.49	26	0.16	311	7.3	2.7	0.27	0.13
	BJOX028000.10.3	0.079	0.11	0.076	0.052	>100	1813	11	0.086	0.029	0.0091
AE	C1080.c03	46	3.7	2.1	1.2	>100	466	>100	0.55	0.33	0.11
	C3347.c11	5.9	0.042	0.034	0.016	0.016	3162	0.039	0.050	0.0090	0.011
	C4118.c09	>100	>100	>100	>100	>100	477	>100	>100	>100	>100
	CNE5	4.0	0.29	0.18	0.35	>100	21	0.20	0.060	0.19	0.045
	CNE8	>100	0.44	0.36	0.36	>100	76	0.42	0.74	>100	0.28
	R1166.c01	4.0	0.46	0.33	0.23	2.3	337	0.62	0.39	0.17	0.097
	R2184.c04	4.0	0.17	0.13	0.24	>100	594	0.030	0.024	0.84	0.027
	R3265.c06	0.72	0.57	0.78	0.21	>100	138	0.73	0.17	0.057	0.044
	928.28	0.42	0.69	0.39	0.13	>100	1116	1.1	0.22	0.42	0.20
	T211.9	2.1	1.3	0.88	0.48	>100	NT	>100	0.90	>100	>100
	T235.47	>100	0.042	0.043	0.059	>100	432	>100	0.028	0.40	0.012
	T250.4	>100	>100	>100	>100	>100	25	>100	>100	>100	>100
AG	T251.18	9.7	7.3	8.8	1.9	>100	114	1.7	1.3	1.9	0.43
	T255.34	0.37	0.20	0.23	0.070	1.0	264	0.71	0.39	0.072	0.028
	T257.31	0.57	0.96	1.2	0.22	>100	203	0.71	0.31	0.078	0.028
	T263.8	0.35	0.14	0.17	0.070	1.6	NT	0.094	0.12	0.11	0.044
	T278.5	>100	>100	>100	>100	>100	<20	>100	>100	>100	>100
	1006.11.C3.1601	0.25	0.18	0.13	0.049	>100	1173	2.5	0.095	0.15	0.036
	1012.11.TC21.3257	0.28	0.87	0.49	0.65	>100	146	0.035	0.070	>100	0.035
	1054.07.TC4.1499	2.2	3.3	3.1	1.9	>100	33	0.20	0.83	>100	0.50
	1056.10.TA11.1826	2.0	1.1	0.68	0.20	>100	106	0.33	0.55	>100	0.18
	62357.14.D3.4589	0.29	0.19	0.10	0.083	0.029	4317	0.43	0.39	0.44	0.11
B	6240.08.TA5.4622	6.5	4.1	2.5	0.85	>100	71	1.6	0.60	1.2	0.39
	6244.13.B5.4567	0.57	0.95	0.65	0.26	1.2	340	0.16	0.12	0.21	0.072
	AC10.0.29	>100	1.9	1.0	>100	>100	79	1.6	1.1	8.3	0.63
	CAAN5342.A2	0.72	2.6	2.2	0.76	>100	38	0.45	0.55	>100	0.49
	SC05.8C11.2344	22	2.3	1.8	6.7	>100	95	0.26	0.49	>100	1.8
	TRO.11	0.47	0.17	0.14	0.065	>100	649	0.79	0.25	0.20	0.077
	WEAU.d15.410.787	1.81	0.27	0.20	0.098	>100	214	0.027	0.046	9.98	0.012
	CNE17	>100	3.7	2.6	5.93	>100	<20	0.18	0.38	>100	2.7
	CNE19	0.092	0.12	0.098	0.036	0.41	708	0.027	0.074	0.13	0.019
	CNE20	0.051	0.12	0.12	0.048	>100	1510	>100	1.3	>100	0.0078
BC	CNE21	0.25	0.55	0.50	0.19	>100	1668	0.58	0.078	2.0	0.016
	CNE30	6.1	3.2	2.6	0.99	3.6	441	0.60	0.28	0.45	0.14
	CNE52	0.87	0.60	0.41	0.11	1.2	630	0.10	0.084	0.092	0.025
	CNE53	0.10	0.11	0.069	0.057	0.061	11658	0.035	0.046	0.065	0.027
	CNE58	0.71	0.10	0.12	0.076	1.3	2315	0.49	0.087	>100	0.078
	1394C9G1(Rev.)	0.12	0.43	0.23	0.18	>100	465	1.2	0.15	0.058	0.058
	7030102001E5(Rev.)	12	1.4	0.91	0.39	>100	701	0.23	0.37	>100	0.22
	BF1266.431a	0.68	0.56	0.056	0.13	>100	355	0.0079	0.0077	>100	>100
	Ce0393 C3	0.15	0.38	0.26	0.15	>100	299	>100	0.12	0.015	0.013
	Ce0682 E4	>100	>100	>100	>100	>100	NT	>100	>100	>100	>100
	Ce1086 B2	>100	>100	>100	>100	>100	NT	>100	>100	>100	>100
	Ce1172 H1	>100	>100	>100	3.2	>100	<20	>100	>100	>100	>100
	Ce1176 A3	4.0	1.4	1.2	0.72	68	129	1.5	0.77	0.45	0.22
	Ce2010 F5	1.1	0.54	0.52	0.11	6.8	208	0.31	0.19	0.42	0.22
	Ce2060 G9	1.6	1.0	1.3	0.89	18	203	0.34	0.14	0.25	0.11
	Ce703010054 2A2	>100	2.0	2.8	2.4	>100	<20	0.99	0.43	>100	0.16
	Ce704809221 1B3	3.5	0.52	0.79	0.33	>100	58	0.32	0.24	0.29	0.20
	Du156.12	>100	0.088	0.11	0.18	>100	311	0.029	0.036	>100	0.22
	Du172.17	0.73	0.22	0.12	0.097	0.40	401	>100	>100	0.017	0.068
C	Du422.1	>100	>100	>100	>100	>100	31	>100	>100	>100	2.0
	HIV-0013095.2.11	1.7	0.52	0.56	0.46	>100	92	0.035	0.038	>100	0.048
	HIV-001428.2.42	0.45	0.062	0.058	0.077	>100	1077	0.0067	0.011	2.2	0.0087
	HIV-16055.2.3	0.65	0.24	0.20	0.26	>100	431	0.027	0.024	0.052	0.0087
	HIV-16845.2.22	>100	>100	>100	>100	>100	NT	>100	>100	>100	>100
	ZM109F.PB4	1.1	0.44	0.55	0.23	>100	<20	0.29	0.035	>100	0.21
	ZM135M.PL10a	2.2	2.0	2.3	0.62	>100	74	>100	0.57	>100	>100
	ZM197M.PB7	>100	0.53	0.66	0.55	>100	281	0.27	0.28	>100	>100
	ZM214M.PL15	1.3	1.7	2.2	0.43	1.7	199	0.48	0.36	0.31	0.085
	ZM233M.PB6	>100	4.0	2.3	>100	>100	57	>100	>100	>100	>100
	ZM246F.D5	>100	0.77	0.87	0.22	>100	<20	>100	2.2	>100	0.088
	ZM247F.F7	>100	>100	>100	>100	>100	<20	>100	0.16	>100	0.097
	ZM249M.B10	0.64	0.12	0.13	0.051	>100	301	0.044	0.056	>100	0.031
	ZM249M.PL1	0.78	0.16	0.11	0.062	>100	313	0.044	0.073	>100	0.060
	ZM53M.PB12	7.2	2.7	2.9	1.7	32	119	0.69	0.42	0.53	0.22
	3817.v2.c59	>100	2.6	3.4	3.1	>100	232	>100	>100	3.8	0.82
	6480.v4.c25	0.83	0.083	0.12	0.091	>100	416	0.031	0.029	0.090	0.016
CD	6811.v7.c18	1.5	0.86	1.1	0.72	>100	141	0.045	0.068	0.20	0.028
	6952.v1.c20	>100	0.17	0.16	0.55	>100	230	0.063	0.020	>100	>100
	89.F1 2 25	>100	>100	>100	>100	>100	NT	>100	>100	>100	>100
	191821 E6 1	>100	>100	>100	>100	>100	NT	>100	>100	>100	>100
D	231965.c01	>100	2.3	2.8	>100	>100	59	0.25	0.32	>100	>100
	3016.v5.c45	>100	0.24	0.36	4.4	>100	NT	1.7	0.068	0.23	0.048
	A07412M1.vrc12	0.23	0.29	0.18	0.18	>100	1037	0.11	0.088	0.027	0.016
	P0402 c2 11	0.99	0.54	0.54	0.46	>100	431	0.050	0.038	0.052	0.056
	P1981 C5 3	>100	4.27	4.14	15	>100	62	0.055	0.079	14	0.19
	X1193 c1	0.78	0.28	0.17	0.28	>100	344	0.0026	0.0013	6.46	0.066
G	X1254 c3	>100	1.39	1.10	>100	>100	68	0.019	0.024	>100	0.15
	X1632 S2 B10	0.70	1.25	1.14	1.03	>100	193	0.0028	0.0047	>100	0.055
	X2088 c9	>100	>100	>100	>100	>100	<20	>100	>100	>100	>100
	X2131 C1 B5	3.61	0.80	0.81	2.71	>100	411	0.0075	0.014	>100	0.26

Breadth (viruses neutralized)	Potency mAb (GeoMean IC_{50})	Potency Plasma (GeoMean ID_{50})
0-10%	10-50 μg/mL	/
10-25%	1-10 μg/mL	50-100
25-50%	0.1-1 μg/mL	100-300
50-75%	0.01-0.1 μg/mL	300-900
75-100%	<0.01 μg/mL	>900

Table S3. Neutralization IC50s of PCIN63 on a large cross-clade pseudovirus panel. – Related to Figure 1 and Table S4.

IC50 is defined as the Ab concentration in μg/mL allowing 50% loss of infectivity for the N=120 reference pseudoviurses (Seaman et al., 2010).

PCIN63 Abs are organized from least to most mutated.

Data are representative of at least 2 experiments.

Env Subtype	GeoMean (IC50 mg/mL) PCIN 66B	PCIN 71G	PCIN 71I	PCIN 71L	PCIN 77D	PC63 M33 serum	Min VRC01	Mat VRC01	Min 12A21	Mat 12A21
A	0.80	0.54	0.24	0.33	2.3	476	0.18	0.058	0.34	0.100
AC	0.21	0.18	0.15	0.086	0.097	30	0.45	0.042	0.014	0.018
ACD	0.12	0.48	0.63	0.032	/	1019	0.48	0.12	0.033	0.076
AE	2.7	0.64	0.48	0.59	0.18	234	0.83	0.28	0.14	0.087
AG	0.92	0.50	0.48	0.18	1.3	205	0.61	0.27	0.24	0.057
B	1.1	0.88	0.61	0.34	0.19	192	0.34	0.29	0.84	0.15
BC	0.33	0.40	0.33	0.18	0.68	1448	0.16	0.15	0.23	0.051
C	1.2	0.57	0.49	0.31	7.6	208	0.15	0.13	0.17	0.092
CD	1.1	0.42	0.53	0.58	/	237	0.044	0.034	0.41	0.072
D	0.23	0.54	0.56	0.90	/	246	0.36	0.12	0.079	0.028
G	1.2	0.98	0.85	1.4	/	191	0.012	0.014	1.7	0.11
Overall	0.93	0.58	0.46	0.34	1.1	255	0.21	0.13	0.25	0.084

Breadth (viruses neutralized)	Potency mAb (GeoMean IC_{50})	Potency Plasma (GeoMean ID_{50})
0-10%	10-50 μg/mL	/
10-25%	1-10 μg/mL	50-100
25-50%	0.1-1 μg/mL	100-300
50-75%	0.01-0.1 μg/mL	300-900
75-100%	<0.01 μg/mL	>900

Env Subtype	Breadth (% viruses) PCIN 66B	PCIN 71G	PCIN 71I	PCIN 71L	PCIN 77D	PC63 M33 serum	Min VRC01	Mat VRC01	Min 12A21	Mat 12A21
A	64%	82%	82%	73%	27%	100%	60%	70%	50%	70%
AC	50%	50%	50%	50%	25%	25%	50%	50%	25%	50%
ACD	50%	100%	100%	50%	0%	50%	100%	100%	50%	100%
AE	50%	86%	86%	86%	21%	79%	79%	86%	64%	79%
AG	67%	78%	78%	78%	22%	71%	56%	78%	67%	67%
B	92%	100%	100%	92%	17%	83%	100%	100%	58%	100%
BC	88%	100%	100%	100%	63%	88%	88%	100%	63%	100%
C	62%	79%	79%	79%	21%	77%	62%	76%	38%	72%
CD	40%	80%	80%	80%	0%	100%	60%	60%	60%	60%
D	25%	75%	75%	50%	0%	100%	75%	75%	50%	50%
G	57%	86%	86%	71%	0%	86%	86%	86%	43%	86%
Overall	63%	84%	84%	79%	21%	80%	72%	81%	51%	77%

Table S4. Neutralization breadth and potency of PCIN63 Abs on a large cross-clade pseudovirus panel. – Related to Figure 1 and Table S3.

PCIN63 Abs are listed vertically by increasing frequency of somatic hypermutations.

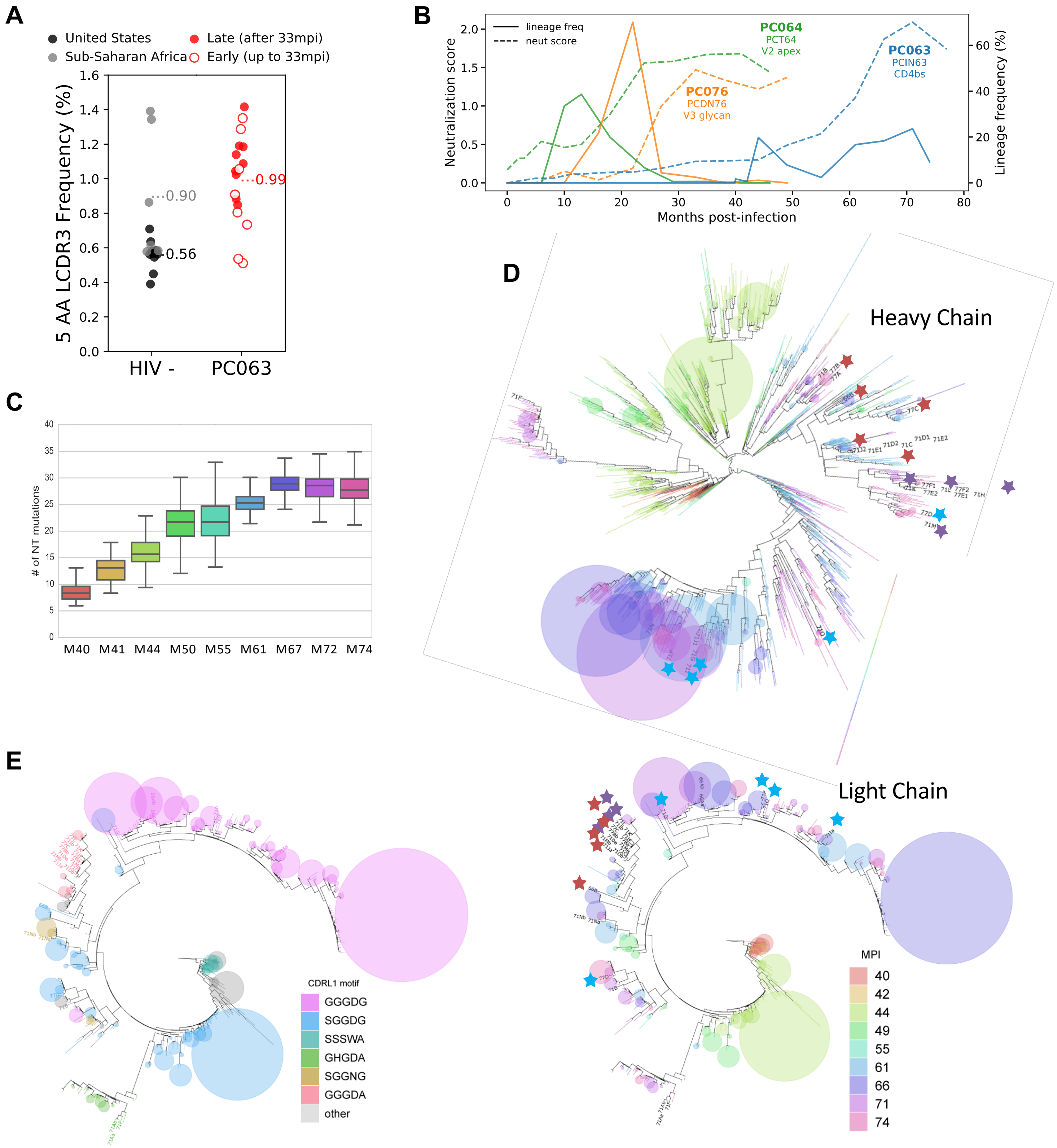

Figure S3. Emergence and development of the PCIN63 bnAb lineage – Related to Figure 2.

(A) Frequency of 5-amino acid LCDR3 immunoglobulin sequences in the naïve repertoire of PC63 (from Zambia) at different time points compared to HIV negative individuals from the United States (Southern California) and sub-Saharan Africa (Protocol C clinical research site in Zambia, Uganda, Rwanda and South Africa).

(B) Comparison of the PCIN63 Ab lineage (Blue) emergence and evolution with other bnAbs lineages isolated from two Protocol C participants (PC076: high-mannose patch targeting PCDN lineage in orange; PC064: V2-apex targeting PCT64 lineage in green) as detected in the periphery. Lineage sequences frequencies are plotted as a percentage of total lineage sequences from all time points. Plasma neutralization score (see (Landais et al., 2016)) from a heterologous medium panel is plotted as a dashed line. Related to Figure 2A.

(C) Somatic hypermutation frequency was calculated for each timepoint as divergence (number of nucleotide changes compared to LMCA). Data are presented as whisker plot showing mean, 95% upper and lower quartiles, standard deviation and outliers.

(D) Longitudinal phylogeny of PCIN63 heavy chain (Flea LINK) (Top) and light chain (Flea LINK) (Bottom) NGS sequences (colored by time-point, mpi). PCIN63 mAbs dependant (red) or accommodating (blue) the N276-glycan for neutralization or with intermediate phenotype (purple) are indicated with star symbols. Shaded circles represent the size of the cluster.

(E) PCIN63 light chain phylogeny as in (D) but colored by LCDR1-motif#4 amino acid sequence.

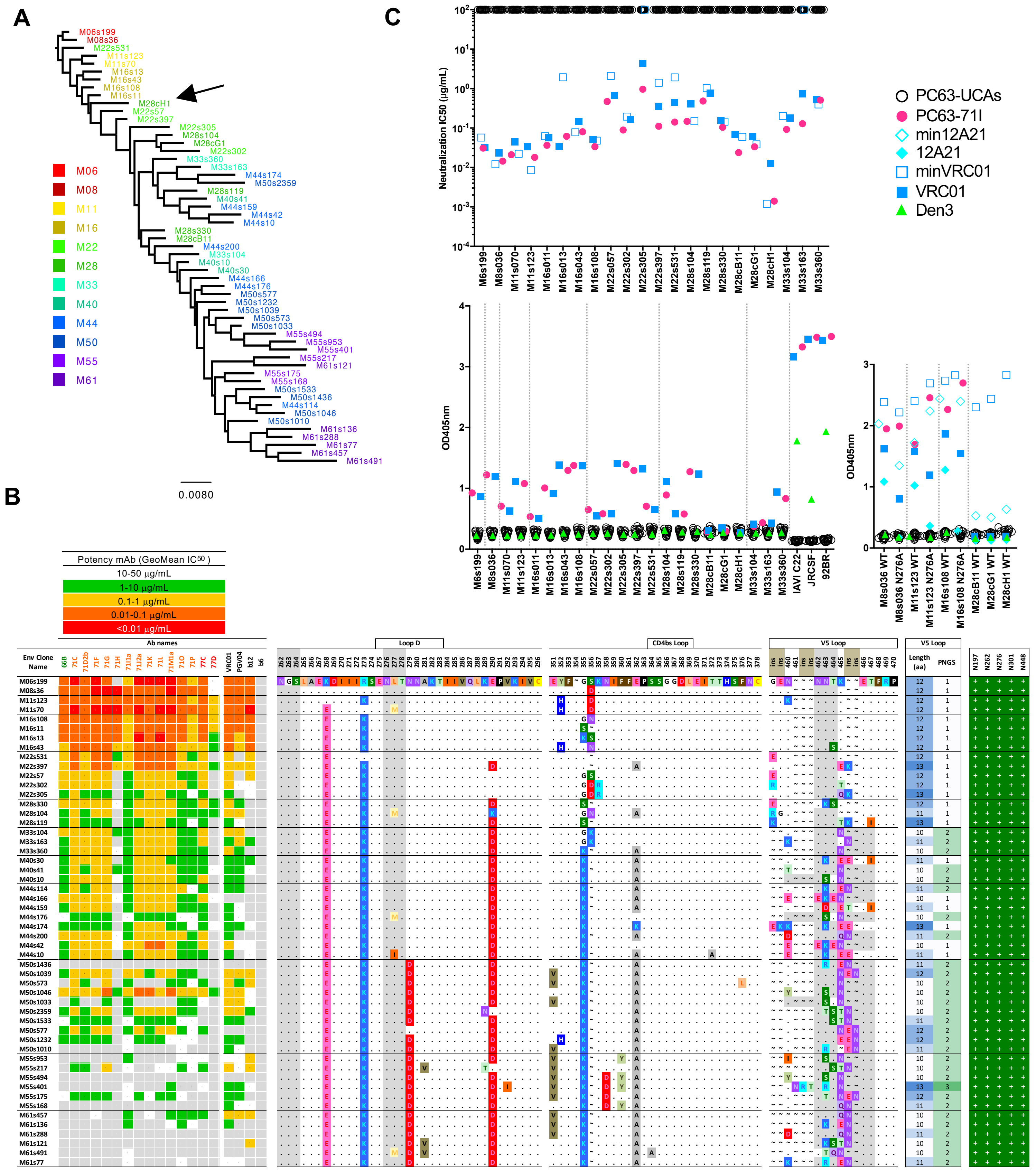

Figure S4. Elicitation of the PCIN63 lineage – Related to Figures 3 and 4.

(A) Phylogeny of PC063 HIV subtype C *env*, estimated by maximum likelihood from representative full-length *env* clonal sequences (c###) and PacBio high-quality consensus sequences (HQCS) (s###) obtained by next-generation sequencing of longitudinal plasma samples. Sequences are color-coded by time point (Months post infection, M). Clone M28cH1 is indicated with a black arrow.

(B) PCIN63 mAbs and control CD4bs Abs were tested for neutralization of pseudo-typed autologous PC063 Env clones representative of each time points (M-Months post infection, s-sequence number) as listed vertically. IC50s are tabulated and color coded as indicated in the legend. Amino-acid sequence and PNGS information of the PC063 autologous Env clones are indicated on the left. Amino-acid residues are numbered based on HxB2 alignment and color-coded as indicated in the legend. V5 loop length and number of PNGS are colored using a gradient.

(C) Putative PCIN63 UCA Abs (see Figure 3A) were tested for neutralization (Top) of representative pseudo-typed autologous Env clones (X-axis) selected from all time points prior to detection of the PCIN63 lineage in the periphery, and binding (bottom) to captured gp120 from corresponding pseudovirus stock lysates (left panel) or recombinantly expressed (right panel).

(B,C) Data are representative of at least 2 experiments.

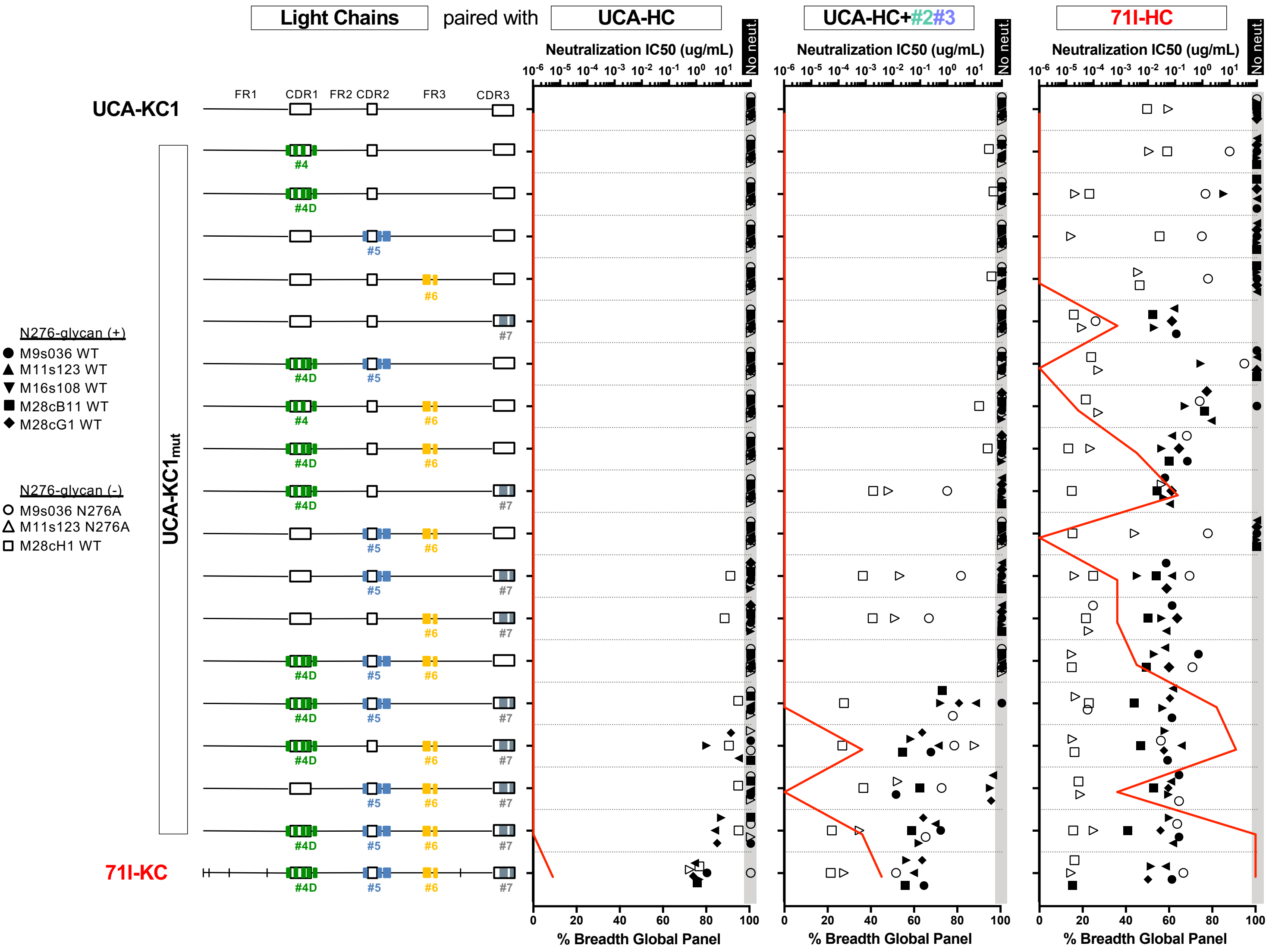

Figure S5. Functional evaluation of PCIN63 Ab lineage maturation – Related to Figure 4

PCIN63-71I-KC SHM motifs (as defined in Figure 2B) were introduced into the PCIN63-UCA-KC1, individually or in combination. Motifs are color-coded and their sequence position is indicated with a diagram. Mutated constructs were paired with WT PCIN63-UCA (left), PCIN63-UCA+Motif#2+Motif#3 (middle) or PCIN63-71I (right) HC. The chimeric Abs were tested for neutralization of the indicated WT and N276A autologous Env clones sensitive to neutralization by PCIN63-71I, as well as against the global 12-virus panel (De Camp et al., 2014). Data are representative of at least 2 experiments.

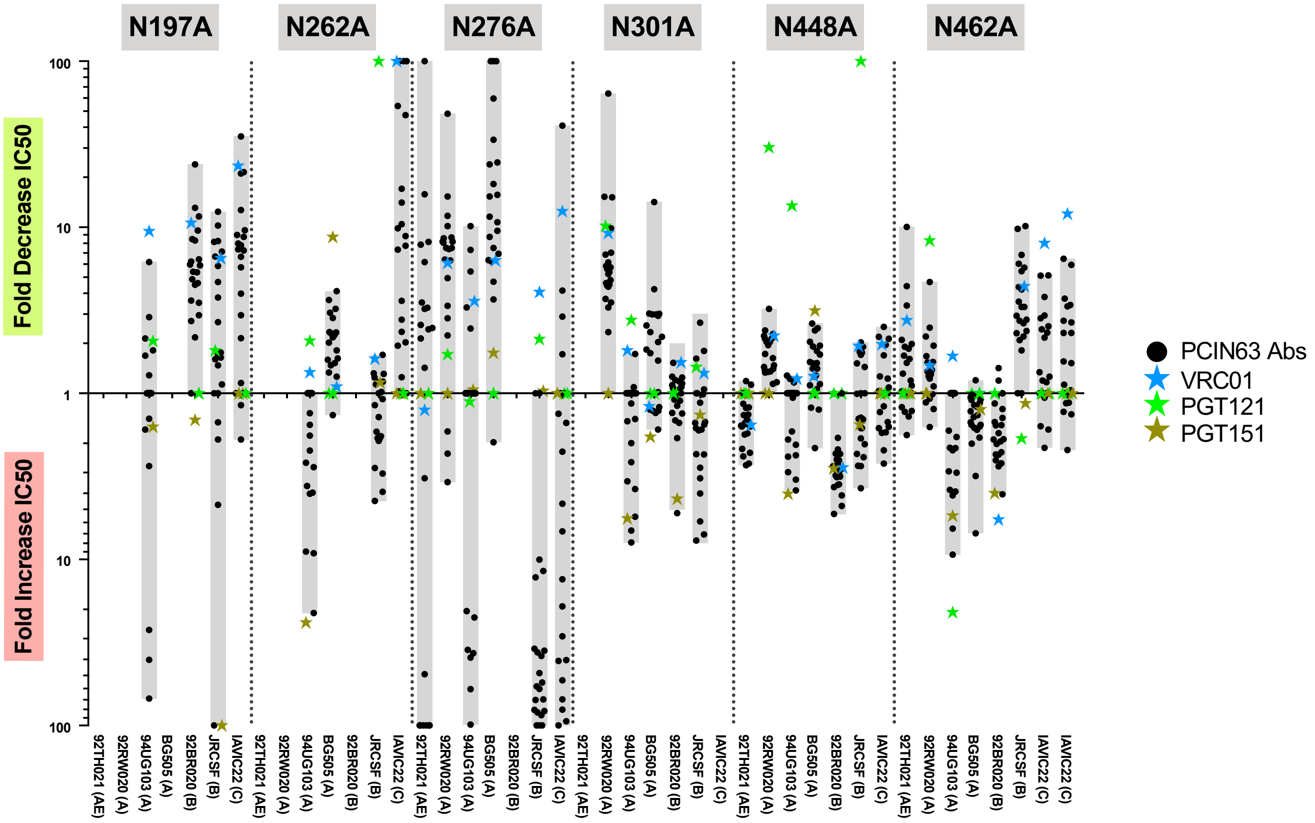

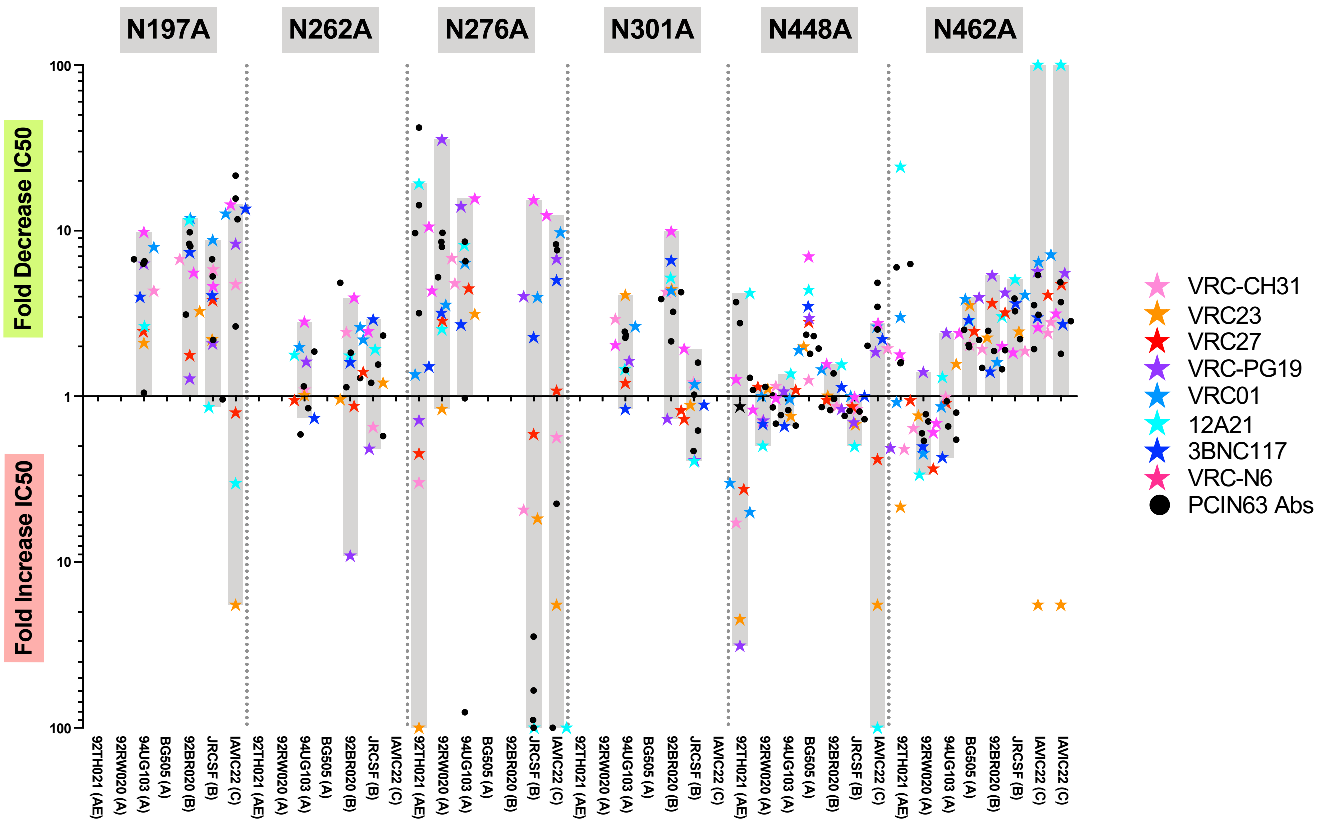

Figure S6. Effect of CD4bs N-glycan removal on neutralization by PCIN63 Abs – Related to Figure 5.
PCIN63 Abs, VRC01-class, PGT151 and PGT121 Abs were tested for neutralizing activity against the indicated pseudoviruses carrying the indicated mutation knocking down CD4bs surrounding N-glycan. The fold decrease or increase in neutralization IC_{50} is plotted. The gray bars indicate the range of variation for the PCIN63 Abs only (upper panel) or VRC01-class reference Abs (lower panel). Data are representation of at least two experiments.

Antibody	Sequence (CDR1, CDR2 marked above alignment)	CDR3
**VH1-2*02**	**QVQLVQSGAEVKKPGASVKVSCKASGYTFTG~~~~~~~~~YYMHWVRQAPGQGLEWMGWINPNSGGTNYAQ~KFQGRVTM~~TR~~~~DTSIS~~~TAYMELSRLRSDDTAVYYC**	
VRC01-HC	........GQM....E.MRI..R....E.ID.........CTLN.I.L...KRP.....LK.RG.AV...R.PL...............VYSD.....FL..RS.TV......F.	TRGKNCDYN~~~~~~~~~~~WDFEH
NIH45-46-HC	..R.S...GQM....E.MRL..R....E.LN.........CPIN.I.L...RRP.....LK.RG.AV...R..................VYSD.....FL..RS.T.......F.	TRGKYCTARDYYN~~~~~~~WDFEH
VRC02-HC	........GQM....E.MRI..Q....E.ID.........CTLN...L...RRP.....LK.RG.AV...R.PL...............VYSD.....FL..RS.TA........	TRGKNCDYN~~~~~~~~~~~WDFEH
VRC03-HC	.........VI.T..S...I..R....N.RD..........SI....LI.DK.F..I...K.LW.AVS..R.QL....S.....QLSQ.PDDPDWGV....F.G.TPA...E.F.	VRRGSCDYCGDFP~~~~~~~W~~QY
VRC06-HC	E....E..PVMR...S.M.I..AT...N.RD.........FSI....FNRRY.F..I...K.MW.AV...R.QL....S...S.LFSQ.LYYPDRG...L.F.G.T.A...D.F.	VRRGSSCPHCGD~~~~~~~~FHFEH
VRC07-HC	..R.S...GQM....D.MRI..R....E.IN.........CPIN.I.L...KRP.....MK.RG.AVS..R.QL...............MYSE.....FL..RS.T.......F.	TRGKYCTARDYYN~~~~~~~WDFEH
VRC08-HC	........TQM.E.....TI..VT...E.VE.........ILIN....V..R.......M..RG..V...R.Q...K............VYRD......LT..G.T.G...K.F.	VRGRSCCGGRRHCNGADCFNWDFQH
VRC18-HC	..R.....NQ.R......RI..E....K.ID.........HFI.....V..H....L.....RG..V..SRTS...KLS.TM.......NFEE......LD..K.NPG.....F.	ARGFAGY~~~~~~~~~~~~~EWSFI
VRC23-HC	...F..............R...E....S..D..........VLQ.I......RP......K.ER.AVS..P.Q....L.L.........LYTE.......HFKN.......I...	ARGVRRDAS~~~~~~~~~~~WWLQF
VRC27_HC	SQR.....PQ.R...S..RI..ET.....NA..........IL..F.....RSF......K.KF.AV...H.S....I.L.........IYRE.....FLD.TG..F........	ARDRLYDGSS~~~~~~~~~~WRLDP
VRC-CH31-HC	.........A.R......T....FAEDDDYSPYWVNPAPEHFI.FL......Q...LA.M..TN.AV...W.YLN...A..........R.MT.....FL.VKS...........	ARAQKRGRSE~~~~~~~~~~WAYAH
VRC-PG04-HC	........SG........R...WT.EDI.ER........TELI.............I..VKTVT.AV.FGSPD.RQ..SL.........RDLF.....H.DIRG.TQG...T.F.	ARQKFYTGGQG~~~~~~~~~WYFDL
VRC-PG19-HC	E.R...............R...A.......D.........FDI..L.....R.......VR.LG..VS..R.Q................FY.D.....F.DFRN.KM....L.F.	ARMGAARE~~~~~~~~~~~~WDFQY
VRC-PG20-HC	..H.M...T.M.......R.T.QT.....SD..........FI..L..V..R.F.....M..QW.QV...R.T................VYRE...V..LD.RS.TFA.....F.	ARRMRSQDRE~~~~~~~~~~WDFQH
VRC-N6-HC	RAH.....TAM.......R...QT......A.........HILF.F.....R....V...K.QY.AV.FGG.G.RD...L.........VYRE...I...DIRG.KP........	ARDRSYGDSS~~~~~~~~~~WALDA
12A12-HC	SQH.....TQ........RI..Q....S..D..........VL..W..............K.VY.AR...R.R....INF..D......IYRE...I.F.D..G.......L.F.	ARDGSGDDTS~~~~~~~~~~WHLDP
12A21-HC	SQH.....TQ........R...Q.......N..........IL..W.............LK.VF.AV...R.Q....IQL.........IYRE...I.FLD..G...........	ARDESGDDLK~~~~~~~~~~WHLHP
3BNC60-HC	..H.S....A.T......R...E....KISD.........HFI..W........Q.V.....KT.QP.NPR.Q.....SL....QASW.FDTY...SF..D.KAV......I.F.	ARQRSDF~~~~~~~~~~~~~WDFDV
3BNC117-HC	....L....A.T......R...E....NIRD..........FI..W........Q.V.....KT.QP.NPR.Q.....SL....HASW.FDTF...SF..D.KA.........F.	ARQRSDY~~~~~~~~~~~~~WDFDV
N60P25.1	H.......T...R.....RI..AS.....SN..........FI........R.......M..LR.AV..SG..................IYTE....SF.V..G.......I.FC	ARGRDGYE~~~~~~~~~~~~YGFNP
N49P7	~AD......V.....D..RI..E.Q..R.PD..........II..I.R.....P.....M..MG.QV.IPW.......S.............E.....FLD.RG.K........C	VRDRSNGSGKRFESSN~~~~WFLDL
DRVIA7	.....E..TQFRR.....R...E......IS.........SFI..I.G...........M..RH.AV...PRR...K...............D.........R........M.F.	VTSRTKDYDE~~~~~~~~~~WWFDP
PCIN63-71I	..................R..........NS.........CLI..W........Q..A. ..LH.AV...H.Q....I.V............D......RG.......T...T..	TRDASRDDRA~~~~~~~~~~WRLDP

Antibody	Sequence (CDR1, CDR2 marked above alignment)	CDR3
**VK3-20*01**	**EIVLTQSPGTLSLSPGERATLSCRASQS~~VSSSYLAWYQQKPGQAPRLLIYGASSRATGIPDRFS~GSGSGTDFTLTISRLEPEDFAVYYC**	
VRC01-KC	.................T.II...T..Y..~~~GS......R.......V..SG.T..A..........RW.P.YN....N..SG..G....	QQYEF
NIH45-46-KC	........A........T.II...T.....~~~GS......R.......V..SG.T..A..........RW.A.YN.S..N..SG..G....	QQYEF
VRC02-KC	.................T.II...T..Y..~~~GS......R.......V..SG.T..A..........RW.P.YN...RN..SG..GL...	QQYEF
VRC03-KC	.........I.......T...F.K...G..~~GNAMT...KRR..V......DT.R..S.V....V.........F...NK.DR........	QQFEF
VRC06-KC	........A..................G..~~GNS.N...KRR..T......DT.R..SD..EK.V.........S...TKVG.........	QQFEF
VRC07b-KC	........A..........I....T..Y..~~~GS......R.......V..AG.T.............RW.AEYN....N..S...G....	QQYEF
VRC08-KC	Y.GV....AI..V.L...V....KT..A..ITPRH.V.HR..G....S.VMT.T.E..S......I.............T...A........	QCLEA
VRC18b-KC	.................T......T..G..IL.NQ...H..RR..P.......G.N..P...E..T.........V...K...RD.......	QILEF
VRC-PG04-KC	.................T.S...T.ASY..~~~GHMT...K....P.K...FAT.K..S..........QF.KQY....T.M......R...	QQLEF
3BNC60-KC	D.QM....SS..ARV.DTV.IT.Q.NGY..~~~~~.N....RR.K..K....DG.KLER.V.A.....RRW.QEYN...NN.Q...V.T.F.	QVYEF
3BNC117-KC	D.QM....SS..A.V.DTV.IT.Q.NGY..~~~~~.N....RR.K..K....DG.KLER.V.S.....RRW.QEYN...NN.Q...I.T.F.	QVYEF
**VK3-15*01**	**EIVMTQSPATLSVSPGERATLSCRASQS~~~VSSNLAWYQQKPGQAPRLLIYGASTRATGIPARFS~GSGSGTEFTLTISSLQSEDFAVYYC**	
VRC23-KC	........V.V...R.GT.........G....G.DV....H....T............S.V.E.......FHVD...S..G..P..V.I...	QQYET
**VK1-33*01**	**DIQMTQSPSSLSASVGDRVTITCQASQD~~~ISNYLNWYQQKPGKAPKLLIYDASNLETGVPSRFS~GSGSGTDFTFTISSLQPEDIATYYC**	
VRC27-KC	...........AV...G......R...G....GSD.H.....R.RP..I..HH..AR.E......G.....H.P.I...ND..LD.V.....	QVIES
VRC-CH31-KC	..............L............RG....GKD.......A.......VS...T..G...........FHQN.SL......A..V...F.	QQYET
VRC-N6-KC	Y.HV........V.I......N..T..G...VGSD.H...H...R......HHT.SV.D...........FH.S.NL...D..AD.......	QVLQF
12A12-KC	.........................G.G....GSS.Q.............VHG....HR...........FH.T.SL...G..RD.F...F.	AVLEF
12A21-KC	.....................N...G.G....GSS.....K...R.....VHG....QR...........FH.T..L.......D.V...F.	AVFQW

Antibody	Sequence (CDR1, CDR2 marked above alignment)	CDR3
**VL2-14*01**	**QSALTQ PASVSGSPGQSITISCTGTSSDVGGYNYVSWYQQHPGKAPKLMIYEVSNRPSGVSNRFS~GSKSGNTASLTISGLQAEDEADYYC**	
VRC-PG19-KC	........................~A...FR.FSS......V..R....L.FS.NR....I.H....................I......H.	NAYEF
VRC-PG20-KC	........P...........L....A.~~~~~~TS.A....YAD...R.IVFDGNK...DI.S......Q..G..........S....Y.H.	NAFEF
**VL2-11*01**	**QSALTQ PRSVSGSPGQSVTISCTGTSSDVGGYNYVSWYQQHPGKAPKLMIYDVSKRPSGVPDRFS~GSKSGNTASLTISGLQAEDEADYYC**	
N49P7	............A.............HN~~~~~~L....C.HQ..R....L...FN..........G..G......T...DD.D.E.F.	WAYEA
**VK1-5*01**	**DIQMTQSPSTLSASVGDRVTITCRASQS~~~ISSWLAWYQQKPGKAPKLLIYKASSLESGVPSRFS~GSGSGTEFTLTISSLQPDDFATYYC**	
DRVIA7	........V.....I............R....DN.V.......R...........I.T..................S.N......V.....	QQFEE
N60P25.1	YV.~.......Y............S......~~G.K................S.T.............D.....N...T..........F.	QHFET
PCIN63-71I	........A...........L....G.G....G.D.G..............H...N.IN...........FH.......N............	QVFEW

Table S5. VRC01-class Abs aminoacid sequence aligment with their putative germline V-gene– Related to Figure 5 and S6

CDRH3 Sequences are highlighted in grey. Motifs (as defined in Figure 2) are highlighted in the corresponding colors.
