# Supplementary material for: Rapid and Focused Maturation of a VRC01-Class HIV Broadly Neutralizing Antibody Lineage Involves Both Binding and Accommodation of the N276-Glycan
Source: Immunity. 2019 Jul 16;51(1):141–154.e6. doi: 10.1016/j.immuni.2019.06.004 (PMC6642152; doi:10.1016/j.immuni.2019.06.004)
Supplement: Document S2. Article plus Supplemental Information [file mmc2.pdf]

Immunity

Article

Rapid and Focused Maturation of a VRC01-Class HIV Broadly Neutralizing Antibody Lineage Involves Both Binding and Accommodation of the N276-Glycan

Graphical Abstract

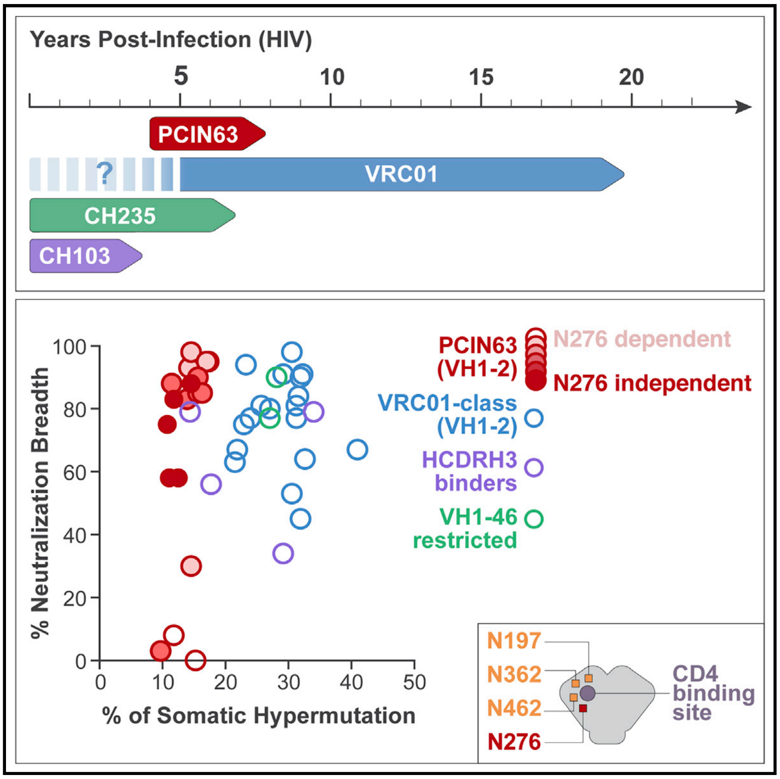

Authors

Jeffrey Umotoy, Bernard S. Bagaya, Collin Joyce, ..., Bryan Briney, Devin Sok, Elise Landais

Correspondence

dsok@iavi.org (D.S.), elandais@iavi.org (E.L.)

In Brief

Understanding the molecular basis of HIV Env-specific broadly neutralizing antibodies (bnAbs) development especially, at early stages, is key for germline-targeting vaccine design strategies. Umotoy et al. mapped the development of a VRC01-class bnAb lineage that achieved breadth in 2 years, revealing early binding to the N276-glycan during affinity maturation, which may have implications for vaccine design.

Highlights

- Isolation of PCIN63, a VRC01-class CD4-binding site Ab lineage with only 12% SHM
- PCIN63 lineage emerged at 40 months post infection and achieved breadth in 2 years
- Identification of a putative PCIN63 UCA reveals the importance of the CDRL3
- PCIN63 bnAbs segregate in N276 glycan- dependent and -independent sub-families

Umotoy et al., 2019, Immunity *51*, 141–154
July 16, 2019 © 2019 The Authors. Published by Elsevier Inc.
https://doi.org/10.1016/j.immuni.2019.06.004

Immunity
Article

CellPress

Rapid and Focused Maturation of a VRC01-Class HIV Broadly Neutralizing Antibody Lineage Involves Both Binding and Accommodation of the N276-Glycan

Jeffrey Umotoy,[1,2] Bernard S. Bagaya,[3,4] Collin Joyce,[5] Torben Schiffner,[5,6] Sergey Menis,[1,2] Karen L. Saye-Francisco,[5] Trevor Biddle,[5] Sanjay Mohan,[7] Thomas Vollbrecht,[7] Oleksander Kalyuzhniy,[1,2] Sharon Madzorera,[8] Dale Kitchin,[8] Bronwen Lambson,[8] Molati Nonyane,[8] William Kilembe,[9] The IAVI Protocol C Investigators and The IAVI African HIV Research Network, Pascal Poignard,[1,5,10] William R. Schief,[1,5,6] Dennis R. Burton,[1,5,6,11] Ben Murrell,[7,12] Penny L. Moore,[8,13,14] Bryan Briney,[5,6] Devin Sok,[1,2,5,6,*] and Elise Landais[1,2,5,15,*]

[1]International AIDS Vaccine Initiative Neutralizing Antibody Center, La Jolla, CA 92037, USA
[2]International AIDS Vaccine Initiative, New York, NY 10004, USA
[3]UVRI-IAVI HIV Vaccine Program, Entebbe, Uganda
[4]Department of Immunology and Molecular Biology, School of Biomedical Sciences, College of Health Sciences, Makerere University, Kampala-Uganda
[5]Department of Immunology and Microbiology, The Scripps Research Institute, La Jolla, CA 92037, USA
[6]Center for HIV/AIDS Vaccine Immunology and Immunogen Discovery (CHAVI-ID) The Scripps Research Institute, La Jolla, CA 92037, USA
[7]Department of Medicine, University of California San Diego, San Diego, CA 92103, USA
[8]Centre for HIV and STIs, National Institute for Communicable Diseases, of the National Health Laboratory Service (NHLS), Johannesburg 2131, South Africa
[9]Rwanda-Zambia HIV Research Group, Lusaka & Ndola, Zambia
[10]Institut de Biologie Structurale, Université Grenoble Alpes, Commissariat a l'Energie Atomique, Centre National de Recherche Scientifique and Centre Hospitalier Universitaire Grenoble Alpes, 38044 Grenoble, France
[11]Ragon Institute of Massachusetts General Hospital, Massachusetts Institute of Technology, and Harvard, Cambridge, MA 02114, USA
[12]Department of Microbiology, Tumor and Cell biology, Karolinska Institutet, Stockholm, Sweden
[13]School of Pathology Faculty of Health Sciences, University of the Witwatersrand, Johannesburg 2050, South Africa
[14]Centre for the AIDS Programme of Research in South Africa (CAPRISA), University of Kwa-Zulu Natal, Durban 4013, South Africa
[15]Lead Contact
*Correspondence: dsok@iavi.org (D.S.), elandais@iavi.org (E.L.)
https://doi.org/10.1016/j.immuni.2019.06.004

SUMMARY

The VH1-2 restricted VRC01-class of antibodies targeting the HIV envelope CD4 binding site are a major focus of HIV vaccine strategies. However, a detailed analysis of VRC01-class antibody development has been limited by the rare nature of these responses during natural infection and the lack of longitudinal sampling of such responses. To inform vaccine strategies, we mapped the development of a VRC01-class antibody lineage (PCIN63) in the subtype C infected IAVI Protocol C neutralizer PC063. PCIN63 monoclonal antibodies had the hallmark VRC01-class features and demonstrated neutralization breadth similar to the prototype VRC01 antibody, but were 2- to 3-fold less mutated. Maturation occurred rapidly within ∼24 months of emergence of the lineage and somatic hypermutations accumulated at key contact residues. This longitudinal study of broadly neutralizing VRC01-class antibody lineage reveals early binding to the N276-glycan during affinity maturation, which may have implications for vaccine design.

INTRODUCTION

Elicitation of broadly neutralizing antibodies (bnAbs) targeting the HIV envelope glycoprotein (Env) is thought to be a key component of a successful HIV-1 vaccine (Fauci, 2017). VRC01-class antibodies, which target the conserved CD4 receptor binding site (CD4bs), are among the broadest neutralizing antibodies. However, these bnAbs typically display high levels of somatic hypermutation (SHM) (Falkowska et al., 2012; Huang et al., 2016; Scheid et al., 2011; Wu et al., 2010; Zhou et al., 2015) and often require years to develop during natural infection (Landais et al., 2016; Lynch et al., 2012). These features suggest that VRC01-class antibodies undergo a long and complex affinity maturation process (Wu et al., 2015) and may be difficult to elicit by immunization.

VRC01-class antibodies have been isolated from several chronically HIV-infected individuals and differ by up to 42% in nucleotide sequence. However, antibodies of this class share common features (Wu et al., 2010; Zhou et al., 2015) (Huang et al., 2016; Sajadi et al., 2018) including the use of a VH1-2 variable gene, a 5-residue LCDR3, and a short/flexible LCDR1. These shared features favor the rational design of immunogens to activate the precursors of VRC01-class bnAbs—so called germline-targeting immunogens (Jardine et al., 2013; McGuire et al., 2013). Such immunogens have succeeded in eliciting

This is an open access article under the CC BY-NC-ND license (http://creativecommons.org/licenses/by-nc-nd/4.0/).

narrowly neutralizing antibody responses with VRC01-class features in transgenic mouse models (Briney et al., 2016b; Dosenovic et al., 2015; Jardine et al., 2015; McGuire et al., 2016; Sok et al., 2016; Tian et al., 2016). However, these responses lack the neutralization breadth associated with VRC01-class bnAbs isolated from chronic infection. Comparison between VRC01-class antibodies (Briney et al., 2016b; Jardine et al., 2013; McGuire et al., 2013) and subsequent design of minimally mutated VRC01-class antibodies (Jardine et al., 2016b) highlighted the functional role of key "patches" of SHM that contribute to neutralization breadth and potency. The importance of these mutations was confirmed by comparing to the relatively strain-specific neutralizing DRVI07 antibody lineage, which harbored all the distinguishing features of VRC01-class antibodies except for the SHM in the light chain needed to accommodate the N276- and N462- glycans adjacent to the CD4bs (Kong et al., 2016). These data indicate that accommodation of the glycans surrounding the CD4bs is a major hurdle for acquiring neutralization breadth that is typical for VRC01-class antibodies.

A detailed analysis of VRC01-class antibody development during infection has been limited by the rare nature of these responses during natural infection and the lack of longitudinal sampling of such responses. Furthermore, although germline-targeting immunogens have successfully fished out naive precursors B cells with VRC01-like features from HIV-naive individuals (Jardine et al., 2016a; Havenar-Daughton et al., 2018), whether these precursors are capable of leading to bnAbs is not known. Moreover, there is no clear pathway for the rapid elicitation of VRC01-class lineages and it is not known whether key mutations need to be introduced in a particular order.

In this study, we describe and map the rapid development of VRC01-class bnAbs in a subtype C-infected Protocol C participant, PC063, with clear CD4bs-targeting broadly neutralizing plasma activity (Landais et al., 2016). We report characterization of monoclonal antibodies isolated from this donor and outline the affinity maturation of the antibody lineage through next-generation sequencing and functional analyses. Overall, the elicitation and affinity maturation of VRC01-class antibodies in the PC063 donor challenges the notion that VRC01-class antibodies require high levels of somatic hypermutation and long periods of affinity maturation to gain neutralization breadth and potency. Additionally, we present data that suggests, in the case of the PCIN63 lineage, the presence of the N276 glycan adjacent to the CD4bs that commonly obstructs VRC01-class antibody binding might have offered favorable interactions to drive affinity maturation of this antibody lineage. The results of these findings have direct implications for HIV vaccine design strategies.

RESULTS

PCIN63 Antibodies Are Minimally Mutated VRC01-Class bnAbs

Participant PC063 from the Protocol C cohort was shown previously to develop a CD4bs-directed bnAb response (Landais et al., 2016). Broadly neutralizing antibody activity was first detected in PC063 plasma at 54 months post infection (mpi), approximately 2 years later than observed for most Protocol C broad neutralizers and reached peak neutralization at 72 mpi (Figure 1A). To isolate the antibodies contributing to the plasma neutralization breadth in this donor, we used the previously described recombinant HIV Env (rgp140F) WT and D368R (CD4bs epitope knock-out) proteins (Li et al., 2012). These proteins differentially adsorbed the broadly neutralizing activity from the plasma (Figure S1A) and were used as fluorophore-conjugated baits to sort CD4bs-specific memory B cells from peripheral blood mononuclear cells (PMBCs) samples collected at 66, 71, and 77 mpi (Figure 1A). Using this sort strategy, 18 mAbs, which define the PCIN63 lineage, were isolated (Figure S1B).

PCIN63 Abs derive from IGHV1-2*02/IGHJ5*02 and IGKV1-5*03/IGKJ1*01 genes, and exhibit characteristic features of VRC01-class bnAbs, including a 5-residue LCDR3 QxxEx motif, a flexible Gly-rich LCDR1 and a 15-residue HCDR3 containing a WxxxDx motif upstream of HFR4 (Figure 1B, S1C, S1D, S2). Importantly, the SHM frequency of the PCIN63 lineage ranges from 9.6% to 16.0% (V_H+J_H) and from 10.0% to 13.7% (V_K+J_K) nucleotide mutation for the heavy (HC) and light (LC) chains, respectively, which is 2- to 3-fold lower than other VRC01-class bnAbs (Figures 1B and S1C). Based on an alignment of PCIN63 mAbs amino acid sequences, the SHMs for this lineage accumulated at positions previously shown to be key epitope contact residues for the VRC01-class of bnAbs. These include mutations in HCDR1 and HCDR2, which are important for high-affinity binding to the gp120-Loop D and CD4bs-loop, and mutations in LCDR1 and LFR3 which reduce the steric clash with the N276- and N462-glycans on Env (Figure S2) (Zhou et al., 2015; Jardine et al., 2016b). Moreover, the neutralization breadth of PCIN63 bnAbs is equivalent to VRC01 on a large cross-clade panel of pseudoviruses (N = 134) despite lower mutation frequencies (Figures 1B, 1C, Tables S1-S4). In fact, SHM in PCIN63 mAbs matched closely that of an engineered variant of mAb 12A21 designed to have the minimal set of SHM required for broadly neutralizing activity (min12A21) (Figure S2) (Jardine et al., 2016b). The identification of PCIN63 Abs thus challenges previous notions that high levels of SHM and prolonged affinity maturation are required for neutralization breadth and potency.

The Rapid Development of the PCIN63 bnAb Lineage Is Not Due to High Naive B Cell Precursor Frequency Nor Biased AID Motifs

The development of VRC01-class antibodies responses is rare compared to bnAbs targeting other epitopes (Li et al., 2007; Walker et al., 2010; Lynch et al., 2012, Landais et al., 2016; Rusert et al., 2016). To determine whether the elicitation of VRC01-class antibodies in this donor was favored because of a higher frequency of VRC01-class precursors, we performed next-generation sequencing (NGS) of the naive peripheral B cell repertoire. The frequency of 5-residue LCDR3 B cells in the naive repertoire of PC063 was found to be slightly higher compared to HIV-naive donors from the United States (California) but similar to HIV-negative individuals from other African Protocol C sites (Figure S3A).

We next performed NGS on the peripheral IgG$^+$ B cell repertoire to determine whether the low frequency of SHM observed for PCIN63 might have been associated with the presence or absence of biased AID motifs in the antibody lineage sequences that would favor affinity maturation. A total of 17 time

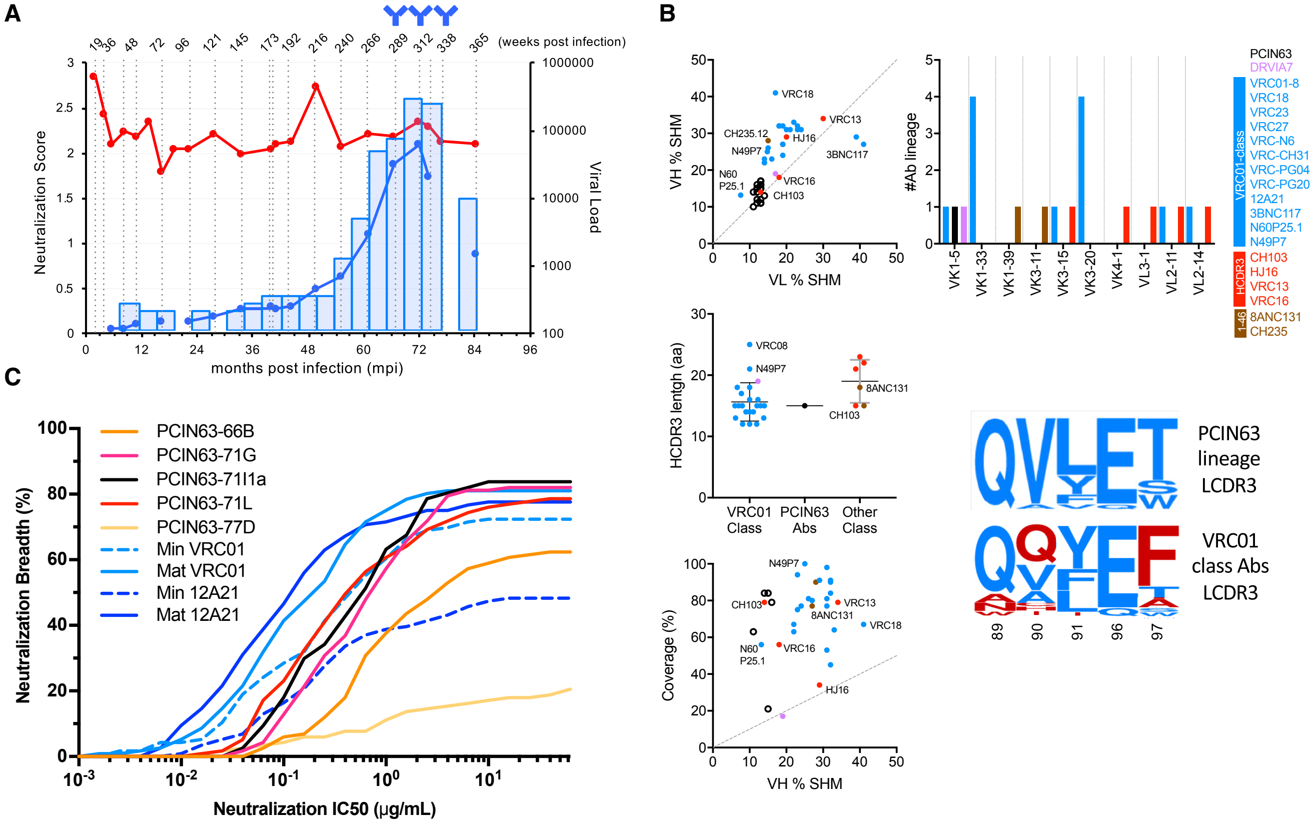

Figure 1. PCIN63 Antibodies Define a Minimally Mutated VRC01-Class bnAb Lineage

(A) Longitudinal plasma samples from donor PC063 were tested for neutralization against heterologous pseudoviruses. The percent of viruses neutralized (> 50% inhibition of infectivity at the lowest plasma dilution, 1:50) from a cross-clade (A, B, C) 37-virus panel is shown as shaded blue bars. The blue line represents the neutralization score calculated for each sample and taking into account breadth and potency (see Landais et al., 2016). The evolution of the viral load (red circles) in the plasma is also plotted. The time points at which PCIN63 antibodies were isolated are indicated by antibody symbols. See also Figure S1A and S1B.

(B) Comparison of CD4bs Abs genetic characteristics. Antibodies are listed and color-coded by classes as defined in Zhou et al., 2016: PCIN63 (Black), DRVI07 (Violet), VRC01-class bnAbs (Blue), HCDR3 dominated bnAbs (Red), VH1-46+ bnAbs (Brown). Top left inlet shows heavy (VH) and light chains (VL) putative V-genes nucleotide variation from IMGT database reference (% SHM). Middle left inlet shows length the HCDR3s, with mean ± SEM showed as lines. Bottom left inlet shows heterologous neutralization breadth (coverage in percentage of viruses neutralized) as reported in CATNAP database (www.hiv.lanl.gov/content/immunology/neutralizing_ab_resources.html) and heavy chains (VH) V-genes nucleotide variation from IMGT database reference (percentage of SHM). Top right inlet shows light chain V-gene usage. Bottom right inlet shows logogram of the LCDR3 amino acid sequences for PCIN63 and other VRC01-class Abs. Residues common or unique to each category are colored in blue and red, respectively. See also Figure S1C, S1D, and S2.

(C) Neutralization breadth (percentage of virus neutralized) is plotted as a function of potency (neutralization IC_{50} in μg/mL) of the indicated antibodies on a 120-virus panel (Seaman et al., 2010). See also Tables S1–S4. Data are representative of at least two experiments.

points between 4 and 77 mpi were processed to generate unpaired heavy-chain and light-chain sequences (Figure S3). The PCIN63 lineage was first detected at 40 mpi with progressive accumulation of nucleotide mutations that plateaued at 67 mpi (Figure S3C). Although the emergence of the PCIN63 bnAb lineage in PC063 occurred almost 2–3 years later than other bnAb lineages from published co-evolution studies targeting the CD4bs (CH235, non-VRC01-class) (Bonsignori et al., 2016), as well as other epitopes, the time required to reach maximum plasma breadth was overall similar (Figures 2A and S3B). Delayed onset (between 37–42 mpi) and fast maturation (14–19 mpi) to breadth was also reported for the DH270, which is a V3-glycan targeting lineage (Bonsignori et al., 2017). While comparison between independent studies is difficult to do accurately and plasma breadth does not necessarily correlate with frequency of SHM in the bnAb lineage, we verified that our estimation of time to maturation was overall consistent with the time to acquire some neutralization breadth in the earliest mAbs isolated from these lineages. We next evaluated whether the regions encoding for key contacts to HIV Env had an enrichment of AID hotspots that might have favored this rapid and highly focused affinity mutation to gain neutralization breadth, but no significant enrichments were found (Rogozin et al., 2001, See methods). Overall, despite the relatively rapid development of VRC01-class antibodies in the PC063 donor, high precursor frequencies or biased AID motifs that might have accelerated affinity maturation and selection does not appear to be driving factors.

Longitudinal Sequence Analysis Identifies the Emergence of SHM Motifs Associated with Neutralization

Longitudinal evaluation of antibody lineages in natural infection can provide information on the order that SHM accumulates,

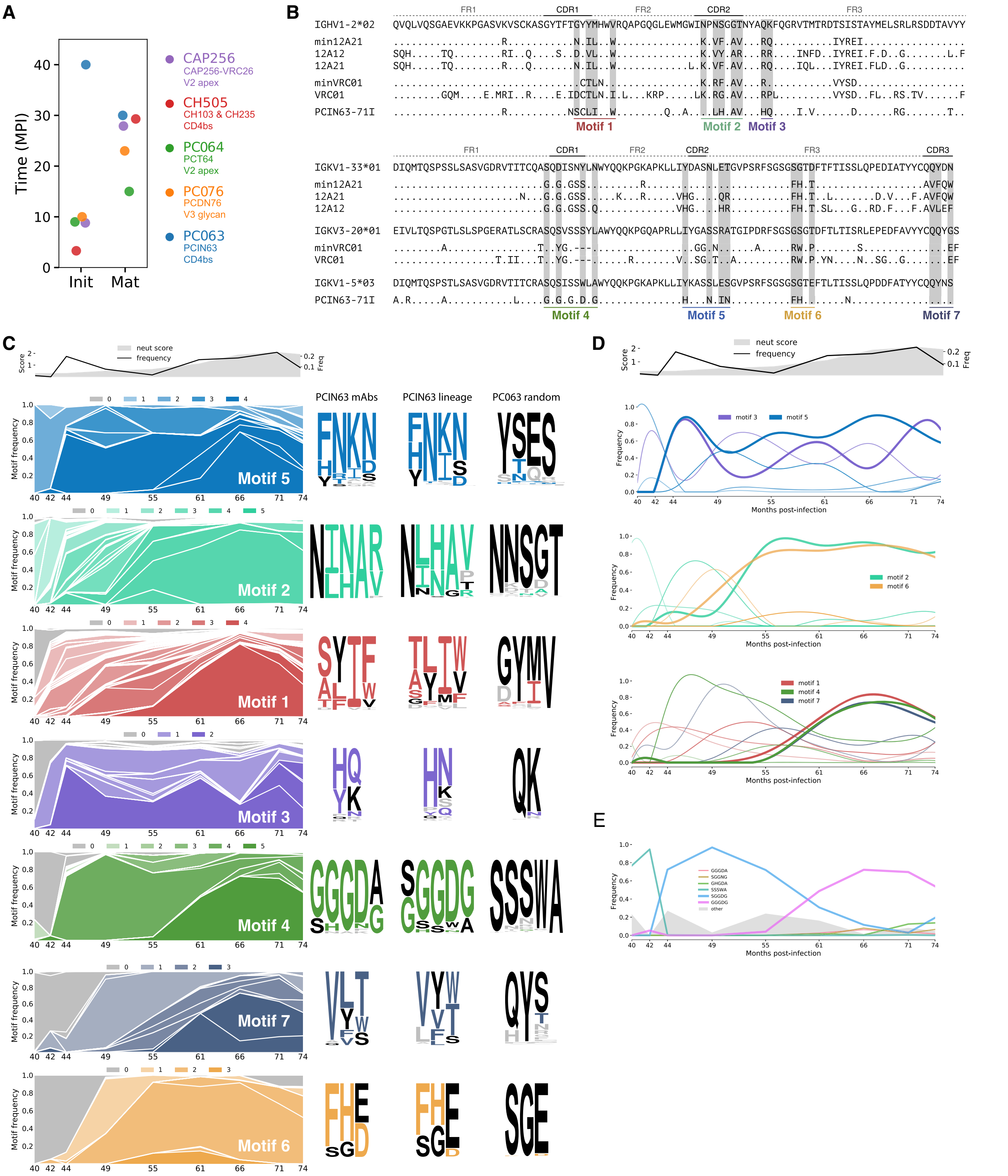

Figure 2. SHM Motifs Are Selected Sequentially in the PCIN63 bnAb Lineage

PC063 IgG libraries prepared from total PBMCs (a single vial of 10 million cells per time point) were amplified with IgG-specific primers for all human VH gene families. See also Figure S3.

(A) Comparison of the PCIN63 Ab lineage (Blue) development kinetics with other bnAbs lineages isolated as reported in original publications. Time to maturation (Mat.) is defined as the time between first detection of the lineage in the periphery (Init.) and peak neutralization breadth in plasma. See also Figure S3B.

(legend continued on next page)

which can then inform rational vaccine strategies to elicit a similar sequence of events. Accordingly, for PC063, detailed analysis of SHM selection pathways could serve as template for elicitation of VRC01-class Abs. Thus, we tracked the emergence and evolution of seven PCIN63 SHM motifs over the course of infection (Figure 2B). These motifs were selected based on previous studies identifying the minimal SHM required for VRC01 and 12A21 broad neutralization (Jardine et al., 2016b). HCDR2-motif #2 (contacting CD4bs-loop) and LCDR2-motif#5 were first selected for, with mutations detected in nearly all sequences at month 40 at the emergence of the lineage (Figure 2C). Selection of HCDR1-motif#1 (stabilizing HCDR2) and HFWR3-motif#3 (contacting V5 loop) residues quickly followed at month 42, then LCDR1-motif#4 (adaptation to N276-glycan) at 44 mpi and finally HFWR3-motif#6 (adaptation to N276-glycan) and LCDR3-motif#7 (contacting Loop D) at 49 mpi (Figure 2C). Both HCDR2-motif #2 and HCDR1-motif#1 sampled a diversity of mutations between months 42 and 55 before converging on a final set of mutations at month 55 and month 66, respectively. In contrast, for LCDR2-motif#5 and HFWR3-motif#3, the sequences rapidly converged at month 44 to a predominant sequence, which then persisted through month 74. While LCDR3-motif#7 sampled several sequences before convergence at month 66, the other sets of mutations in the light chain, LCDR1-motif#4 and LFWR3-motif#6, converged rapidly without sampling different combinations of mutations.

Another representation of the data is presented in Figure 2D, which emphasizes when each SHM sequence motif fully converged to the affinity-mature SHM sequence. Globally, there appears to be three phases, early convergence for HCDR2-motif#3 and LCDR2-motif#5, intermediate convergence for HCDR2-motif#2 and LFWR3-motif#6 and late convergence for HCDR1-motif#1, LCDR1-motif#4, LCDR3-motif#7 (Figures 2D and 2E). Overall, these results convey a complex affinity maturation process where different sequences are sampled for each SHM motif that then become fixed at different time points, in response to changes in the autologous virus swarm over time.

The PCIN63-UCA Does Not Bind to Env Proteins from Time Points Preceding Initiation of the Lineage

To better define the affinity maturation pathway for the PCIN63 lineage, we next determined the PCIN63 unmutated common ancestor (UCA). For the HC, an unmutated common ancestor (PCIN63-UCA-HC) was identified from the 40 mpi time point. However, for the light chain, lineage assignment based solely on V_K+J_K gene usage and 5-residue LCDR3 criteria identified 18 unmutated putative precursor light chain sequences from the NGS dataset. The most frequent putative unmutated light chain sequence had an LCDR3 with amino-acid sequence QQSEA and was named PCIN63-UCA-KC1 (Figure 3A). The second most frequent unmutated light chain sequence with matching V_K+J_K genes had an LCDR3 with amino-acid sequence QLYET, similar to the LCDR3 of the least mutated PCIN63-light chain sequences found at 40 mpi and was named PCIN63-UCA-KC2 (Figure 3A). To functionally characterize these putative UCAs, all 18 light chain variants were subsequently paired with the PCIN63-UCA-HC (UCA1-UCA18) and first tested for binding to VRC01-class germline targeting immunogens (Figure 3B). Only three Abs, including UCA2 but not UCA1, bound to eOD-GT8 with affinities lower to several recently described putative VRC01-class bnAb precursors (Havenar-Daughton et al., 2018).

We next determined whether the PCIN63-UCA variants could bind to autologous envelope sequences isolated from the PC063 donor. Autologous envelopes were sequenced by NGS and single genome amplification (SGA) at each time point before 40 mpi (Figure S4A). As described for other donors, PC063 Env variants became increasingly resistant to PCIN63 mAbs as mutations accumulated in loop D, CD4bs loop, and V5 loops, resulting in complete escape at month-61 (Figure S4B). Notably, among the set of sequences there was one rare virus variant isolated at 28 months post infection (M28cH1), lacking both the N276- and N462-glycans. As described previously, these glycans surround the CD4bs and are thought to obstruct binding of VRC01-class antibodies. However, none of the putative PCIN63-UCA Abs neutralized any of the autologous Env clones tested (Figure S4C) and no binding could be detected to the corresponding gp120 proteins captured from lysed pseudovirions or to recombinant gp120 from representative Env clones (Figure S4C), although, Env variants from 28 and 33 mpi had poor expression and therefore could not be evaluated. Overall, despite the availability of longitudinal sampling, UCA determination for this lineage remains partially ambiguous and the Env variants that triggered the PCIN63 Ab lineage is yet to be confirmed.

Autologous Adaptation to the N276-Glycan Is Associated with Heterologous Neutralization

Accommodation of the N276 glycan near the CD4bs is arguably the primary obstacle in eliciting VRC01-class responses. To determine at which point this occurred in the PC63 lineage, we next systematically evaluated the functional contributions of each SHM motif to neutralization breadth and potency. We generated PCIN63-UCA/71I chimeric antibody variants containing or lacking individual SHM motifs and evaluated each variant for neutralization against N276-glycan bearing or lacking autologous viruses as well as against a global heterologous 12-virus panel (deCamp et al., 2014) (Figure 4).

(B) Delineation of PCIN63 Ab heavy (HC) and light (KC) chains V-segments SHM motifs (gray) on amino-acid sequences of PCIN63-71I and compared to mature VRC01, minVRC01, 12A21, and min12A21 and respective germlines. Residues varying from VH1-2 and VL putative germlines are indicated by a single letter code.
(C and D) Longitudinal frequency of PCIN63 Ab lineage NGS sequences containing 1–5 residues mutated from germline motif to any of the amino-acid found in the isolated mature PCIN63 Abs. (C) Left panel shows motifs containing the same number of SHMs but varying in amino-acid sequence are separated by white lines within each shade of the motif-coded color. Motifs are arranged from top to bottom in chronological order of selection, i.e., time first mutation is detected for this motif. Right panel shows logogram representing the distribution of amino-acids at each position of the motif for the isolated mature PCIN63 Abs, the entire PCIN63 lineage NGS sequences and random Ab sequences from the same NGS dataset. Germline residues are indicated in black while mutated amino-acids found in the isolated matured PCIN63 Abs are colored using the pattern defined in (B).
(D) Motifs are grouped based on the time for all motif residues to be mutated (Thick line).
(E) Detail of LCDR1-Motif#4 evolution showing the dynamics for the six dominant versions (see also Figure S3E).

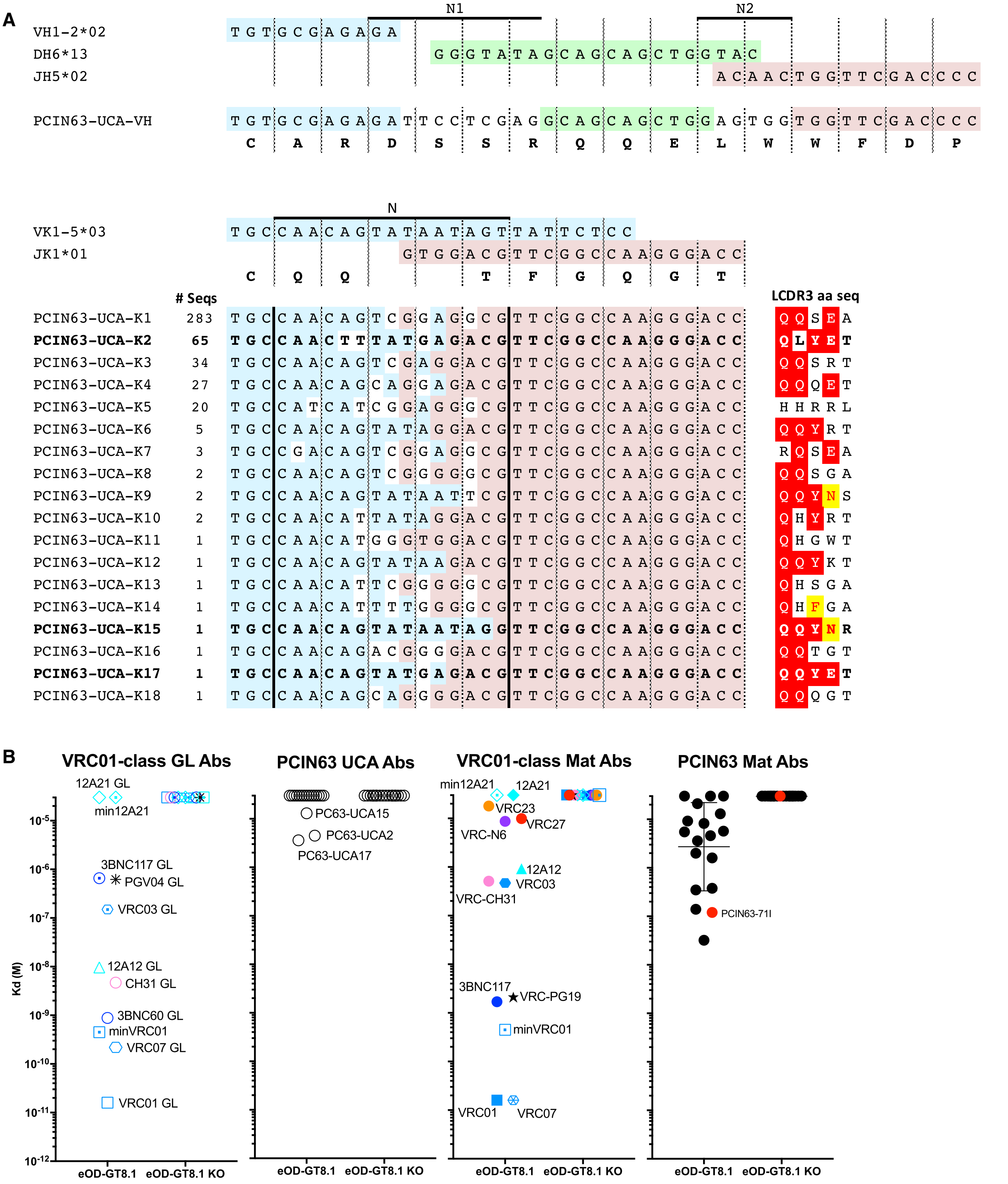

Figure 3. The Majority of Putative PCIN63 UCAs Do Not Bind eOD-GT8

(A) Identification of PCIN63 lineage precursor sequences. The CDR3 of putative PCIN63-UCA NGS sequences (100% identity to PCIN63 Abs HC and LC germline V+J genes) are aligned with IMGT V_H1-2*02, D_H6*13, J_H5*03, Vκ1-5*03, and Jκ1*01 germline genes sequences. The N regions of the junctions are indicated. For

(legend continued on next page)

Pairing the UCA-HC variants with the mature 71I-KC resulted in cross-neutralization of autologous viruses, particularly when HCDR2-motif#2 was introduced to the UCA-HC (Figure 4A, right). The combined LC SHMs alone allowed weaker neutralization of autologous Env lacking the N276-glycan than N276-bearing clones except when paired with the HCDR2-motif#2. In contrast, the UCA-KC2 variants paired with the mature 71I-HC predominantly neutralized viruses lacking the N276 glycan. These observations not only confirm the critical role of LC mutations in accommodating the N276-glycan but also suggest direct dependence on this glycan for binding of early PCIN63 intermediates to autologous Envs. Overall, pairing the UCA/71I-HC variants with the UCA-KC2 yielded broader autologous neutralization than pairing with UCA-KC1 (Figures 4, S5). This observation not only support UCA2 as the natural precursor of the PCIN63 lineage but also confirms an important role for the LCDR3 in elicitation of VRC01-class lineages.

Pairing the UCA-KC2 variants with an intermediate HC containing only HCDR2-motif#2 and HFWR3-motif#3 highlighted the co-dependence of LCDR1-motif4 and LFWR3-motif#6 for neutralization of autologous viruses with PNGS at position N276, with a critical role for $D32_{LC}$ in LCDR1-motif4 (Figure S4B, middle). The detection of autologous M28cH1 neutralization by almost all Ab variants containing at least one SHM motif suggests it may indeed have been the eliciting variant.

The absence of individual SHM motifs in either the HC or the KC had minimal effects on autologous neutralization when the variants were paired with affinity-mature KC or HC, respectively, indicating redundancy across the SHM motifs for epitope recognition. Although heterologous neutralization was overall correlated with autologous neutralization of N276-glycan bearing viruses, the absence of individual SHM motifs had greater impact on heterologous neutralization, which was most significantly reduced in absence of LCDR1-motif#4. In summary, SHM in HCDR2-motif#2, LCDR1-motif#4 and LFWR3-motif#6 are sufficient for autologous neutralization of variant bearing the N276-glycan by PCIN63 Abs.

PCIN63 bnAbs Engage the N276-Glycan of Some Env Strains

To better understand how the PCIN63 antibody lineage might interact with the N276 and other surrounding glycans, we next tested neutralization against heterologous viruses and their corresponding N276A mutants. The data revealed a hierarchy in the importance of glycans for neutralization by PCIN63 Abs, with $N276 \geq N197 > N262 \approx N301 > N462/N463 > N448$ (Figure S6). This trend is generally consistent with the effects observed for other VRC01-class bnAbs, particularly 12A21 and other Abs bearing a glycine-rich LCDR1 loop (VRC23, VRC27, VRC-CH31) with the exception of VRC-N6 (Figure S6, Table S5). We also observed significant variation between viral strains in their sensitivity to PCIN63 neutralization, as well as between PCIN63 mAbs in their ability to neutralize a given viral strain (Figure 5A). These variations were still apparent, although to a lesser extent, when pseudoviruses were produced in GnT $I^{-/-}$ cells (293-S) yielding predominantly Man5 glycans on HIV Env (Figure 5A) (Doores and Burton, 2010). The negative effect of glycan removal on neutralization for some viral strains by PCIN63 Abs was greater for N276 than other glycans and suggests that the Abs may directly depend on the N276-glycan for binding or neutralization in the context of some Env strains such as JR-CSF (Figure 5A). We evaluated other components of the epitope including surrounding glycans as well as key contact residues on Env and observed a general trend toward dependence on the N276-glycan when other obstructive features are present (JR-CSF) but accommodation of the N276 glycan when surrounding obstructive features are missing (JR-FL), such as the N461-glycan and the lack of N197- and N234-glycans (Figure 5A). We next determined whether there were shared motifs that lead to dependence or accommodation of the N276 glycan. An alignment of the PCIN63 antibody sequences that are or are not dependent on the N276-glycan revealed that the subset of PCIN63 antibodies that accommodate the N276 glycan share similar sequence features as the 12A21 antibody (Figure 5B). The N276-glycan dependent PCIN63 Abs carried SHM motifs possibly sub-optimal for high-affinity contacts with the CD4bs loop (non-aromatic residue $N54_{HC}$) and V5 loop ($Y61_{HC}$), while N276-glycan accommodating PCIN63 mAbs displayed a fourth Gly residues in LCDR1-motif#4 ($G34_{LC}$) and a more canonical LCDR3-motif#7 with aromatic residues at position 91 and 97 optimal for loop D and V5 loop binding (Figure 5B). Additionally, all PCIN63 mAbs also carried $R/W19_{HC}$ and $E/D76_{HC}$ mutations possibly involved in N197-glycan interactions. Further corroborating the difference in antibody subsets, we also observed that PCIN63 Abs with N276-glycan dependent or accommodating phenotypes clustered in separate branches of the lineage phylogenetic trees suggesting divergent evolution in response to the N276-glycan for this lineage (Figure S3D). Overall, these observations suggest a greater dependence on direct contact with the N276-glycan when the CD4bs loop is less accessible and Env protein contacts sub-optimal.

DISCUSSION

The exceptional breadth of VRC01-class bnAbs and their commonly shared features have long made them favored targets for rational vaccine design (Wu et al., 2010; Zhou et al., 2015). Despite major progress in the isolation and characterization of VRC01-class antibodies and the design of immunogens to prime VRC01-class precursors by immunization, the field has yet to elicit VRC01-class antibodies by immunization (Jardine et al., 2013; McGuire et al., 2013; Dosenovic et al., 2015; Jardine et al., 2015; Briney et al., 2016b; McGuire et al., 2016; Sok et al., 2016; Tian et al., 2016). Among other challenges, antibodies of this class can be up to 40% mutated from germline suggesting that during infection, VRC01-class antibodies require

putative PCIN63-UCA-KCs, the number of sequences (# seq) found in the NGS dataset and the corresponding LCDR3 amino-acid sequences (right) are also indicated. Residues previously described as important for binding to the HIV Env CD4bs are colored in red.

(B) Binding affinity of PCIN63 mature and putative UCA Abs and VRC01-class inferred germline (VRC01, VRC03, VRC07, 12A12, 12A21, 3BNC60, 3BNC117, VRC-PG04, VRC-CH31) and mature Abs (minVRC01, VRC01, VRC03, VRC07, VRC23, VRC27, 12A12, min12A21, 12A21, 3BNC117, VRC-PG19, VRC-CH31, VRC-N6) for the indicated previously described VH1-2 germline targeting immunogens, as measured by SPR. Data are representative of at least two experiments.

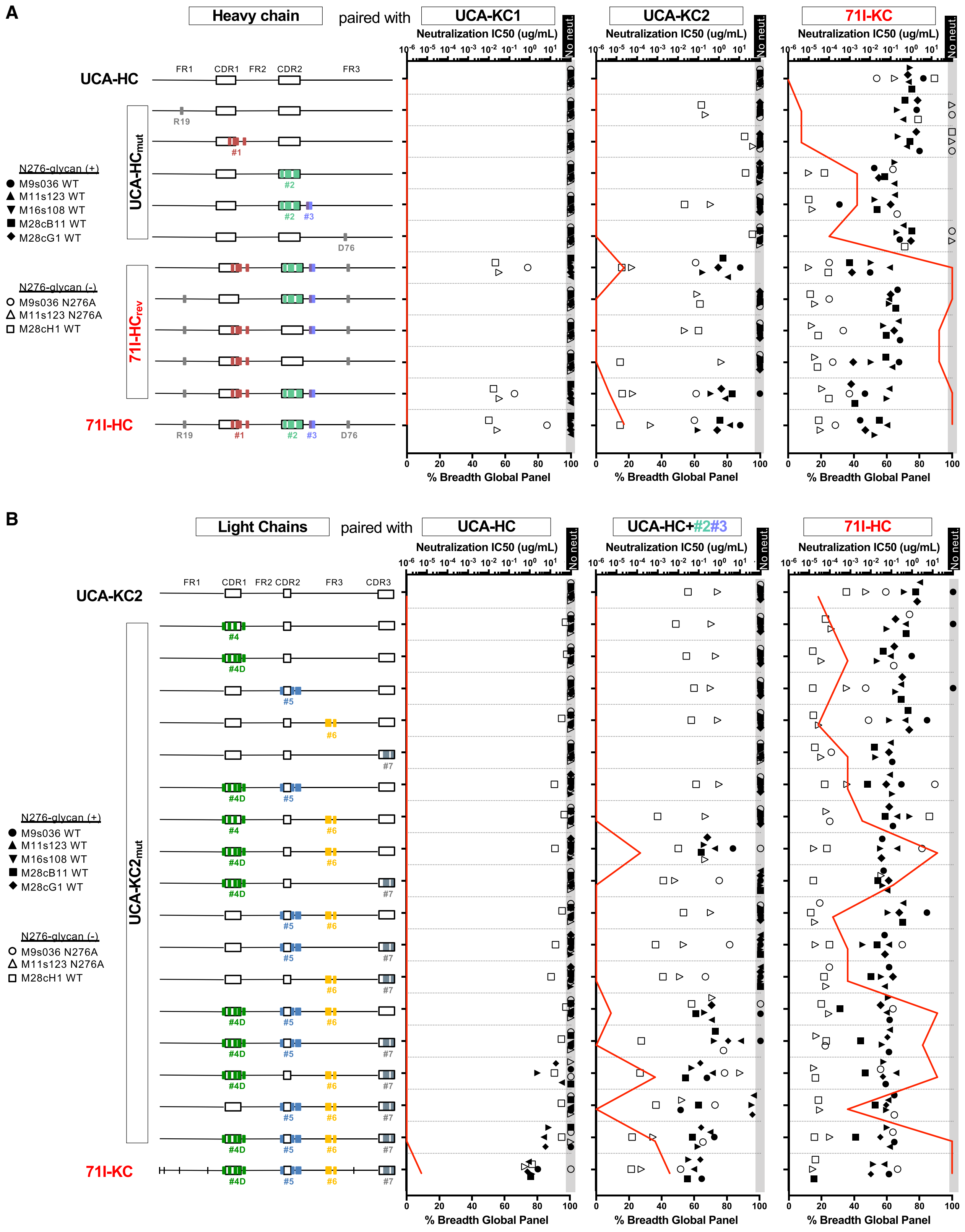

(legend on next page)

long periods of affinity maturation to target the occluded epitope that is surrounded by glycans on the HIV Env trimer (Huang et al., 2016; Sajadi et al., 2018). The glycan at N276 in particular has been previously described to be a key impediment for VRC01-class antibodies to target the CD4bs (Kong et al., 2016), and its removal has been shown to enhance the binding and neutralization activity of antibodies of this class. NGS analysis of VRC01 lineage ontogeny using longitudinal samples collected 5–20 years after infection indeed revealed a complex multi-branched lineage (Wu et al., 2015). However, late sampling only captured lineage sequences that were heavily mutated preventing the reconstruction of the early stages of VRC01 elicitation and maturation. Here, we describe the first complete longitudinal analysis of VRC01-class antibodies affinity maturation from elicitation to acquisition of neutralization breadth in natural infection. The findings also challenge the current strategies relying on glycan-deleted immunogens to select for Ab with glycan avoiding features and suggest that transient direct glycan-binding may help driving affinity maturation toward broad glycan accommodation.

Despite the number of VRC01-class mAbs isolated to date, the field has relied on reverted-germline Abs as templates for vaccine design, which were inferred either by phylogeny of B cell transcripts at a single time point or by reverting the V and J genes and approximating the HCDR3 of mature bnAbs (Jardine et al., 2013; McGuire et al., 2013; Jardine et al., 2015; Dosenovic et al., 2015; Briney et al., 2016b; McGuire et al., 2016; Bonsignori et al., 2018). Indeed, the uncertainty in determining the PCIN63 lineage precursor light chain despite the availability of contemporaneous samples reveal the importance of small LCDR3 variations on antibody binding, which highlights how sequence approximations could lead to different interpretations of germline antibody binding. We note in the case of PCIN63, both binding to eOD-GT8 and the weak neutralizing activity detected against the M28cH1 glycan-lacking variants by UCA2 chimeric Abs compared to UCA1 suggest but do not formerly validate UCA2 as the true PCIN63 precursor.

Previous studies reported that VRC01-class bnAbs require more time (>5 years) to develop during infection than bnAbs targeting other Env epitopes (1–3 years) (Landais et al., 2016; Lynch et al., 2012), a hypothesis supported by the high frequency of SHMs typically found among VRC01-class antibodies (Figure 1). While there was indeed a late onset for the PCIN63 antibody lineage, neutralization breadth for this lineage was acquired in less than 2 years and isolated mAbs from these time points showed only 10%–15% mutation from germline, which is 2- to 3-fold lower than typical VRC01-class antibodies. PCIN63 is the second VK1-5+ VRC01-subclass bnAb lineage isolated to date (Sajadi et al., 2018). Together with the recent finding that the VK1-5+ subclass of potential VRC01-class precursors were nearly as common as the most frequent VK3-20+ eOD-GT8-specific B cells in naive donor repertoires (Havenar-Daughton et al., 2018), this suggest (1) that the naive B cells isolated using germline-targeting immunogens may indeed represent true bnAbs precursors and (2) that this particular subclass of bnAbs may serve as a favorable target for vaccine design. Furthermore, evaluation of factors that might have favored the elicitation and focused affinity maturation of the PCIN63 lineage in donor PC063, i.e., frequencies of VRC01-class precursors and biased AID hotspots (see Methods) favoring affinity maturation at key contact residues, showed no significant differences compared to other donors. This suggests that similar focused maturation of PCIN63-like antibodies, with more classical features compared to other bnAbs of this class, might be readily achievable by vaccination.

To inform VRC01-class antibodies targeting vaccine strategies, we thus phylogenetically retraced the evolution of key SHM motifs and functionally tested their contribution to autologous and heterologous neutralization. The observation that PCIN63 lineage heavy chain sampled a greater sequence diversity compared to the light chain is consistent with a stringent selection pressure on the light chain to accommodate glycans surrounding the CD4bs followed by honing of the antibody-epitope interactions through gradual selection of affinity mutations in the heavy chain. Previous studies demonstrated that VRC01-class bnAbs SHM in the LC specifically were critical for the adaptation to the N276- and V5-loop glycans and are required for neutralization breadth. Accordingly, most immunization strategies are designed to prime VRC01-class precursors with immunogens lacking these glycans (eOD-GT8, 426c and donor NIH-45 Env derived molecules). In these studies, the Abs elicited by glycan-lacking priming immunogens typically cannot bind or neutralize Env constructs with the N276-glycan present. The responses are then typically boosted with more native-like Env proteins incrementally incorporating glycans to select for HC and LC mutations that enable binding in the presence of the N276 glycan. However, this strategy has only shown success in models where precursor frequencies are high or when the animal model contains an affinity-mature VRC01-class heavy- or light-chain gene (Briney et al., 2016b; Dosenovic et al., 2015; Jardine et al., 2015; McGuire et al., 2016; Sok et al., 2016; Tian et al., 2016).

Here we showed that UCA-HC/mature-KC chimeric Ab displayed weak neutralizing activity of the N276-glycan bearing autologous clones but not of their N276-glycan KO counterpart suggesting that direct engagement of the N276-glycan may

Figure 4. SHM Motifs in HCDR2, LCDR1, and LFWR3 Are Sufficient for Autologous Neutralization of N276-glycan Bearing Viral Variants

Chimeric Abs were tested for neutralization of the indicated WT and N276A autologous Env clones sensitive to neutralization by PCIN63-71I and against the global 12-virus panel (deCamp et al., 2014). Autologous neutralization IC_{50} are plotted with symbols according to the legend, and neutralization breadth (percentage of viruses neutralized) on the global panel is indicated with a red line.

(A) PCIN63-71I HC were either introduced into the PCIN63-UCA-HC (mut) or reverted to germline (rev). SHM motifs are color-coded as in Figure 2B and their position is indicated with diagrams. Mutated constructs were paired with WT PCIN63-UCA-KC1 (left), PCIN63-UCA-KC2 (middle), or PCIN63-71I (right) LC and tested for neutralization.

(B) PCIN63-71I-KC SHM motifs (as defined in Figure 2B) were introduced into the PCIN63-UCA-KC2, individually or in combination. Motifs are color-coded and their sequence position is indicated with a diagram. Mutated constructs were paired with WT PCIN63-UCA (left), PCIN63-UCA+Motif#2+Motif#3 (middle) or PCIN63-71I (right) HC. See also Figure S5. Data are representative of at least two experiments.

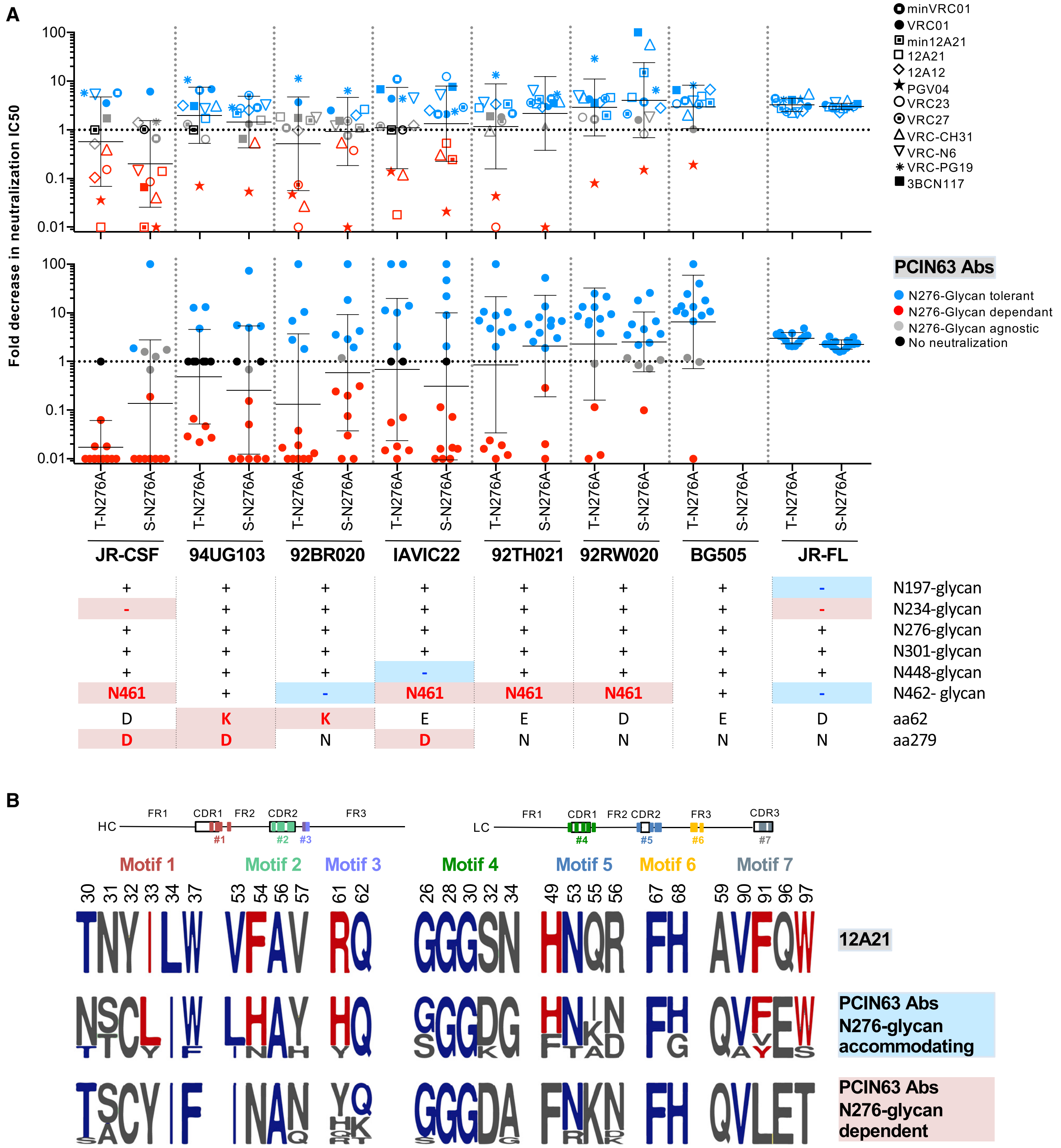

Figure 5. Neutralization of PCIN63 and Other VRC01-Class Antibodies Is Strain-Specifically N276-Glycan Dependent

(A) Fold decrease in neutralization IC_{50} of PCIN63 Abs (lower panel) and the listed VRC01-class Abs (upper panel) for N276A mutant of the indicated pseudotyped Env strains produced in 293-T or 293-S ($Gtnl^{-/-}$) cells compared to WT. The geomean with SD are shown. Relevant features of the selected Env strains are indicated above. Symbols are color-coded according to the permissive (blue) versus obstructive (red) potential regarding access to the CD4bs. See also Figure S6. Data are representative of at least two experiments.

(B) Logogram of the PCIN63 SHM motifs amino acid sequences for PCIN63 Abs segregated according to their sensitivity to N276-glycan removal (A) and compared to corresponding sequences of 12A12, 12A21, and min12A21 Abs. Residues common to 12A21 and PCIN63 N276-glycan accommodating Abs are colored in red. Residues shared between 12A21 and all PCIN63 Abs are colored in blue. SHM motifs are color-coded as in Figure 2 and their position is indicated with diagrams. See also Figures S2, S6, Table S5.

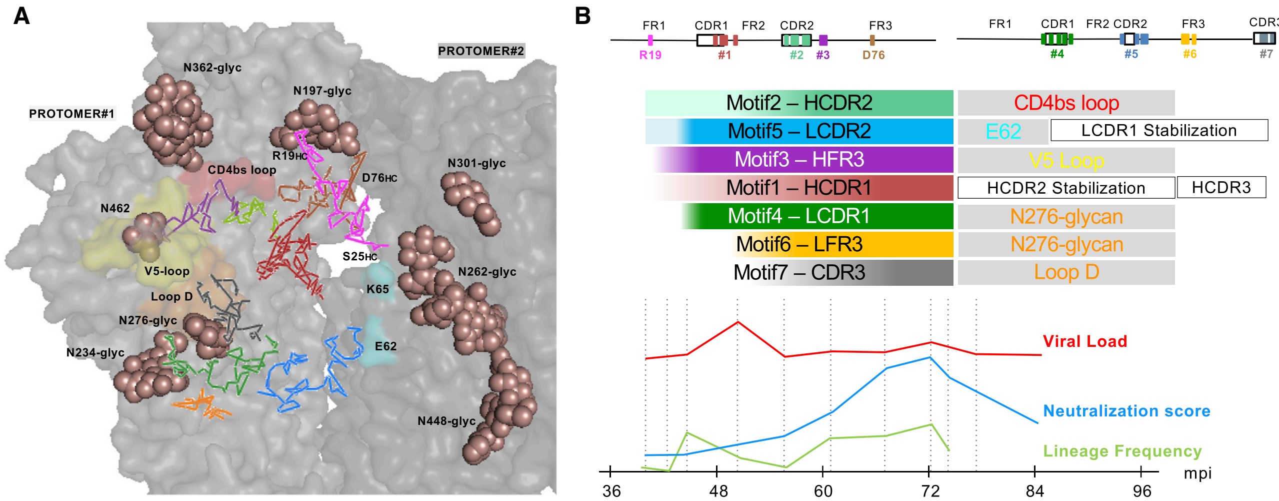

Figure 6. Modeling of PCIN63 bnAb Env Contacts Support a Possible Role of Glycan Interactions during Maturation and Lineage CD4bs Immunofocusing

(A) *In silico* modeling of 12A21 bound to BG505 SOSIP.664 using the published structures of 12A21 bound to gp120 (PDB ID: 4JPW) (Klein et al., 2013) and glycosylated BG505 SOSIP.664 bound to 35O22 and PGT122 (PDB ID: 6DE7) (Zhang et al., 2018). Two of the three Env protomers are displayed in shades of gray as transparent surfaces highlighting the CD4bs loop (red), loop D (orange), the V5 loop (yellow) on one protomer and other putative contact residues (cyan) on the second protomer. Glycans surrounding the CD4bs protruding from both protomers are shown as brown spheres and labeled. 12A21 HC and LC residues corresponding to PCIN63 SHM motifs are shown as ribbon and color-coded as defined in Figure 2B and represented in a diagram (B). Additional motifs in HFR1 (aa19-25 – pink) and HFWR3 (aa71-76 – brown) are also highlighted.

(B) PC63 infection timeline summary showing the evolution of viral load (red), serum neutralization breadth-potency (score, blue), and overall PCIN63 Ab lineage frequency in the periphery (green line). PCIN63 Ab lineage maturation is further detailed above for each SHM motif as shaded bars from unmutated (white) to fully mature (full motif-specific color) according to the kinetic analysis detailed in Figure 2. Putative functional impact of SHM motifs maturation regarding contact with Env (gray filled boxes) or internal structural stabilization (open boxes) inferred from the model in (A) are shown color-coded accordingly.

have facilitated binding. The lineage then affinity matures into two main branches, where one remains dependent on the N276 glycan for binding and the other gaining affinity for the CD4bs epitope in the absence of the N276 glycan. Together with the recent finding that VRC01 inferred GL Ab interacted with the N276-glycan of 426c gp120 core lacking the V1-V2-V3 loops (Borst et al., 2018), these data suggest an alternative vaccine design strategy where the N276-glycan is present on the germline-targeting immunogens at the initial priming stages to select for early intermediates with glycan-binding properties, which could be subsequently boosted with immunogens to affinity mature intermediate precursors to relinquish dependency on the glycan and acquire higher affinity for the CD4bs epitope. A similar strategy was suggested for V2-apex directed Abs upon observation that varying affinities for particular glycoforms were associated with the elicitation of the PCT64 lineage (Landais et al., 2017, Rantalainen et al., 2018).

To better understand the putative interactions between PCIN63 Abs and the Env trimer, we constructed an *in silico* model of 12A21 bound to BG505 SOSIP.664 using independently published structures (Klein et al., 2013; Zhang et al., 2018) (Figure 6). This model suggests contacts between E62 and CDR2-N57$_{HC}$, and N279 and CDR3-E96$_{LC}$ consistent with the strain-specific N276-glycan dependent variations observed in Figure 5A. Together with the evidence of early N276-glycan binding and significant toggling of the HCDR2-motif#2, this model suggests that PCIN63 Abs maturation pathway required a fine balance between glycan avoidance to better access the CD4bs loop and Loop D contact residues, and direct glycan binding to counter lower affinity of early viral intermediates evolving to escape autologous Ab responses. While acquiring a deletion in LCDR1 to avoid the N276-glycan, as seen in most VRC01-class Abs, might be more difficult to reproduce by vaccination, our data suggest that immunization strategies should not focus on glycan-deleted immunogens alone to drive affinity maturation of this lineage.

The trajectory for affinity maturation to broadly neutralizing antibodies in natural infection follows complex pathways with many "dead-ends" (Bhiman et al., 2015; Sok et al., 2014) that may be avoided with more focused and targeted rational vaccine design strategies. The isolation of the PCIN63 bnAbs and the identification of their UCA will enable the development of a germline mouse model for a CD4bs lineage known to develop broadly neutralizing activity. Finally, the identification of antibody intermediates and viral sequences will enable the evaluation of candidate boosts to refine immunization schemes that might drive such responses by vaccination.

STAR★METHODS

Detailed methods are provided in the online version of this paper and include the following:

- KEY RESOURCES TABLE
- CONTACT FOR REAGENT AND RESOURCES SHARING
- EXPERIMENTAL MODEL AND SUBJECT DETAILS
  - Human samples
  - Cell Lines

- METHODS DETAILS
    - Single memory B cell sorting and isolation of PCIN63 monoclonal antibodies
    - PCIN63 antibody expression and purification
    - B cell repertoire next generation sequencing and computational analysis
    - AID hotspots analysis
    - Single genome amplification (SGA), sequencing and cloning
    - Full-length *env* amplification sequencing and computational analysis
    - Neutralization assay
    - Serum adsorptions
    - Surface plasmon resonance (SPR)
    - ELISA assays
- QUANTIFICATION AND STATISTICAL ANALYSIS
- DATA AND CODE AVAILABILITY

SUPPLEMENTAL INFORMATION

Supplemental Information can be found online at https://doi.org/10.1016/j.immuni.2019.06.004.

CONSORTIA

The IAVI Protocol C Investigators Susan Allen (Emory University, USA and ZERHP, Zambia), William Kilembe (ZEHRP, Zambia), Shabir Lakhi (ZEHRP, Zambia), Mubiana Inambao (ZEHRP, Zambia), Etienne Karita (Rwanda-Zambia HIV Research Group, Project San Francisco, Kigali, Rwanda), Anatoli Kamali (MRC/UVRI Uganda), Eduard J. Sanders (KEMRI, Kenya and Oxford University, UK), Omu Anzala (KAVI, Kenya), Vinodh Edward (The Aurum Institute, South Africa), Linda-Gail Bekker (Cape Town University, South Africa), Jianming Tang (University of Alabama Birmingham, USA). The IAVI African HIV Research Network also includes Jill Gilmour (IAVI and London Imperial College, UK), Eric Hunter (Emory University, USA), Matt Price (IAVI and University of California San Francisco, USA).

ACKNOWLEDGMENTS

IAVI's work is made possible by generous support from many donors including: the Bill & Melinda Gates Foundation, the Ministry of Foreign Affairs of Denmark, Irish Aid, the Ministry of Finance of Japan in partnership with The World Bank, the Ministry of Foreign Affairs of the Netherlands, the Norwegian Agency for Development Cooperation (NORAD), the United Kingdom Department for International Development (DFID), and the United States Agency for International Development (USAID). The full list of IAVI donors is available at www.iavi.org. This study was made possible by the generous support of the American people through USAID. The contents are the responsibility of the International AIDS Vaccine Initiative and do not necessarily reflect the views of USAID or the United States Government. This work was also supported by National Institute of Health (NIH) Center for HIV/AIDS Vaccine Immunology and Immunogen Discovery Grant UM1AI100663 to BB, DS and DRB, as well as the International AIDS Vaccine Initiative Neutralizing Antibody Consortium through the Collaboration for AIDS Vaccine Discovery grants OPP1084519 and OPP1115782. BM was supported by grants R00AI120851 and UM1AI068618 from the National Institute of Allergy and Infectious Diseases (NIAID). The content is solely the responsibility of the authors and does not necessarily represent the official views of NIH. P.L.M. is supported by the South African Research Chairs Initiative of the Department of Science and Technology and National Research Foundation of South Africa (Grant No 98341). The content is solely the responsibility of the authors and does not necessarily represent the official views of the National Institute of Allergy and Infectious Diseases, the National Institute of General Medical Sciences or the National Institutes of Health.

PacBio SMRT sequencing was performed with the support of the Translational Virology Core at the UC San Diego Center for AIDS Research (P30 AI036214), and the IGM Genomics Center, University of California, San Diego, La Jolla, California. We are very grateful to and thank all of the Protocol C participants and clinical investigators, as well as all of the Protocol C project team members for all of the support provided for this study, particularly Matt Price (IAVI and University of California San Francisco, USA), Brendan McAtarsney (London Imperial College, UK) and Jonathan Hare (IAVI and London Imperial College, UK) for clinical data management and coordinating the samples transfers and shipments for this study. Finally we would like to express our gratitude to Christina Corbaci (The Scripps Research Institute, CA, USA) for her expert assistance in the design of the graphical abstract.

AUTHORS CONTRIBUTIONS

Conceptualization: S. Menis, W.K., I.P.C.I. & I.A.H.R.N., P.P., B.M., P.L.M., B. Briney, D.S., and E.L. Methodology: S. Menis, B.M., P.L.M. and E.L. Software: C.J., S. Menis, B.M. and B. Briney. Validation: E.L. Formal Analysis: J.U., B. Bagaya, C.J., T.S., B.M., P.L.M., B. Briney, and E.L. Investigation: J.U., B. Bagaya, C.J., T.S., K.F.-S., T.B., S. Mohan, T.V., O.K., S. Madzorera, D.K., B.L., M.N., and E.L. Resources: W.K., I.P.C.I. & I.A.H.R.N., W.R.S., D.R.B., and E.L. Data Curation: B.M., B. Briney, and E.L. Writing – Original Draft: J.U., B. Briney, and E.L. Writing – Review & Editing: J.U., B. Bagaya, W.R.S., D.R.B., B.M., P.L.M., B. Briney, D.S., and E.L. Visualization: J.U., T.S., S. Menis, B.M., B. Briney, D.S., and E.L. Supervision: T.S., B.M., P.L.M., B. Briney, D.S., and E.L. Project Administration: E.L. Funding Acquisition: W.R.S., D.R.B., B.M., P.L.M., B. Briney, D.S., and E.L.

DECLARATION OF INTERESTS

Some authors have filed a patent application whose subject matter relates to this work.

Received: November 20, 2018
Revised: March 31, 2019
Accepted: June 6, 2019
Published: July 16, 2019

REFERENCES

Bhiman, J.N., Anthony, C., Doria-Rose, N.A., Karimanzira, O., Schramm, C.A., Khoza, T., Kitchin, D., Botha, G., Gorman, J., Garrett, N.J., et al. (2015). Viral variants that initiate and drive maturation of V1V2-directed HIV-1 broadly neutralizing antibodies. Nat. Med. *21*, 1332–1336.

Bonsignori, M., Zhou, T., Sheng, Z., Chen, L., Gao, F., Joyce, M.G., Ozorowski, G., Chuang, G.-Y., Schramm, C.A., Wiehe, K., et al.; NISC Comparative Sequencing Program (2016). Maturation Pathway from Germline to Broad HIV-1 Neutralizer of a CD4-Mimic Antibody. Cell *165*, 449–463.

Bonsignori, M., Kreider, E.F., Fera, D., Meyerhoff, R.R., Bradley, T., Wiehe, K., Alam, S.M., Aussedat, B., Walkowicz, W.E., Hwang, K.-K., Saunders, K.O., Zhang, R., Gladden, M.A., Monroe, A., Kumar, A., Xia, S.-M., Cooper, M., Louder, M.K., McKee, K., Bailer, R.T., Pier, B.W., Jette, C.A., Kelsoe, G., Williams, W.B., Morris, L., Kappes, J., Wagh, K., Kamanga, G., Cohen, M.S., Hraber, P.T., Montefiori, D.C., Trama, A., Liao, H.-X., Kepler, T.B., Moody, M.A., Gao, F., Danishefsky, S.J., Mascola, J.R., Shaw, G.M., Hahn, B.H., Harrison, S.C., Korber, B.T., and Haynes, B.F. (2017). Staged induction of HIV-1 glycan-dependent broadly neutralizing antibodies. Sci. Trans. Med. *9*, eaai7514.

Bonsignori, M., Scott, E., Wiehe, K., Easterhoff, D., Alam, S.M., Hwang, K.-K., Cooper, M., Xia, S.-M., Zhang, R., Montefiori, D.C., et al. (2018). Inference of the HIV-1 VRC01 Antibody Lineage Unmutated Common Ancestor Reveals Alternative Pathways to Overcome a Key Glycan Barrier. Immunity *49*, 1162–1174.e8.

Borst, A.J., Weidle, C.E., Gray, M.D., Frenz, B., Snijder, J., Joyce, M.G., Georgiev, I.S., Stewart-Jones, G.B., Kwong, P.D., McGuire, A.T., DiMaio, F., Stamatatos, L., Pancera, M., and Veesler, D. (2018). Germline VRC01 antibody recognition of a modified clade C HIV-1 envelope trimer and a glycosylated HIV-1 gp120 core. eLife *7*, 37688.

Briney, B., Le, K., Zhu, J., and Burton, D.R. (2016a). Clonify: unseeded antibody lineage assignment from next-generation sequencing data. Sci. Rep. *6*, 23901.

Briney, B., Sok, D., Jardine, J.G., Kulp, D.W., Skog, P., Menis, S., Jacak, R., Kalyuzhniy, O., de Val, N., Sesterhenn, F., et al. (2016b). Tailored Immunogens Direct Affinity Maturation toward HIV Neutralizing Antibodies. Cell *166*, 1459–1470.e11.

deCamp, A., Hraber, P., Bailer, R.T., Seaman, M.S., Ochsenbauer, C., Kappes, J., Gottardo, R., Edlefsen, P., Self, S., Tang, H., et al. (2014). Global panel of HIV-1 Env reference strains for standardized assessments of vaccine-elicited neutralizing antibodies. J. Virol. *88*, 2489–2507.

Doores, K.J., and Burton, D.R. (2010). Variable loop glycan dependency of the broad and potent HIV-1-neutralizing antibodies PG9 and PG16. J. Virol. *84*, 10510–10521.

Dosenovic, P., von Boehmer, L., Escolano, A., Jardine, J., Freund, N.T., Gitlin, A.D., McGuire, A.T., Kulp, D.W., Oliveira, T., Scharf, L., et al. (2015). Immunization for HIV-1 Broadly Neutralizing Antibodies in Human Ig Knockin Mice. Cell *161*, 1505–1515.

Edgar, R.C. (2010). Search and clustering orders of magnitude faster than BLAST. Bioinformatics *26*, 2460–2461.

Eren, K., Weaver, S., Ketteringham, R., Valentyn, M., Laird Smith, M., Kumar, V., Mohan, S., Kosakovsky Pond, S.L., and Murrell, B. (2018). Full-Length Envelope Analyzer (FLEA): A tool for longitudinal analysis of viral amplicons. PLoS Comput. Biol. *14*, e1006498.

Falkowska, E., Ramos, A., Feng, Y., Zhou, T., Moquin, S., Walker, L.M., Wu, X., Seaman, M.S., Wrin, T., Kwong, P.D., et al. (2012). PGV04, an HIV-1 gp120 CD4 binding site antibody, is broad and potent in neutralization but does not induce conformational changes characteristic of CD4. J. Virol. *86*, 4394–4403.

Fauci, A.S. (2017). An HIV Vaccine Is Essential for Ending the HIV/AIDS Pandemic. JAMA *318*, 1535–1536.

Gao, F., Robertson, D.L., Morrison, S.G., Hui, H., Craig, S., Decker, J., Fultz, P.N., Girard, M., Shaw, G.M., Hahn, B.H., and Sharp, P.M. (1996). The heterosexual human immunodeficiency virus type 1 epidemic in Thailand is caused by an intersubtype (A/E) recombinant of African origin. J. Virol. *70*, 7013–7029.

Havenar-Daughton, C., Sarkar, A., Kulp, D.W., Toy, L., Hu, X., Deresa, I., Kalyuzhniy, O., Kaushik, K., Upadhyay, A.A., Menis, S., Landais, E., Cao, L., Diedrich, J.K., Kumar, S., Schiffner, T., Reiss, S.M., Seumois, G., Yates, J.R., Paulson, J.C., Bosinger, S.E., Wilson, I.A., Schief, W.R., and Crotty, S. (2018). The human naive B cell repertoire contains distinct subclasses for a germline-targeting HIV-1 vaccine immunogen. Sci. Transl. Med. *10*, eaat0381.

Huang, J., Kang, B.H., Ishida, E., Zhou, T., Griesman, T., Sheng, Z., Wu, F., Doria-Rose, N.A., Zhang, B., McKee, K., et al. (2016). Identification of a CD4-Binding-Site Antibody to HIV that Evolved Near-Pan Neutralization Breadth. Immunity *45*, 1108–1121.

Huerta-Cepas, J., Dopazo, J., and Gabaldón, T. (2010). ETE: a python Environment for Tree Exploration. BMC Bioinformatics *11*, 24.

Jardine, J., Julien, J.-P., Menis, S., Ota, T., Kalyuzhniy, O., McGuire, A., Sok, D., Huang, P.-S., MacPherson, S., Jones, M., et al. (2013). Rational HIV immunogen design to target specific germline B cell receptors. Science *340*, 711–716.

Jardine, J.G., Ota, T., Sok, D., Pauthner, M., Kulp, D.W., Kalyuzhniy, O., Skog, P.D., Thinnes, T.C., Bhullar, D., Briney, B., et al. (2015). HIV-1 VACCINES. Priming a broadly neutralizing antibody response to HIV-1 using a germline-targeting immunogen. Science *349*, 156–161.

Jardine, J.G., Kulp, D.W., Havenar-Daughton, C., Sarkar, A., Briney, B., Sok, D., Sesterhenn, F., Ereño-Orbea, J., Kalyuzhniy, O., Deresa, I., et al. (2016a). HIV-1 broadly neutralizing antibody precursor B cells revealed by germline-targeting immunogen. Science *351*, 1458–1463.

Jardine, J.G., Sok, D., Julien, J.-P., Briney, B., Sarkar, A., Liang, C.-H., Scherer, E.A., Henry Dunand, C.J., Adachi, Y., Diwanji, D., et al. (2016b). Minimally Mutated HIV-1 Broadly Neutralizing Antibodies to Guide Reductionist Vaccine Design. PLoS Pathog. *12*, e1005815.

Katoh, K., Kuma, K., Toh, H., and Miyata, T. (2005). MAFFT version 5: improvement in accuracy of multiple sequence alignment. Nucleic Acids Res. *33*, 511–518.

Katoh, K., and Standley, D.M. (2013). MAFFT multiple sequence alignment software version 7: improvements in performance and usability. Mol. Biol. Evol. *30*, 772–780.

Klein, F., Diskin, R., Scheid, J.F., Gaebler, C., Mouquet, H., Georgiev, I.S., Pancera, M., Zhou, T., Incesu, R.-B., Fu, B.Z., et al. (2013). Somatic mutations of the immunoglobulin framework are generally required for broad and potent HIV-1 neutralization. Cell *153*, 126–138.

Kong, L., Ju, B., Chen, Y., He, L., Ren, L., Liu, J., Hong, K., Su, B., Wang, Z., Ozorowski, G., et al. (2016). Key gp120 Glycans Pose Roadblocks to the Rapid Development of VRC01-Class Antibodies in an HIV-1-Infected Chinese Donor. Immunity *44*, 939–950.

Kraus, M.H., Parrish, N.F., Shaw, K.S., Decker, J.M., Keele, B.F., Salazar-Gonzalez, J.F., Grayson, T., McPherson, D.T., Ping, L.-H., Anderson, J.A., et al. (2010). A rev1-vpu polymorphism unique to HIV-1 subtype A and C strains impairs envelope glycoprotein expression from rev-vpu-env cassettes and reduces virion infectivity in pseudotyping assays. Virology *397*, 346–357.

Laird Smith, M., Murrell, B., Eren, K., Ignacio, C., Landais, E., Weaver, S., Phung, P., Ludka, C., Hepler, L., Caballero, G., et al.; IAVI Protocol C Investigators & The IAVI African HIV Research Network (2016). Rapid sequencing of complete env genes from primary HIV-1 samples. Virus Evol. *2*, vew018.

Landais, E., Huang, X., Havenar-Daughton, C., Murrell, B., Price, M.A., Wickramasinghe, L., Ramos, A., Bian, C.B., Simek, M., Allen, S., et al. (2016). Broadly Neutralizing Antibody Responses in a Large Longitudinal Sub-Saharan HIV Primary Infection Cohort. PLoS Pathog. *12*, e1005369.

Landais, E., Murrell, B., Briney, B., Murrell, S., Rantalainen, K., Berndsen, Z.T., Ramos, A., Wickramasinghe, L., Smith, M.L., Eren, K., et al.; IAVI Protocol C Investigators; IAVI African HIV Research Network (2017). HIV Envelope Glycoform Heterogeneity and Localized Diversity Govern the Initiation and Maturation of a V2 Apex Broadly Neutralizing Antibody Lineage. Immunity *47*, 990–1003.e9.

Lefranc, M.-P., Giudicelli, V., Ginestoux, C., Jabado-Michaloud, J., Folch, G., Bellahcene, F., Wu, Y., Gemrot, E., Brochet, X., Lane, J., Regnier, L., Ehrenmann, F., Lefranc, G., and Duroux, P. (2009). IMGT®, the international ImMunoGeneTics information system®. Nucleic Acids Res. *37*, D1006–D1012.

Li, Y., Migueles, S.A., Welcher, B., Svehla, K., Phogat, A., Louder, M.K., Wu, X., Shaw, G.M., Connors, M., Wyatt, R.T., and Mascola, J.R. (2007). Broad HIV-1 neutralization mediated by CD4-binding site antibodies. Nat. Med. *13*, 1032–1034.

Li, Y., Svehla, K., Louder, M.K., Wycuff, D., Phogat, S., Tang, M., Migueles, S.A., Wu, X., Phogat, A., Shaw, G.M., et al. (2009). Analysis of neutralization specificities in polyclonal sera derived from human immunodeficiency virus type 1-infected individuals. J. Virol. *83*, 1045–1059.

Li, Y., O'Dell, S., Wilson, R., Wu, X., Schmidt, S.D., Hogerkorp, C.M., Louder, M.K., Longo, N.S., Poulsen, C., Guenaga, J., et al. (2012). HIV-1 neutralizing antibodies display dual recognition of the primary and coreceptor binding sites and preferential binding to fully cleaved envelope glycoproteins. J. Virol. *86*, 11231–11241.

Lynch, R.M., Tran, L., Louder, M.K., Schmidt, S.D., Cohen, M., Dersimonian, R., Euler, Z., Gray, E.S., Abdool Karim, S., Kirchherr, J., et al.; CHAVI 001 Clinical Team Members (2012). The development of CD4 binding site antibodies during HIV-1 infection. J. Virol. *86*, 7588–7595.

MacLeod, D.T., Choi, N.M., Briney, B., Garces, F., Ver, L.S., Landais, E., Murrell, B., Wrin, T., Kilembe, W., Liang, C.-H., et al.; IAVI Protocol C Investigators & The IAVI African HIV Research Network (2016). Early Antibody Lineage Diversification and Independent Limb Maturation Lead to Broad HIV-1 Neutralization Targeting the Env High-Mannose Patch. Immunity *44*, 1215–1226.

Masella, A.P., Bartram, A.K., Truszkowski, J.M., Brown, D.G., and Neufeld, J.D. (2012). PANDAseq: paired-end assembler for illumina sequences. BMC Bioinformatics *13*, 31.

McGuire, A.T., Hoot, S., Dreyer, A.M., Lippy, A., Stuart, A., Cohen, K.W., Jardine, J., Menis, S., Scheid, J.F., West, A.P., et al. (2013). Engineering HIV envelope protein to activate germline B cell receptors of broadly neutralizing anti-CD4 binding site antibodies. J. Exp. Med. *210*, 655–663.

McGuire, A.T., Gray, M.D., Dosenovic, P., Gitlin, A.D., Freund, N.T., Petersen, J., Correnti, C., Johnsen, W., Kegel, R., Stuart, A.B., et al. (2016). Specifically modified Env immunogens activate B-cell precursors of broadly neutralizing HIV-1 antibodies in transgenic mice. Nat. Commun. *7*, 10618.

Price, M.N., Dehal, P.S., and Arkin, A.P. (2010). FastTree 2–approximately maximum-likelihood trees for large alignments. PLoS ONE *5*, e9490.

Rantalainen, K., Berndsen, Z.T., Murrell, S., Cao, L., Omorodion, O., Torres, J.L., Wu, M., Umotoy, J., Copps, J., Poignard, P., et al. (2018). Co-evolution of HIV Envelope and Apex-Targeting Neutralizing Antibody Lineage Provides Benchmarks for Vaccine Design. Cell Rep. *23*, 3249–3261.

Rogozin, I.B., Pavlov, Y.I., Bebenek, K., Matsuda, T., and Kunkel, T.A. (2001). Somatic mutation hotspots correlate with DNA polymerase eta error spectrum. Nat. Immunol. *2*, 530–536.

Rusert, P., Kouyos, R.D., Kadelka, C., Ebner, H., Schanz, M., Huber, M., Braun, D.L., Hozé, N., Scherrer, A., Magnus, C., et al.; Swiss HIV Cohort Study (2016). Determinants of HIV-1 broadly neutralizing antibody induction. Nat. Med. *22*, 1260–1267.

Sajadi, M.M., Dashti, A., Rikhtegaran Tehrani, Z., Tolbert, W.D., Seaman, M.S., Ouyang, X., Gohain, N., Pazgier, M., Kim, D., Cavet, G., et al. (2018). Identification of Near-Pan-neutralizing Antibodies against HIV-1 by Deconvolution of Plasma Humoral Responses. Cell *173*, 1783–1795.e14.

Salazar-Gonzalez, J.F., Bailes, E., Pham, K.T., Salazar, M.G., Guffey, M.B., Keele, B.F., Derdeyn, C.A., Farmer, P., Hunter, E., Allen, S., et al. (2008). Deciphering human immunodeficiency virus type 1 transmission and early envelope diversification by single-genome amplification and sequencing. J. Virol. *82*, 3952–3970.

Scheid, J.F., Mouquet, H., Ueberheide, B., Diskin, R., Klein, F., Oliveira, T.Y.K., Pietzsch, J., Fenyo, D., Abadir, A., Velinzon, K., et al. (2011). Sequence and structural convergence of broad and potent HIV antibodies that mimic CD4 binding. Science *333*, 1633–1637.

Seaman, M.S., Janes, H., Hawkins, N., Grandpre, L.E., Devoy, C., Giri, A., Coffey, R.T., Harris, L., Wood, B., Daniels, M.G., et al. (2010). Tiered categorization of a diverse panel of HIV-1 Env pseudoviruses for assessment of neutralizing antibodies. J. Virol. *84*, 1439–1452.

Sok, D., van Gils, M.J., Pauthner, M., Julien, J.-P., Saye-Francisco, K.L., Hsueh, J., Briney, B., Lee, J.H., Le, K.M., Lee, P.S., et al. (2014). Recombinant HIV envelope trimer selects for quaternary-dependent antibodies targeting the trimer apex. Proc. Natl. Acad. Sci. USA *111*, 17624–17629.

Sok, D., Briney, B., Jardine, J.G., Kulp, D.W., Menis, S., Pauthner, M., Wood, A., Lee, E.C., Le, K.M., Jones, M., et al. (2016). Priming HIV-1 broadly neutralizing antibody precursors in human Ig loci transgenic mice. Science *353*, 1557–1560.

Tian, M., Cheng, C., Chen, X., Duan, H., Cheng, H.-L., Dao, M., Sheng, Z., Kimble, M., Wang, L., Lin, S., et al. (2016). Induction of HIV Neutralizing Antibody Lineages in Mice with Diverse Precursor Repertoires. Cell *166*, 1471–1484.e18.

Tiller, T., Meffre, E., Yurasov, S., Tsuiji, M., Nussenzweig, M.C., and Wardemann, H. (2008). Efficient generation of monoclonal antibodies from single human B cells by single cell RT-PCR and expression vector cloning. J. Immunol. Methods *329*, 112–124.

Walker, L.M., Simek, M.D., Priddy, F., Gach, J.S., Wagner, D., Zwick, M.B., Phogat, S.K., Poignard, P., and Burton, D.R. (2010). A limited number of antibody specificities mediate broad and potent serum neutralization in selected HIV-1 infected individuals. PLoS Pathog. *6*, e1001028.

Wu, X., Yang, Z.Y., Li, Y., Hogerkorp, C.M., Schief, W.R., Seaman, M.S., Zhou, T., Schmidt, S.D., Wu, L., Xu, L., et al. (2010). Rational design of envelope identifies broadly neutralizing human monoclonal antibodies to HIV-1. Science *329*, 856–861.

Wu, X., Zhang, Z., Schramm, C.A., Joyce, M.G., Kwon, Y.D., Zhou, T., Sheng, Z., Zhang, B., O'Dell, S., McKee, K., et al.; NISC Comparative Sequencing Program (2015). Maturation and Diversity of the VRC01-Antibody Lineage over 15 Years of Chronic HIV-1 Infection. Cell *161*, 470–485.

Zhang, P., Gorman, J., Geng, H., Liu, Q., Lin, Y., Tsybovsky, Y., Go, E.P., Dey, B., Andine, T., Kwon, A., et al. (2018). Interdomain Stabilization Impairs CD4 Binding and Improves Immunogenicity of the HIV-1 Envelope Trimer. Cell Host Microbe *23*, 832–844.e6.

Zhou, T., Lynch, R.M., Chen, L., Acharya, P., Wu, X., Doria-Rose, N.A., Joyce, M.G., Lingwood, D., Soto, C., Bailer, R.T., et al.; NISC Comparative Sequencing Program (2015). Structural Repertoire of HIV-1-Neutralizing Antibodies Targeting the CD4 Supersite in 14 Donors. Cell *161*, 1280–1292.

STAR★METHODS

KEY RESOURCES TABLE

REAGENT or RESOURCE	SOURCE	IDENTIFIER
Antibodies		
Monoclonal anti-HIV-1 Env VRC01	NIH AIDS Reagent Program; www.hiv.lanl.gov	Cat# 12033; RRID: AB_2491019
Monoclonal anti-HIV-1 Env minVRC01	William Schief, Scripps schief@scripps.edu	N/A
Monoclonal anti-HIV-1 Env 12A12	Michel Nussenzweig The Rockefeller University nussen@rockefeller.edu	RRID: AB_2491040
Monoclonal anti-HIV-1 Env 12A21	Michel Nussenzweig The Rockefeller University nussen@rockefeller.edu	RRID: AB_2491036
Monoclonal anti-HIV-1 Env min12A21	William Schief, Scripps schief@scripps.edu	N/A
Monoclonal anti-HIV-1 Env VRC03	NIH AIDS Reagent Program; www.hiv.lanl.gov	Cat# 12032: RRID: AB_2491021
Monoclonal anti-HIV-1 Env VRC07	John R. Mascola, NIH; www.hiv.lanl.gov	N/A
Monoclonal anti-HIV-1 Env VRC23	John R. Mascola, NIH; www.hiv.lanl.gov	RRID: AB_2491072
Monoclonal anti-HIV-1 Env VRC27	John R. Mascola, NIH; www.hiv.lanl.gov	N/A
Monoclonal anti-HIV-1 Env VRC-CH31	NIH AIDS Reagent Program; www.hiv.lanl.gov	Cat# 12565; RRID: AB_2491024
Monoclonal anti-HIV-1 Env VRC-PG04	Dennis R. Burton, Scripps; www.hiv.lanl.gov	RRID: AB_2491022
Monoclonal anti-HIV-1 Env VRC-PG19	John R. Mascola, NIH; www.hiv.lanl.gov	N/A
Monoclonal anti-HIV-1 Env VRC-N6	NIH AIDS Reagent Program; www.hiv.lanl.gov	Cat# 12968
Monoclonal anti-HIV-1 Env 3BNC117	NIH AIDS Reagent Program; www.hiv.lanl.gov	Cat# 12474; RRID: AB_2491033
Monoclonal anti-HIV-1 Env PCIN63-UCAs, _66B to _77D	Elise Landais, IAVI (This Paper)	N/A
Monoclonal inferred germline Ab for anti-HIV-1 Env VRC01, VRC03, VRC07, 12A12, 12A21, 3BNC60, 3BNC117, VRC-PG04, VRC-CH31	William Schief, Scripps schief@scripps.edu	N/A
Polyclonal anti-HV-1 Env sheep purified Ab D7324	Aalto Bioreagents	Cat #D7324
Alkaline Phosphatase AffiniPure Goat Anti-Human IgG, F(ab')$_2$ fragment specific	Jackson ImmunoResearch	Cat# 109-055-097
Bacterial and Virus Strains		
37 HIV-1 Env-pseudotyped viruses	Elise Landais IAVI-NAC (elandais@iavi.org)	(Landais et al., 2016)
120 HIV-1 Env-pseudotyped viruses	D.Montefiori, Duke University (david.montefiori@duke.edu)	(Seaman et al., 2010)
Biological Samples		
PBMC from IAVI Protocol C Donor 63	Protocol C, IAVI	N/A
Serum from IAVI Protocol C Donor 63	Protocol C, IAVI	N/A
Chemicals, Peptides, and Recombinant Proteins		
100mM dNTP set	Thermo Fisher	Cat# 10297117
293Fectin	Thermo Fisher	Cat# 12347500
AMPure PB Beads	Pacific Biosciences	Cat# 100-265-900
Complete EDTA free protease inhibitors	Roche	Cat# 05056489001
**Dynabeads™ MyOne™ Tosylactivated**	Thermo Fisher	Cat# 65502
FUGENE 6	Promega	Cat# E2692
Galanthus nivalis lectin (snow drop), agarose bound	Vector Labs	Cat# AL-1243
GeneArt® Seamless Cloning and Assembly Enzyme Mix	Thermo Fisher	Cat# A14606
IgG Elution Buffer		

(*Continued on next page*)

Continued

REAGENT or RESOURCE	SOURCE	IDENTIFIER
Luciferase Cell Culture Lysis 5X Reagent	Promega	Cat# E1531
Phosphatase substrate	Sigma	Cat# S0942
Protein A Sepharose Fast Flow	GE healthcare	Cat# 17-1279-03
QC Lightning Mullti Site-Directed Mutagenesis Kit	Agilent Technologies	Cat# 210513
Qiaquick PCR purification kit	QIAGEN	Cat# 28106
Recombinant HIV-1-gp120 Antigen from 92BR020 Env strain	Elise Landais, IAVI-NAC (elandais@iavi.org)	Genebank: AY_669726.1
Recombinant HIV-1-gp120 Antigen from JR-CSF Env strain	Elise Landais, IAVI-NAC (elandais@iavi.org)	Genebank: AY_669718
Recombinant HIV-1-gp120 Antigen from PC63 Env clones	This paper	N/A
RNase OUT	Thermo Fisher	Cat# 10777019
SPRI Select Reagent	Bekman Coulter	Cat# B23317
SuperScript® III Reverse Transcriptase	Thermo Fisher	Cat# 18080-085
Critical Commercial Assays		
DH5a E.Coli	BioPioneer	Cat# GACC-96P
DNA 1200 Analysis Kit	Agilent Technologies	Cat# 5067-1508
HotStarTaq Plus DNA Polymerase Kit	QIAGEN	Cat# 203603
Luciferase 1000 Assay System	Promega	Cat# E4550
MiSeq Reagent Kit V3 (600-cycle)	Illumina	Cat# MS-102-3003
PacBio RS II C3 Sequencing Kit	Pacific Biosciences	Cat# P/N 100-254-800
QIAamp Viral RNA Mini Kit	QIAGEN	Cat# 52906
RNEasy Mini Purification Kit	QIAGEN	Cat# 74104
SuperScript® III First-Strand Synthesis System for RT-PCR	Thermo Fisher	Cat# 18080-051
Human Antibody Capture Kit	GE Healthcare Life Sciences	Cat# BR100839
4-12% Bis-Tris NuPAGE gel system	Thermo Fisher	Cat# NP0321BOX
SMRTbell Template Prep Kit 1.0	Pacific Biosciences	Cat# 100-259-100
Deposited Data		
PC63 Full-length Env longitudinal sequences	This Paper	GenBank: MK_749242 to MK_749296
PCIN63 bnAb lineage HC MiSeq sequences	This Paper	BioProject: PRJNA_545346
PCIN63-UCAs, _66B to _77D heavy chain nt sequences	This Paper	GenBank: MK_749197 to MK_749219
PCIN63-UCAs, _66B to _77D light chain nt sequences	This Paper	GenBank: MK_749220 to MK749241
Experimental Models: Cell Lines		
Human: HEK293T	ATCC	Cat# CRL-3216; RRID: CVCL_0063
Human: HEK293S GnT1-	ATCC	Cat# CRL-3022; RRID: CVCL_A785
Human: HeLa-derived TZM-bl	NIH AIDS Reagent Program	Cat# 8129-442; RRID: CVCL_B478
Human: FreeStyle 293F	Thermo Fisher	Cat# R79007; RRID: CVCL_D603
Oligonucleotides		
Env-F: GAGCAGAAGACAGTGGCAATGA	Integrated DNA Technologies	N/A
Env-R: CCACTTGCCACCCATBTTATAGCA	Integrated DNA Technologies	N/A
IgG NGS Primers	Integrated DNA Technologies	(Briney et al., 2016a)
Recombinant DNA		
Plasmid pcDNA3.1+	Thermo Fisher	Cat# V790-20
Plasmid pSG3Denv	NIH AIDS Reagent Program	Cat# 11051

(*Continued on next page*)

Continued

REAGENT or RESOURCE	SOURCE	IDENTIFIER
Software and Algorithms		
AbStar	Bryan Briney (briney@scripps.edu), The Scripps Research Instiute	https://github.com/briney/abstar
Clonify	Bryan Briney (briney@scripps.edu), The Scripps Research Instiute	https://github.com/briney/clonify-python
ETE Toolkit	Jaime Huerta-Cepa (jhuerta@crg.es), Centre for Genomic Regulation, Spain	http://ete.cgenomics.org
FastTree	Morgan N. Price (MorganNPrice@yahoo.com) Lawrence Berkely University	http://www.microbesonline.org/fasttree; RRID:SCR_015501
FigTree	Andrew Rambaut (andrew.rambaut@zoo.ox.ac.uk) University of Edinburgh	http://tree.bio.ed.ac.uk/software/figtree/; RRID:SCR_008515
Full-Length Env Analysis (FLEA) pipeline	Ben Murrell (murrellb@gmail.com), Karolinska Institutet, Sweden.	https://github.com/veg/flea-pipeline/
IMGT/V-QUEST	International ImMunoGeneTics Information System; Marie-Paule Lefranc (marie-paule.lefranc@igh.cnrs.fr), University of Montpellier, France	www.imgt.org; RRID: SCR_012780
MAFFT	Kazutaka Katoh (kkatoh@kuicr.kyoto-u.ac.jp) Kyoto University, Japan	http://www.biophys.kyoto-u.ac.jp/~katoh/programs/align/mafft; RRID: SCR_011811
Microsoft Excel	Microsoft Corp	RRID: SCR_016137
PacBio SMRTportal Version 2.3	Pacific Biosciences	http://www.pacb.com/products-and-services/analytical-software/smrt-analysis/; RRID: SCR_002942
PANDAseq	Josh D. Neufeld (ac.oolretawu@dlefuenj) University of Waterloo, Canada	https://github.com/neufeld/pandaseq; RRID: SCR_002705
PRISM6	GraphPad	http://www.graphpad.com/; RRID: SCR_002798
ProteOn Manager Software	Bio-Rad	Cat# 1760200
PyMol Molecular Graphics Systems Version 1.5.0.4	Schrodinger LLC	https://www.schrodinger.com/pymol; RRID: SCR_000305
USEARCH	Robert C. Edgar (robert@drive5.com)	http://www.drive5.com/usearch/
Other		
96S Super Magnet Plate	ALPAQUA	Cat# A001322
AKTA Pure 25M2	GE Healthcare Life Sciences	Cat# 29018228
C1000 Thermocycler	Bio-Rad	Cat# 1851196
FACS ARIA III	BD Biosciences	Cat# 744763
MiSeq Sequencer	Illumina	N/A
PacBio RS-II Sequencer	Pacific Biosciences	N/A
ProteOn XPR36 Protein Interaction Array System	Bio-Rad	Cat# 1760100
ProteOn GLC Sensor Chip	Bio-Rad	Cat# 1765011
Qubit 3.0 Fluorometer	Thermo Fisher	Cat# Q33216
Sanger Sequencing: bacterial colonies and plasmids	Genewiz	https://www.genewiz.com/
Superdex 200 HiLoad 16/600 column	GE Healthcare Life Sciences	Cat# 28989335
Synergy H1 Hybrid-Mode Microplate Reader	Biotek	N/A
VersaMax Microplate Reader	Molecular Devices	N/A

CONTACT FOR REAGENT AND RESOURCES SHARING

Further information and requests for resources and reagents should be directed to and will be fulfilled by the Lead Contact, Elise Landais (elandais@iavi.org).

EXPERIMENTAL MODEL AND SUBJECT DETAILS

Human samples

Donor PC063 was part of the IAVI sponsored Protocol C cohort in sub-Saharan Africa that involved rapid screening of 613 individuals with a recent history of HIV exposure for HIV antibodies in sub-Saharan Africa (Landais et al., 2016). Samples were collected with written, informed consent, and the study was reviewed and approved by institutional Ethics and Research Committees to the participating clinical investigators, namely Susan Allen (Emory University, GA, USA), William Kilembe (University of Zambia, Zambia), Shabir Lakhi (University of Zambia, Zambia), Mubiana Inambao (University of Zambia), Etienne Karita (Republic of Rwanda, Rwanda), Anatoli Kamali (MRC/UVRI Uganda), Eduard J. Sanders (KEMRI, Kenya), Omu Anzala (KAVI and University of Nairobi, Kenya), Vinodh Edward (University of Kwazulu Natal, South Africa), Linda-Gail Bekker (Cape Town University, South Africa), Jill Gilmour (London Imperial College, UK) and Eric Hunter (Emory University, GA, USA) as well as to Elise Landais (Scripps, CA, USA).

Donor PC063 is a female participant enrolled in the Protocol C longitudinal primary infection cohort at 34-year of age, approximately 7 weeks (2 months) after heterosexual infection by a subtype C HIV-1 virus and was identified as one of the top 20% neutralizers, neutralizing up to 85% of viruses on a 37-virus panel (Landais et al., 2016). The neutralizing activity was first detected in the plasma after 5 year of infection and steadily increased to reach a peak at 6 years, neutralizing 79% of viruses from a large panel (Seaman et al., 2010; Landais et al., 2016). The plasma broadly neutralizing activity was mapped to the CD4 binding site as it was competed by RSC3 and its absorption with rgp120 monomers was competed by b6 and sensitive to the D168R mutation (Landais et al., 2016).

Cell Lines

The female HEK-derived 293T, HEK293S N-acetylglucosaminyltransferase I-negative (GnTI−/−), and HeLa-derived TZM-bl cell lines were maintained in complete Dulbecco's Modified Eagle Medium (herein referred to as cDMEM) containing high- glucose Dulbecco's Modified Eagle Medium (DMEM, Thermo Fisher), 1X Penicillin-Streptomycin (Pen Strep, Thermo Fisher) and 10% fetal bovine serum (FBS, Gemini Bio Products) at 37°C and 5% CO_2. FreeStyle HEK-derived 293F cells (Thermo Fisher) were maintained in Freestyle 293 Expression Medium at 37°C and 10% CO_2 with shaking at 120 RPM.

METHODS DETAILS

Single memory B cell sorting and isolation of PCIN63 monoclonal antibodies

Sorting of antigen- and epitope-specific memory B cells was performed as previously described (MacLeod et al., 2016; Sok et al., 2014; Tiller et al., 2008; Wu et al., 2010).

Fluorescent-labeled antibodies recognizing cell surface markers were purchased from BD Biosciences. AVI-tagged WT and D368R YU2-gp140-Foldon proteins (plasmid generously provided by Y. Li) were produced, purified, labeled with biotin (Avidity), and coupled to streptavidin-PE, streptavidin-APC (Life Technologies), and streptavidin-BV421 (BD Biosciences), as previously described (Sok et al., 2014). Cells were stained with the Live/Dead Fixable Near-IR Dead Cell Stain Kit (Life Technologies) for 30 min on ice according to the manufacturer's instructions. Cells were then labeled with antibodies for surface markers together with probes for 1 hour in Brilliant Stain buffer (BD Biosciences) on ice. Cells were sorted into individual wells of a 96 well plate containing First Strand buffer containing DTT and RNaseOUT (Life Technologies) using a BD FACSAria III sorter and were immediately sealed and stored at-80°C after sorting each plate.

cDNA was generated from cells sorted into lysis buffer using Superscript III Reverse Transcriptase (Life Technologies) and random hexamers (Gene Link). Nested PCR amplification of heavy- and light-chain variable regions was performed using Multiplex PCR Kit (QIAGEN) and previously described primer sets (Tiller et al., 2008). Amplified heavy- and light-chain variable regions were sequenced and subsequently analyzed using IMGT (the International ImMunoGeneTics Information System, www.imgt.org) V-quest (Lefranc et al., 2009).

Antibodies of interest were cloned into expression vectors (Tiller et al., 2008) by re-amplification of sequences using the same primers but modified to introduce homology to the cut ends of the vector, and cloning was performed using the Seamless Cloning and Assembly Enzyme mix (Life Technologies) in expression vectors with the appropriate IgG1, Ig kappa or Ig lambda constant domain. Antibodies incorporating targeted amino acid mutations were generated by Quickchange mutagenesis (Stratagene).

PCIN63 antibody expression and purification

Antibodies HC and LC constructs were transiently expressed with the FreeStyle 293 Expression System (Invitrogen). Supernatant was collected after 4-5 days of culture and whole IgGs were purified with Protein A Sepharose (GE Healthcare). Purified proteins purity and integrity checked by SDS–PAGE.

B cell repertoire next generation sequencing and computational analysis

RNA was prepared (RNEasy kit, QIAGEN) from total PBMCs (a single of 10 million cells per time point) and was subjected to reverse transcription using barcoding primers that contain unique Ab identifiers as previously described (Briney et al., 2016a). The cDNA was

then amplified using a mix of gene specific primers containing unique identifiers. Illumina sequencing adapters and sample-specific indexes were added during a second round of PCR. Samples were quantified using fluorometry (Qubit; Life Technologies), pooled at approximately equimolar concentrations, and the sample pool was re-quantified before loading onto an Illumina MiSeq. Paired-end MiSeq reads were merged with PANDAseq (Masella et al., 2012). GL assignment, junction identification, and other basic Ab information was determined using AbStar (www.github.com/briney/abstar). Sequences were assigned to clonal lineages using Clonify (Briney et al., 2016a). PCIN63 lineage sequences were clustered at 97.5% identity with USEARCH (Edgar, 2010), and the size of each cluster was recorded. Cluster centroids were used to generate a multiple sequence alignment with MAFFT (Katoh et al., 2005), and a tree file was calculated with FastTree using default settings (Price et al., 2010). The phylogenetic tree was drawn in Python using the ETE Toolkit (Huerta-Cepas et al., 2010).

AID hotspots analysis

Frequency and distribution of AID hotspots in PCIN63 lineage HCs, including both mAb and NGS sequences as well as the identified UCA, variable gene region was compared to all IGHV1-2 heavy chain alleles in the IMGT database (http://www.imgt.org/genedb). Analysis was performed considering the entire Ab sequence, or each antibody region (CDR1, CDR2, FR1, FR2, FR3) separately. Individual mutations were considered to have occurred in an AID hotspot if the mutated nucleotide fell within a sequence region encoding an AID hotspot motif (RGYW, or the reverse complement WRCY (Rogozin et al., 2001) in the germline V gene sequence. Separately for each time point, the mean frequency of AID hotspots was determined by counting the number of AID hotspots in each sequence (considering both NGS and mAb sequences) and dividing by the total number of sequences obtained at the time point.

Single genome amplification (SGA), sequencing and cloning

HIV-1 RNA was isolated from plasma using the QIAGEN QIAamp Viral RNA kit, and reverse transcribed to cDNA using SuperScript III Reverse Transcriptase (Invitrogen, CA). The envelope genes were amplified from single genome templates (Salazar-Gonzalez et al., 2008) and amplicons were directly sequenced using the ABI PRISM Big Dye Terminator Cycle Sequencing Ready Reaction kit (Applied Biosystems, Foster City, CA) and resolved on an ABI 3100 automated genetic analyzer. The full-length env sequences were assembled and edited using Sequencher v.4.5 software (Genecodes, Ann Arbor, MI).

Selected envelope amplicons were cloned into the expression vector pcDNA 3.1 (directional) (Invitrogen) by re-amplification of SGA first-round products using Pfu Ultra II enzyme (Stratagene) with the EnvM primer, 59-TAGCCCTTCCAGT CCCCCCT TTTCTTTTA-39 (Gao et al., 1996) and directional primer, EnvAstop, 59-CAC CGGCTTAGGCATCTCCTATGGCAGGAAGAA-39 (Kraus et al., 2010). Cloned env genes were sequenced to confirm that they exactly matched the sequenced amplicon. Autologous clones were mutated at key residues within the C-strand using the Stratagene QuickChange II kit (Stratagene) as described by the manufacturer. Mutations were confirmed by sequencing. Envelope clones were used to generate single round of replication Env-pseudoviruses as described below.

Full-length *env* amplification sequencing and computational analysis

HIV-1 envelope genes were amplified and sequenced as described in Laird-Smith *et. al*. (2016) (Laird Smith et al., 2016). Briefly, virions were purified from 1-2 mL of plasma at each time point using a sucrose cushion and ultracentrifugation. Viral RNA was extracted (Viral RNA Mini Kit, QIAGEN) and subjected to RT-PCR (SuperScript III First Strand, Thermo Fisher). The cDNA was used as template to generate HIV-1 *env* amplicons, which were then purified (QIAquick, QIAGEN). Replicate PCR reactions for each sample were visualized, quantitated (2100 Bioanalyzer System, Agilent Biosciences) and pooled by sample. Preparation and sequencing of SMRTbell template libraries of approximately 2.6-kb insert size were performed according to the manufacturer's instructions (Pacific Biosciences).

CCS sequences were constructed using the PacBio SMRTportal software (version 2.3). The Full-Length Envelope Analysis (FLEA) pipeline (Eren et al., 2018) was used to error correct these CCS reads, and cluster them into near-identical clusters, inferring High Quality Consensus Sequences (HQCSs) for each cluster. Envelope phylogenies, as well as the dynamics of amino acid frequency evolution, were inferred from these HQCSs. MAFFT (v7.164b (Katoh and Standley, 2013), with manual curation, was used to create a multiple sequence alignment. Gappy regions were manually removed when reconstructing phylogenies, since their alignment is uncertain. Phylogenies were reconstructed with FastTree v2.1 (Price et al., 2010), and visualized with FigTree (http://tree.bio.ed.ac.uk/software/figtree/). Frequency kinetic plots and similar analyses were created with custom Mathematica scripts.

Selected full-length autologous *env* gene sequences were synthesized for representative clones of each time point using GeneArt® gene synthesis services (Life Technologies), then cloned into pcDNA3.1 vector (Life Technologies) for pseudovirus production. Mutagenesis was performed using Quickchange site-directed mutagenesis kit (Agilent Technologies).

Neutralization assay

Plasma and monoclonal antibodies neutralizing activity was assessed using single round of replication in TZM-bl target cells, as described previously (Landais et al., 2016) and in absence of DEAE-dextran. Briefly, wild-type (WT) and mutant pseudoviruses were generated by co-transfection of 293T cells with an Env-expressing plasmid and an Env-deficient genomic backbone plasmid

(pSG3ΔEnv). Pseudoviruses were harvested 72h post transfection for use in neutralization assays. Pseudoviruses incorporating single amino acid mutations were generated by Quickchange mutagenesis (Stratagene). Plasma samples were heat-inactivated at 56 C for 45min prior to use in neutralization assays.

Serum adsorptions

Serum adsorptions with antigen-coupled beads were performed using tosyl-activated magnetic beads (Life Technologies), as described previously (Li et al., 2009). Beads coupling was performed at a ratio of 1mg gp140 per 25mg of beads. Plasma samples were depleted of Abs binding to these proteins through multiple rounds of immunoprecipitation. The depletion of Abs of the desired specificity was confirmed by ELISA prior to using depleted serum in pseudovirus neutralization assays.

Surface plasmon resonance (SPR)

SPR experiments were performed on a Proteon XPR36 instrument (Bio-Rad) using GLC sensor chips (Bio-Rad) and 1x HBS buffer (Teknova) supplemented with 1 mg/mL BSA). Chips were prepared using the Human Antibody Capture Kit (GE Healthcare) according to manufacturer's instructions. For kinetic measurements, approximately 100 RUs of the indicated mAbs were captured onto the sensor surface. 4-fold dilution series of the indicated analytes were flowed over the captured mAbs for 120 s, followed by buffer injections for 600 s. After each cycle, surfaces were regenerated by four injections of 3 M magnesium chloride with 180 s contact times. Data were analyzed using the ProteOn Manager software (Bio-Rad). Following interspot and column double referencing, data were fitted to a Langmuir 1:1 binding model or equilibrium binding model as appropriate.

ELISA assays

Half-area 96-well ELISA plates were coated overnight at 4C with 50 μL PBS containing 250 ng of compound per well. The D7324 polyclonal sheep Ab (Aalto Bioreagents) targeting the C5 domain of gp120 was also used to capture autologous gp120 from pseudovirus stocks lysed by adding 1% NP40 for 30min at room temperature. The wells were washed four times with PBS containing 0.05% Tween 20 and blocked with 3% BSA at room temperature for 1h. Serial dilutions of sera were then added to the wells, and the plates were incubated at room temperature for 1h. After washing four times, goat anti-human IgG F(ab')2 conjugated to alkaline phosphatase (Pierce), diluted 1:1000 in PBS containing 1% BSA and 0.025% Tween 20, was added to the wells. The plates were incubated at room temperature for 1h, washed four times, and the plates were developed by adding alkaline phosphatase substrate (Sigma) diluted in alkaline phosphatase staining buffer (pH 9.8), according to the manufacturer's instructions. The optical density at 405 nm was read on a microplate reader (Molecular Devices). EC_{50} values were calculated using Prism6 (GraphPad).

QUANTIFICATION AND STATISTICAL ANALYSIS

For all mAb/serum pseudovirus neutralization and ELISA assays (Figures 1C, 4A-B, 5A, S1A, S4B-C, S5, S6, Tables S1-S4) the IC_{50}, or concentration of mAb / dilution of serum needed to obtain 50% neutralization against a given pseudovirus, was calculated from the non-linear regression of the neutralization curve. For neutralization assays in which a fold-change in IC_{50} imparted by a particular virus mutant or virus treatment was reported (Figures 5A and S6), the IC_{50} obtained for one virus/assay condition was divided by the IC_{50} obtained for the other virus/assay condition, as indicated in the figure legends. All neutralization and ELISA assays were repeated at least twice, and data shown are from representative experiments.

SPR measurements (Figure 3B) were taken over two independent experiments, and data shown are from representative experiments.

DATA AND CODE AVAILABILITY

The accession numbers for the PCIN63-UCA and PCIN63-66A to PCIN63-77F heavy chain sequences reported in this paper are GenBank: MK_749197, MK_749198, MK_749199, MK_749200, MK_749201, MK_749202, MK_749203, MK_749204, MK_749205, MK_749206, MK_749207, MK_749208, MK_749209, MK_749210, MK_749211, MK_749212, MK_749213, MK_749214, MK_749215, MK_749216, MK_749217, MK_749218, MK_749219.

The accession numbers for the PCIN63-UCA and PCIN63-66A to PCIN63-77F light chain sequences reported in this paper are GenBank: MK_749220, MK_749221, MK_749222, MK_749223, MK_749224, MK_749225, MK_749226, MK_749227, MK_749228, MK_749229, MK_749230, MK_749231, MK_749232, MK_749233, MK_749234, MK_749235, MK_749236, MK_749237, MK_749238, MK_749239, MK_749240, MK_749241.

The MiSeq PCIN63 Ab lineage heavy and light chain NGS dataset are publicly available online through BioProject: PRJNA545346.

The accession numbers for the PC63 Env clones and HQCS reported in this paper are GenBank: MK_749242, MK_749243, MK_749244, MK_749245, MK_749246, MK_749247, MK_749248, MK_749249, MK_749250, MK_749251, MK_749252, MK_749253, MK_749254, MK_749255, MK_749256, MK_749257, MK_749258, MK_749259, MK_749260, MK_749261, MK_749262, MK_749263, MK_749264, MK_749265, MK_749266, MK_749267, MK_749268, MK_749269, MK_749270, MK_749271, MK_749272, MK_749273, MK_749274, MK_749275, MK_749276, MK_749277, MK_749278, MK_749279, MK_749280, MK_749281, MK_749282, MK_749283, MK_749284, MK_749285, MK_749286, MK_749287, MK_749288, MK_749289, MK_749290, MK_749291, MK_749292, MK_749293, MK_749294, MK_749295, MK_749296.

Immunity, Volume *51*

Supplemental Information

Rapid and Focused Maturation of a VRC01-Class HIV Broadly Neutralizing Antibody Lineage Involves Both Binding and Accommodation of the N276-Glycan

Jeffrey Umotoy, Bernard S. Bagaya, Collin Joyce, Torben Schiffner, Sergey Menis, Karen L. Saye-Francisco, Trevor Biddle, Sanjay Mohan, Thomas Vollbrecht, Oleksander Kalyuzhniy, Sharon Madzorera, Dale Kitchin, Bronwen Lambson, Molati Nonyane, William Kilembe, The IAVI Protocol C Investigators, The IAVI African HIV Research Network, Pascal Poignard, William R. Schief, Dennis R. Burton, Ben Murrell, Penny L. Moore, Bryan Briney, Devin Sok, and Elise Landais

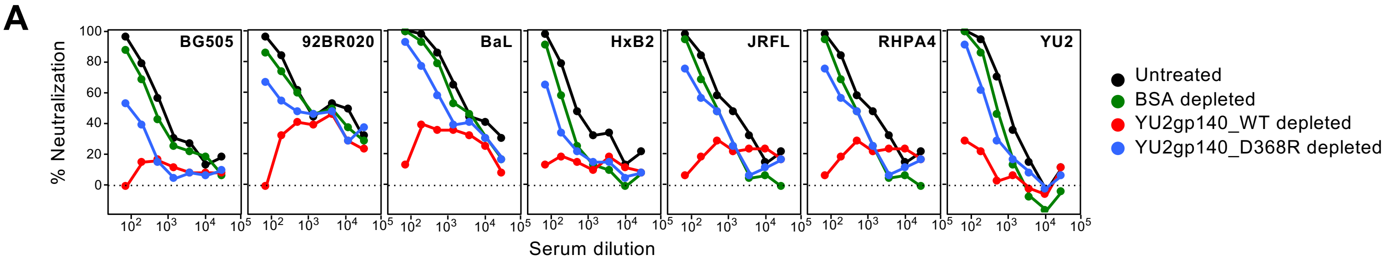

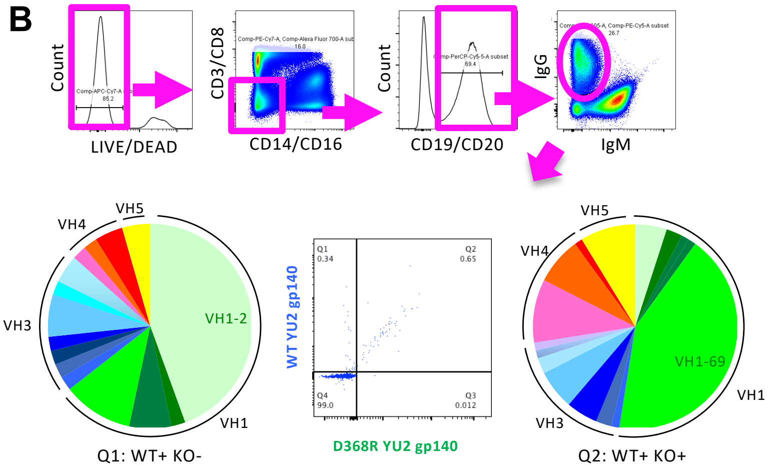

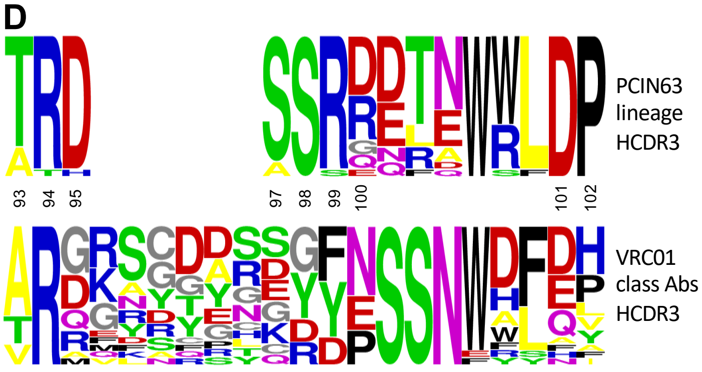

C

mpi	mAb name	CDRH3 sequence (15aa)	V_H+J_H % SHM (nt)	V_H+J_H % SHM (aa)	UCA-H % SHM (nt)	UCA-H % SHM (aa)	CDRL3 sequence (5aa)	Vκ+Jκ % SHM (nt)	Vκ+Jκ % SHM (aa)	UCA-K % SHM (nt)	UCA-K % SHM (aa)
		**Heavy Chain**					**Light Chain Chain**				
		**Putative Germline Genes: IGHV1-2*02 / IGHJ5*02**					**IGKV1-5*03 / IGKJ1*01**				
66	66Aa						QVVQW	12.04	15.63	14.56	17.76
66	66B	TRDSSRRDTNWWLDP	9.59	18.42	11.38	20.33	QVLET	12.71	17.71	14.24	18.69
71	71Aa						QVYES	10.70	19.79	12.30	20.56
71	71B	TTHSSSRDFQWSLDP	9.88	16.67	11.92	20.33	QVYES	11.04	16.67	12.94	17.76
71	71D2b	TRDSSRRDTNWWLDP	10.47	21.05	12.20	22.76	QVLET	12.71	19.79	14.24	20.56
71	71C	TRDSSRRDTNWWLDP	10.76	20.18	12.47	21.95	QVLET	12.37	18.75	13.92	19.63
71	71E1	TRDSSRRDTNWWLDP	10.76	20.18	12.47	21.95					
71	71F	TRDSSRQQRDWWLDP	12.50	21.05	13.01	21.14	QVYES	11.04	18.75	12.62	19.63
71	71G	TRDASRDDRAWRLDP	13.08	21.05	15.72	23.58	QVVEW	12.37	15.63	14.56	16.82
71	71H	ARDSSREETNWWLDP	13.08	21.93	14.36	23.58	QVLET	13.04	20.83	14.24	21.50
71	71I1a	TRDASRDDRAWRLDP	13.66	21.05	16.26	23.58	QVFEW	13.04	17.71	14.56	18.69
71	71J2a	TRDASRDDRAWRLDP	10.76	20.18	12.47	21.95	QVLET	12.71	18.75	14.24	19.63
71	71K	TRDSSRDETNWWLDP	14.24	23.68	15.45	25.20	QVLET	13.04	18.75	14.56	19.63
71	71L	TRDSSRDETNWWLDP	14.24	24.56	15.45	26.02	QVLET	13.04	18.75	14.56	19.63
71	71M1a	ARDSSRDETNWWLDP	14.53	21.05	15.72	22.76	QVLET	12.37	18.75	13.92	19.63
71	71N1a	TRDSSRGNTEWRLDP	14.53	21.05	15.72	22.76	QVYQT	12.71	18.75	14.89	20.56
71	71O	TRDSSRDNLEWRLDP	15.99	23.68	17.07	23.58	QVFEW	12.71	15.63	14.56	16.82
71	71P	TRDSSRGDTEWRLDP	15.99	23.68	17.89	24.39	QVFEW	13.04	16.67	14.89	17.76
77	77A	TTHSSRRDFQWSLDP	10.47	17.54	12.20	20.33					
77	77B1b	TRDSSRDETNWWLDP	11.34	23.68	13.82	25.20	QVLET	12.71	19.79	14.24	20.56
77	77C	TRDSSRRDLEWRLDP	11.92	21.05	12.74	21.14	QVLET	13.71	19.79	15.53	20.56
77	77D	ARDSSRDETNWWLDP	13.08	20.18	14.36	21.95	QAYES	12.37	19.79	14.24	20.56
77	77E1	TRDSSRDETNWWLDP	13.95	23.68	15.18	25.20					
77	77F1	TRDSSRDETNWWLDP	14.53	23.68	15.72	25.20					

Figure S1: Isolation of CD4bs-specific antibodies in participant PC063 – Related to Figure 1.
(A) Neutralization of HIV-1 pseudoviruses by titrated amount of PC063 month-66 (M66) untreated plasma or after adsorption on BSA- or rgp140-coated beads. This data is from a single experiment.
(B) Fluorescence Activated Cell Sorting (FACS) cell plots highlighting cell selection strategy for CD4bs specific B-cell isolation from PC063. Pie charts detail the distribution of VH-gene IMGT assignment of IgG amplicons generated from rgp140 WT+D368R- and rgp140 WT+D368R+ single sorted cells in (Quadrant #1, Q1, left) and (Quadrant #2, Q2, right).
(C) Statistics for the PCIN63 heavy chain and light chain sequences obtained from the VH1-2+ single B-cell isolated in (B). The percent of somatic hyper mutations at the nucleotide (nt) and amino-acid (aa) levels compared to the IMGT database germline gene sequences and NGS-identified unmutated common ancestor (UCA) (see Figure 3A), were calculated for V and J heavy chain (VH+JH) and light chain (VK+JK) segments. The CDR3 definitions are based on IMGT database guidelines.
(D) Logogram of the HCDR3 amino acid sequences for PCIN63 and VRC01-class Abs.

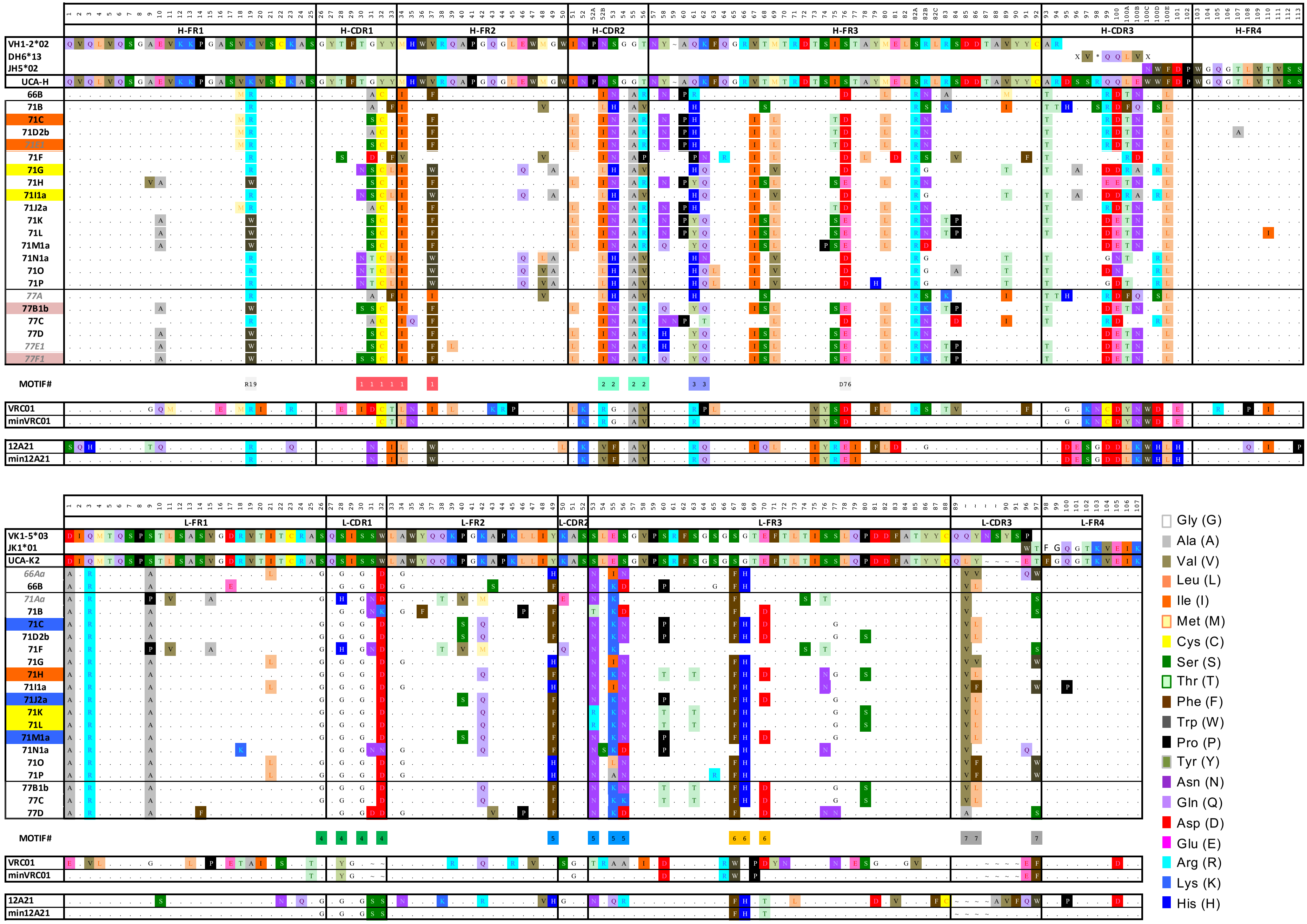

Figure S2. PCIN63 mAbs heavy chain and light chain amino acid alignments with putative germline genes – Related to Figure 1.
PCIN63 mAbs HC (top) and LC (bottom) amino-acid sequences are aligned to the IMGT database V_H1-2*02, D_H6-13, J_H5*02, Vκ1-5*03 and Jκ1*01 sequences. Identical residues are marked as a dot and somatically mutated residues are specified using one-letter coding. Deletions are marked by a ~ symbol. CDR and FRW regions are indicated. Sequences from different mAbs sharing perfect amino-acid identity are highlighted in colors. Unpaired sequences are showed in grey and italic

Clade	Time point / Ab name	PCIN 66B	PCIN 71B	PCIN 71C	PCIN 71D2b	PCIN 71F	PCIN 71G	PCIN 71H	PCIN 71I1a	PCIN 71J2a	PCIN 71K	PCIN 71L	PCIN 71M1a	PCIN 71N1a	PCIN 71O	PCIN 71P	PCIN 77B1b	PCIN 77C	PCIN 77D	PC63 M33 Serum	VRC01 min	VRC01 MAT	12A21 min	12A21 MAT
	SHM (V_H+J_H)	9.59	9.88	10.76	10.76	12.50	13.08	13.08	13.66	13.37	14.24	14.24	14.53	14.53	15.99	15.99	11.34	11.92	13.08		7.22	21.31	3.44	12.37
	SHM (V_K+J_K)	12.71	11.04	12.37	12.71	11.04	12.37	13.04	13.04	12.71	13.04	13.04	12.37	12.37	12.71	13.04	13.04	13.71	12.37		6.48	29.32	8.33	21.91
Ctrl	MLV	>100	>100	>100	>100	>100	>100	>100	>100	>100	>100	>100	>100	>100	>100	>100	>100	>100	>100	6	>100	>100	>100	>100
	VSV	>100	>100	>100	>100	>100	>100	>100	>100	>100	>100	>100	>100	>100	>100	>100	>100	>100	>100		>100	>100	>100	>100
A	92RW020	0.79	>100	0.67	0.59	>100	0.86	0.26	0.39	1.1	0.20	0.096	0.20	>100	0.58	2.2	9.5	0.94	2.4	491	0.11	0.50	0.09	0.06
	94UG103	>100	>100	>100	>100	>100	4.3	10	3.1	>100	7.1	5.7	2.4	>100	7.0	18	>100	>100	>100	1	0.20	0.35	>100	0.46
	BG505	1.3	>100	5.2	>100	>100	1.4	1.1	0.49	0.76	0.72	>100	139	>100	0.75	1.5	15	1.1	29	564	0.12	0.09	0.12	0.09
	KNH1144	1.2	>100	0.98	1.5	>100	5.1	0.45	1.3	1.1	0.32	0.42	0.66	>100	1.9	23	37	4.3	48	248	1.56	0.37	0.59	0.54
	KNH1207	0.38	>100	0.23	0.47	>100	1.2	0.33	0.36	0.25	0.29	0.29	0.62	>100	0.57	3.0	68	1.3	>100	.nt.	63	0.88	0.68	0.32
AE	92TH021	0.36	>100	0.12	9.1	>100	0.60	0.11	0.13	0.23	0.12	0.33	6.8	>100	0.30	0.36	0.94	0.24	>100	456	0.28	0.24	0.09	0.06
B	89.6	>100	>100	3.0	1.8	>100	0.24	>100	0.22	2.3	14	>100	>100	>100	1.4	0.60	>100	>100	>100	1	0.78	0.63	>100	1.96
	6535	0.62	>100	0.18	0.22	>100	1.9	2.0	0.19	0.16	0.42	0.16	0.13	>100	0.74	0.54	>100	62	>100	263	0.29	0.32	>100	9.15
	92BR020	0.26	>100	0.15	0.25	>100	0.64	0.20	0.23	0.32	0.14	0.13	0.18	>100	0.59	0.58	10	0.69	>100	834	0.81	0.04	0.11	0.08
	ADA	45	>100	2.1	5.1	>100	2.3	2.6	1.5	3.2	1.3	0.54	0.88	>100	5.6	16	50	>100	>100	192	0.32	0.48	>100	0.82
	BaL	0.11	0.041	0.025	0.044	0.71	0.093	44	0.017	0.068	1.9	1.0	21	>100	0.25	0.085	0.0074	>100	2.8	1790	0.01	0.02	>100	0.12
	CAAN	2.2	>100	0.93	0.49	>100	0.58	3.2	2.4	1.6	1.9	1.2	10	>100	9.4	7.9	8.4	10	>100	1	0.25	0.42	8.86	0.46
	DH12	46	>100	2.6	4.3	>100	3.8	32	2.6	3.4	21	8.3	5.6	>100	9.7	10	38	4.1	>100	823	0.12	0.40	>100	48
	HxB2	0.75	>100	0.067	0.24	>100	0.098	0.72	0.043	0.12	0.26	0.17	0.10	>100	0.083	0.13	0.48	>100	>100	926	0.01	0.05	>100	>100
	JRCSF	4.6	>100	0.60	0.91	>100	1.5	1.3	0.43	1.6	0.56	0.70	0.92	>100	1.4	2.7	1.6	3.3	>100	182	0.12	0.14	>100	0.43
	JRFL	0.064	>100	0.022	0.055	>100	0.14	0.082	0.053	0.052	0.053	0.037	0.070	>100	0.11	0.42	0.35	0.13	2.0	2275	71.60	0.61	0.05	0.04
	MN	0.15	>100	0.12	0.088	>100	0.075	>100	0.072	0.13	>100	>100	>100	>100	0.29	0.16	0.49	>100	>100	496	0.03	0.09	>100	20
	PVO4	1.1	>100	0.28	0.32	4.48	2.6	0.35	0.47	0.30	0.23	0.19	0.68	>100	0.89	2.4	7.2	2.4	28	402	0.19	0.35	0.29	0.41
	QHO692	6.0	>100	2.3	2.1	>100	12	1.4	1.8	1.3	1.4	1.0	1.9	>100	6.0	35	16	8.0	>100	198	3.23	1.84	7.20	4.83
	REJO	0.13	>100	0.039	0.058	>100	0.13	0.050	0.034	0.065	0.15	0.094	0.086	>100	0.13	0.15	0.096	0.42	>100	273	0.04	0.09	>100	0.79
	RHPA4	0.20	>100	0.048	0.12	>100	0.15	0.030	0.050	0.074	0.029	0.022	0.052	>100	0.089	0.23	1.8	0.16	3.4	705	21	0.04	1.37	0.04
	SC422	0.50	>100	0.12	0.36	>100	0.52	0.13	0.081	0.15	0.19	0.11	0.39	>100	0.24	0.96	1.1	>100	>100	222	0.30	0.09	>100	2.70
	SF162	>100	>100	2.4	22	>100	0.39	>100	0.19	0.99	44	12	0.89	>100	0.45	0.45	6.0	>100	>100	834	0.04	0.14	>100	0.67
	SS1196	>100	>100	8.9	35	>100	1.1	>100	0.37	9.0	19	13	3.6	>100	1.3	2.7	>100	>100	>100	378	0.09	0.22	>100	>100
	TRJO	>100	>100	272	>100	>100	0.59	0.24	0.11	62	0.080	0.080	0.12	>100	0.19	0.16	>100	>100	>100	246	0.12	0.08	>100	1.25
	TRO11	0.37	>100	0.24	0.28	>100	0.47	0.080	0.18	0.18	0.049	0.035	0.068	>100	0.31	0.79	27	0.46	99	439	2.84	0.52	0.26	0.22
	WITO	0.82	>100	0.13	0.12	>100	0.24	0.092	0.086	0.085	0.051	0.048	0.062	>100	0.078	0.078	>100	0.24	>100	496	1.07	0.17	2.89	0.22
	YU2	0.13	>100	0.058	0.10	>100	0.65	0.085	0.18	0.087	0.067	0.056	0.11	>100	0.60	0.92	7.4	0.17	15	1170	0.05	0.09	0.15	0.05
C	93IN905	0.73	>100	0.17	0.36	>100	0.34	0.063	0.090	0.21	0.035	0.014	0.056	>100	0.17	0.58	40	0.33	0.59	701	0.06	0.13	2.26	0.06
	CAP210	>100	>100	>100	>100	>100	>100	>100	45	>100	>100	>100	>100	>100	>100	>100	>100	>100	>100	1	>100	>100	>100	>100
	CAP45	>100	>100	>100	>100	>100	>100	>100	0.053	>100	>100	>100	>100	>100	0.13	0.053	>100	>100	>100	1	>100	0.27	>100	0.06
	DU156	>100	>100	7.9	21	>100	0.12	0.51	0.034	6.6	0.11	0.067	1.7	>100	0.070	0.15	>100	0.014	>100	654	0.05	0.06	6.78	0.03
	DU172	4.8	>100	0.37	0.75	>100	0.75	0.25	0.29	0.60	0.27	0.16	0.21	>100	0.38	0.76	67	1.5	10	589	>100	>100	0.56	0.29
	DU422	>100	>100	>100	>100	>100	>100	>100	>100	>100	>100	>100	>100	>100	>100	>100	>100	>100	>100	1	>100	>100	>100	3.37
	IAVIC22	>100	>100	20	52	>100	0.066	2.6	2.6	40	2.4	2.0	2.6	>100	7.9	21	>100	>100	>100	177	0.28	0.54	>100	67
	ZM53	1.1	>100	0.45	0.79	>100	1.1	0.39	0.35	0.36	0.26	0.52	0.36	>100	0.69	3.6	>100	0.77	24	.nt.	0.54	0.79	0.63	0.25
	ZM109	2.2	>100	0.98	1.0	>100	1.3	0.49	0.62	1.4	0.40	0.44	1.0	>100	7.4	9.9	>100	>100	>100	.nt.	28	0.11	>100	5.05
	ZM135	3.2	>100	1.3	1.9	>100	7.5	4.5	1.5	1.3	1.3	1.6	3.1	>100	7.4	14	>100	48	>100	1	63	1.17	>100	>100
	ZM214	0.17	>100	0.12	0.12	0.12	0.013	0.084	0.14	0.16	0.080	0.012	0.015	>100	0.19	0.14	0.74	0.021	0.46	1	0.18	0.31	0.15	0.21
	ZM249	0.43	>100	0.16	0.43	>100	0.15	0.055	0.080	0.14	0.046	0.044	0.058	>100	0.11	0.58	5.9	>100	>100	330	0.08	0.09	22	0.10

Table S1. Neutralization IC50s of PCIN63 Abs on a medium cross-clade pseudovirus panel. – Related to Figure 1 and Table S2

IC50 is defined as the Ab concentration in µg/mL allowing 50% loss of infectivity.
PCIN63 Abs are organized from least to most mutated.
Data are representative of at least 2 experiments.

Breadth (viruses neutralized)	Potency mAb (GeoMean IC_{50})	Potency Plasma (GeoMean ID_{50})
0-10%	10-50 µg/mL	/
10-25%	1-10 µg/mL	50-100
25-50%	0.1-1 µg/mL	100-300
50-75%	0.01-0.1 µg/mL	300-900
75-100%	<0.01 µg/mL	>900

Ab name	Breadth (% viruses) IC_{50} <0.1µg/mL	IC_{50} <1µg/mL	IC_{50} <10µg/mL	IC_{50} <50µg/mL	GeoMean IC_{50} (µg/mL)	SHM (V_H+J_H)	SHM (V_K+J_K)
66B	3%	45%	70%	75%	0.8	9.6	12.7
71B	3%	3%	3%	3%	0.04	9.9	11.0
71C	15%	63%	85%	88%	0.50	10.8	12.4
71D2b	10%	55%	75%	83%	0.73	10.5	12.7
71F	0%	5%	8%	8%	0.72	12.5	11.0
71G	13%	58%	90%	93%	0.57	13.1	12.4
71H	23%	55%	75%	83%	0.48	13.1	13.0
71I1a	30%	75%	95%	98%	0.28	13.7	13.0
71J2a	15%	55%	85%	88%	0.56	10.8	12.7
71K	23%	63%	80%	90%	0.42	14.2	13.0
71L	30%	63%	80%	85%	0.27	14.2	13.0
71M1a	20%	58%	80%	85%	0.55	14.5	12.4
71N1a	0%	0%	0%	0%	/	14.5	12.7
71O	10%	65%	95%	95%	0.630	16.0	12.7
71P	8%	55%	78%	95%	1.1	16.0	13.0
77B1b	5%	18%	40%	58%	4.2	11.3	12.7
77C	5%	33%	53%	58%	0.92	11.9	13.7
77D	0%	5%	15%	30%	7.3	13.1	12.4
PC63M33	25%	65%	83%	88%	125		
min VRC01	25%	68%	78%	83%	0.37	6.5	7.2
VRC01	30%	88%	93%	93%	0.21	29.3	21.3
min 12A21	8%	33%	48%	50%	0.64	8.3	3.4
12A21	25%	65%	83%	88%	0.47	21.9	12.4

Table S2. Neutralization breadth and potency of PCIN63 Abs on a medium cross-clade pseudovirus panel. – Related to Figure 1 and Table S1

PCIN63 Abs are listed vertically by increasing frequency of somatic hypermutations.

		Neutralization IC_{50} (mg/mL)									
Subtype	HIV.1 Isolate	PCIN 66B	PCIN 71G	PCIN 71I	PCIN 71L	PCIN 77D	PC63 M33 serum	Min VRC01	Mat VRC01	Min 12A21	Mat 12A21
-	aMLV	>100	>100	>100	>100	>100	<20	>100	>100	>100	>100
A	0260.v5.c1	1.5	1.7	1.7	0.58	0.95	NT	0.85	0.33	0.51	0.19
	0330.v4.c3	0.45	0.29	0.19	0.077	74	735	NT	NT	NT	NT
	191084 B7.19	0.86	0.21	0.17	0.19	>100	634	0.057	0.043	7.3	0.042
	191955 A11	>100	1.2	0.036	6.3	>100	2018	>100	>100	>100	>100
	9004SS A3 4	>100	>100	>100	>100	>100	NT	>100	>100	>100	>100
	MS208.A1	0.73	0.28	0.24	0.19	>100	530	>100	0.024	>100	6.0
	Q168ENVa2	NT	NT	NT	NT	NT	NT	NT	NT	NT	NT
	Q23ENV17	0.36	0.18	0.15	0.080	0.17	736	0.074	0.042	0.12	0.026
	Q259ENVd2.17	>100	0.77	0.073	>100	>100	93	0.20	0.029	>100	0.10
	Q461ENVe2	6.8	7.7	7.1	2.2	>100	57	1.2	0.27	0.29	0.087
	Q769ENVd22	0.22	0.094	0.070	0.079	>100	1355	0.036	0.019	0.035	0.0089
	Q842ENVd12	>100	>100	>100	>100	>100	NT	>100	>100	>100	>100
AC	3301.v1.c24	0.68	0.49	0.39	0.33	>100	558	0.11	0.14	>100	0.049
	6041.v3.c23	0.063	0.066	0.057	0.022	0.097	<20	1.8	0.013	0.014	0.0069
	6540.v4.c1	>100	>100	>100	>100	>100	24	>100	>100	>100	>100
	6545.v4.c1	>100	>100	>100	>100	>100	2	>100	>100	>100	>100
ACD	0815.v3.c3	0.12	0.057	0.062	0.032	>100	1019	0.056	0.0100	0.033	0.012
	3103.v3.c10	>100	4.0	6.4	>100	>100	<20	4.1	1.3	>100	0.47
AE	620345.c01	>100	>100	>100	>100	>100	170	>100	>100	>100	>100
	BJOX009000.02.4	>100	4.2	2.9	11	>100	<20	2.9	0.64	>100	>100
	BJOX010000.06.2	>100	16	8.5	9.9	>100	57	6.5	3.9	>100	8.0
	BJOX015000.11.5	>100	1.4	0.99	0.44	>100	36	2.0	0.19	0.60	0.13
	BJOX025000.01.1	>100	0.58	0.49	26	0.16	311	7.3	2.7	0.27	0.13
	BJOX028000.10.3	0.079	0.11	0.076	0.052	>100	1813	11	0.086	0.029	0.0091
	C1080.c03	46	3.7	2.1	1.2	>100	466	>100	0.55	0.33	0.11
	C3347.c11	5.9	0.042	0.034	0.016	0.016	3162	0.039	0.050	0.0090	0.011
	C4118.c09	>100	>100	>100	>100	>100	477	>100	>100	>100	>100
	CNE5	4.0	0.29	0.18	0.35	>100	21	0.20	0.060	0.19	0.045
	CNE8	>100	0.44	0.36	0.36	>100	76	0.42	0.74	>100	0.28
	R1166.c01	4.0	0.46	0.33	0.23	2.3	337	0.62	0.39	0.17	0.097
	R2184.c04	4.0	0.17	0.13	0.24	>100	594	0.030	0.024	0.84	0.027
	R3265.c06	0.72	0.57	0.78	0.21	>100	138	0.73	0.17	0.057	0.044
AG	928.28	0.42	0.69	0.39	0.13	>100	1116	1.1	0.22	0.42	0.20
	T211.9	2.1	1.3	0.88	0.48	>100	NT	>100	0.90	>100	>100
	T235.47	>100	0.042	0.043	0.059	>100	432	>100	0.028	0.40	0.012
	T250.4	>100	>100	>100	>100	>100	25	>100	>100	>100	>100
	T251.18	9.7	7.3	8.8	1.9	>100	114	1.7	1.3	1.9	0.43
	T255.34	0.37	0.20	0.23	0.070	1.0	264	0.71	0.39	0.072	0.028
	T257.31	0.57	0.96	1.2	0.22	>100	203	0.71	0.31	0.078	0.028
	T263.8	0.35	0.14	0.17	0.070	1.6	NT	0.094	0.12	0.11	0.044
	T278.5	>100	>100	>100	>100	>100	<20	>100	>100	>100	>100
B	1006.11.C3.1601	0.25	0.18	0.13	0.049	>100	1173	2.5	0.095	0.15	0.036
	1012.11.TC21.3257	0.28	0.87	0.49	0.65	>100	146	0.035	0.070	>100	0.035
	1054.07.TC4.1499	2.2	3.3	3.1	1.9	>100	33	0.20	0.83	>100	0.50
	1056.10.TA11.1826	2.0	1.1	0.68	0.20	>100	106	0.33	0.55	>100	0.18
	62357.14.D3.4589	0.29	0.19	0.10	0.083	0.029	4317	0.43	0.39	0.44	0.11
	6240.08.TA5.4622	6.5	4.1	2.5	0.85	>100	71	1.6	0.60	1.2	0.39
	6244.13.B5.4567	0.57	0.95	0.65	0.26	1.2	340	0.16	0.12	0.21	0.072
	AC10.0.29	>100	1.9	1.0	>100	>100	79	1.6	1.1	8.3	0.63
	CAAN5342.A2	0.72	2.6	2.2	0.76	>100	38	0.45	0.55	>100	0.49
	SC05.8C11.2344	22	2.3	1.8	6.7	>100	95	0.26	0.49	>100	1.8
	TRO.11	0.47	0.17	0.14	0.065	>100	649	0.79	0.25	0.20	0.077
	WEAU.d15.410.787	1.81	0.27	0.20	0.098	>100	214	0.027	0.046	9.98	0.012
BC	CNE17	>100	3.7	2.6	5.93	>100	<20	0.18	0.38	>100	2.7
	CNE19	0.092	0.12	0.098	0.036	0.41	708	0.027	0.074	0.13	0.019
	CNE20	0.051	0.12	0.12	0.048	>100	1510	>100	1.3	>100	0.0078
	CNE21	0.25	0.55	0.50	0.19	>100	1668	0.58	0.078	2.0	0.016
	CNE30	6.1	3.2	2.6	0.99	3.6	441	0.60	0.28	0.45	0.14
	CNE52	0.87	0.60	0.41	0.11	1.2	630	0.10	0.084	0.092	0.025
	CNE53	0.10	0.11	0.069	0.057	0.061	11658	0.035	0.046	0.065	0.027
	CNE58	0.71	0.10	0.12	0.076	1.3	2315	0.49	0.087	>100	0.078
C	1394C9G1(Rev.)	0.12	0.43	0.23	0.18	>100	465	1.2	0.15	0.058	0.058
	7030102001E5(Rev.)	12	1.4	0.91	0.39	>100	701	0.23	0.37	>100	0.22
	BF1266.431a	0.68	0.56	0.056	0.13	>100	355	0.0079	0.0077	>100	>100
	Ce0393 C3	0.15	0.38	0.26	0.15	>100	299	>100	0.12	0.015	0.013
	Ce0682 E4	>100	>100	>100	>100	>100	NT	>100	>100	>100	>100
	Ce1086 B2	>100	>100	>100	>100	>100	NT	>100	>100	>100	>100
	Ce1172 H1	>100	>100	>100	3.2	>100	<20	>100	>100	>100	>100
	Ce1176 A3	4.0	1.4	1.2	0.72	68	129	1.5	0.77	0.45	0.22
	Ce2010 F5	1.1	0.54	0.52	0.11	6.8	208	0.31	0.19	0.42	0.22
	Ce2060 G9	1.6	1.0	1.3	0.89	18	203	0.34	0.14	0.25	0.11
	Ce703010054 2A2	>100	2.0	2.8	2.4	>100	<20	0.99	0.43	>100	0.16
	Ce704809221 1B3	3.5	0.52	0.79	0.33	>100	58	0.32	0.24	0.29	0.20
	Du156.12	>100	0.088	0.11	0.18	>100	311	0.029	0.036	>100	0.22
	Du172.17	0.73	0.22	0.12	0.097	0.40	401	>100	>100	0.017	0.068
	Du422.1	>100	>100	>100	>100	>100	31	>100	>100	>100	2.0
	HIV-0013095.2.11	1.7	0.52	0.56	0.46	>100	92	0.035	0.038	>100	0.048
	HIV-001428.2.42	0.45	0.062	0.058	0.077	>100	1077	0.0067	0.011	2.2	0.0087
	HIV-16055.2.3	0.65	0.24	0.20	0.26	>100	431	0.027	0.024	0.052	0.0087
	HIV-16845.2.22	>100	>100	>100	>100	>100	NT	>100	>100	>100	>100
	ZM109F.PB4	1.1	0.44	0.55	0.23	>100	<20	0.29	0.035	>100	0.21
	ZM135M.PL10a	2.2	2.0	2.3	0.62	>100	74	>100	0.57	>100	>100
	ZM197M.PB7	>100	0.53	0.66	0.55	>100	281	0.27	0.28	>100	>100
	ZM214M.PL15	1.3	1.7	2.2	0.43	1.7	199	0.48	0.36	0.31	0.085
	ZM233M.PB6	>100	4.0	2.3	>100	>100	57	>100	>100	>100	>100
	ZM246F.D5	>100	0.77	0.87	0.22	>100	<20	>100	2.2	>100	0.088
	ZM247F.F7	>100	>100	>100	>100	>100	<20	>100	0.16	>100	0.097
	ZM249M.B10	0.64	0.12	0.13	0.051	>100	301	0.044	0.056	>100	0.031
	ZM249M.PL1	0.78	0.16	0.11	0.062	>100	313	0.044	0.073	>100	0.060
	ZM53M.PB12	7.2	2.7	2.9	1.7	32	119	0.69	0.42	0.53	0.22
CD	3817.v2.c59	>100	2.6	3.4	3.1	>100	232	>100	>100	3.8	0.82
	6480.v4.c25	0.83	0.083	0.12	0.091	>100	416	0.031	0.029	0.090	0.016
	6811.v7.c18	1.5	0.86	1.1	0.72	>100	141	0.045	0.068	0.20	0.028
	6952.v1.c20	>100	0.17	0.16	0.55	>100	230	0.063	0.020	>100	>100
	89.F1 2 25	>100	>100	>100	>100	>100	NT	>100	>100	>100	>100
D	191821 E6 1	>100	>100	>100	>100	>100	NT	>100	>100	>100	>100
	231965.c01	>100	2.3	2.8	>100	>100	59	0.25	0.32	>100	>100
	3016.v5.c45	>100	0.24	0.36	4.4	>100	NT	1.7	0.068	0.23	0.048
	A07412M1.vrc12	0.23	0.29	0.18	0.18	>100	1037	0.11	0.088	0.027	0.016
G	P0402 c2 11	0.99	0.54	0.54	0.46	>100	431	0.050	0.038	0.052	0.056
	P1981 C5 3	>100	4.27	4.14	15	>100	62	0.055	0.079	14	0.19
	X1193 c1	0.78	0.28	0.17	0.28	>100	344	0.0026	0.0013	6.46	0.066
	X1254 c3	>100	1.39	1.10	>100	>100	68	0.019	0.024	>100	0.15
	X1632 S2 B10	0.70	1.25	1.14	1.03	>100	193	0.0028	0.0047	>100	0.055
	X2088 c9	>100	>100	>100	>100	>100	<20	>100	>100	>100	>100
	X2131 C1 B5	3.61	0.80	0.81	2.71	>100	411	0.0075	0.014	>100	0.26

Breadth (viruses neutralized)	Potency mAb (GeoMean IC_{50})	Potency Plasma (GeoMean ID_{50})
0-10%	10-50 μg/mL	/
10-25%	1-10 μg/mL	50-100
25-50%	0.1-1 μg/mL	100-300
50-75%	0.01-0.1 μg/mL	300-900
75-100%	<0.01 μg/mL	>900

Table S3. Neutralization IC50s of PCIN63 on a large cross-clade pseudovirus panel. – Related to Figure 1 and Table S4.

IC50 is defined as the Ab concentration in μg/mL allowing 50% loss of infectivity for the N=120 reference pseudoviurses (Seaman et al., 2010).

PCIN63 Abs are organized from least to most mutated.

Data are representative of at least 2 experiments.

Env Subtype	PCIN 66B	PCIN 71G	PCIN 71I	PCIN 71L	PCIN 77D	PC63 M33 serum	Min VRC01	Mat VRC01	Min 12A21	Mat 12A21
	GeoMean (IC50 mg/mL)									
A	0.80	0.54	0.24	0.33	2.3	476	0.18	0.058	0.34	0.100
AC	0.21	0.18	0.15	0.086	0.097	30	0.45	0.042	0.014	0.018
ACD	0.12	0.48	0.63	0.032	/	1019	0.48	0.12	0.033	0.076
AE	2.7	0.64	0.48	0.59	0.18	234	0.83	0.28	0.14	0.087
AG	0.92	0.50	0.48	0.18	1.3	205	0.61	0.27	0.24	0.057
B	1.1	0.88	0.61	0.34	0.19	192	0.34	0.29	0.84	0.15
BC	0.33	0.40	0.33	0.18	0.68	1448	0.16	0.15	0.23	0.051
C	1.2	0.57	0.49	0.31	7.6	208	0.15	0.13	0.17	0.092
CD	1.1	0.42	0.53	0.58	/	237	0.044	0.034	0.41	0.072
D	0.23	0.54	0.56	0.90	/	246	0.36	0.12	0.079	0.028
G	1.2	0.98	0.85	1.4	/	191	0.012	0.014	1.7	0.11
Overall	0.93	0.58	0.46	0.34	1.1	255	0.21	0.13	0.25	0.084

Breadth (viruses neutralized)	Potency mAb (GeoMean IC_{50})	Potency Plasma (GeoMean ID_{50})
0-10%	10-50 μg/mL	/
10-25%	1-10 μg/mL	50-100
25-50%	0.1-1 μg/mL	100-300
50-75%	0.01-0.1 μg/mL	300-900
75-100%	<0.01 μg/mL	>900

Env Subtype	PCIN 66B	PCIN 71G	PCIN 71I	PCIN 71L	PCIN 77D	PC63 M33 serum	Min VRC01	Mat VRC01	Min 12A21	Mat 12A21
	Breadth (% viruses)									
A	64%	82%	82%	73%	27%	100%	60%	70%	50%	70%
AC	50%	50%	50%	50%	25%	25%	50%	50%	25%	50%
ACD	50%	100%	100%	50%	0%	50%	100%	100%	50%	100%
AE	50%	86%	86%	86%	21%	79%	79%	86%	64%	79%
AG	67%	78%	78%	78%	22%	71%	56%	78%	67%	67%
B	92%	100%	100%	92%	17%	83%	100%	100%	58%	100%
BC	88%	100%	100%	100%	63%	88%	88%	100%	63%	100%
C	62%	79%	79%	79%	21%	77%	62%	76%	38%	72%
CD	40%	80%	80%	80%	0%	100%	60%	60%	60%	60%
D	25%	75%	75%	50%	0%	100%	75%	75%	50%	50%
G	57%	86%	86%	71%	0%	86%	86%	86%	43%	86%
Overall	63%	84%	84%	79%	21%	80%	72%	81%	51%	77%

Table S4. Neutralization breadth and potency of PCIN63 Abs on a large cross-clade pseudovirus panel. – Related to Figure 1 and Table S3.

PCIN63 Abs are listed vertically by increasing frequency of somatic hypermutations.

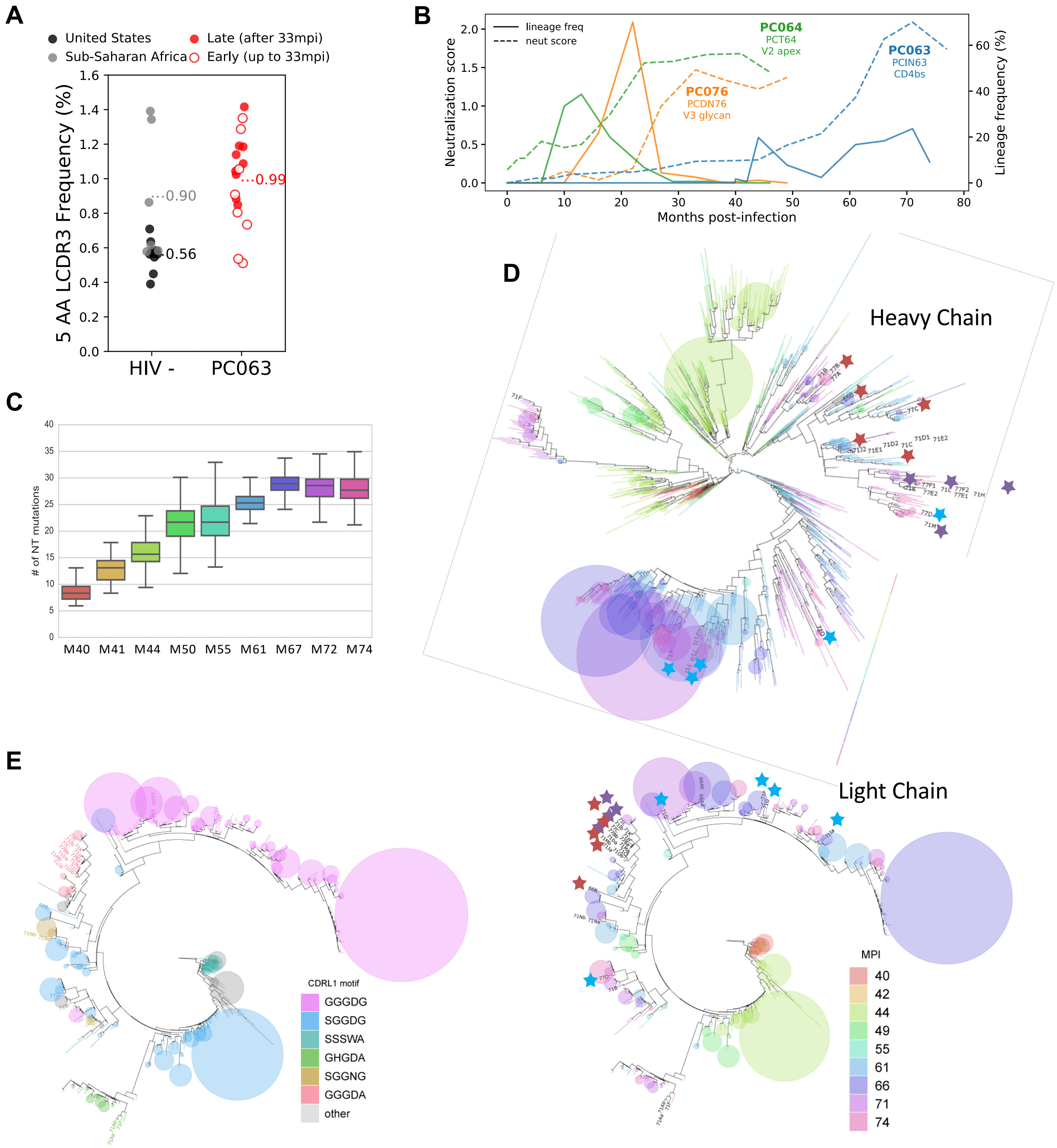

Figure S3. Emergence and development of the PCIN63 bnAb lineage – Related to Figure 2.

(A) Frequency of 5-amino acid LCDR3 immunoglobulin sequences in the naïve repertoire of PC63 (from Zambia) at different time points compared to HIV negative individuals from the United States (Southern California) and sub-Saharan Africa (Protocol C clinical research site in Zambia, Uganda, Rwanda and South Africa).

(B) Comparison of the PCIN63 Ab lineage (Blue) emergence and evolution with other bnAbs lineages isolated from two Protocol C participants (PC076: high-mannose patch targeting PCDN lineage in orange; PC064: V2-apex targeting PCT64 lineage in green) as detected in the periphery. Lineage sequences frequencies are plotted as a percentage of total lineage sequences from all time points. Plasma neutralization score (see (Landais et al., 2016)) from a heterologous medium panel is plotted as a dashed line. Related to Figure 2A.

(C) Somatic hypermutation frequency was calculated for each timepoint as divergence (number of nucleotide changes compared to LMCA). Data are presented as whisker plot showing mean, 95% upper and lower quartiles, standard deviation and outliers.

(D) Longitudinal phylogeny of PCIN63 heavy chain (Flea LINK) (Top) and light chain (Flea LINK) (Bottom) NGS sequences (colored by time-point, mpi). PCIN63 mAbs dependant (red) or accommodating (blue) the N276-glycan for neutralization or with intermediate phenotype (purple) are indicated with star symbols. Shaded circles represent the size of the cluster.

(E) PCIN63 light chain phylogeny as in (D) but colored by LCDR1-motif#4 amino acid sequence.

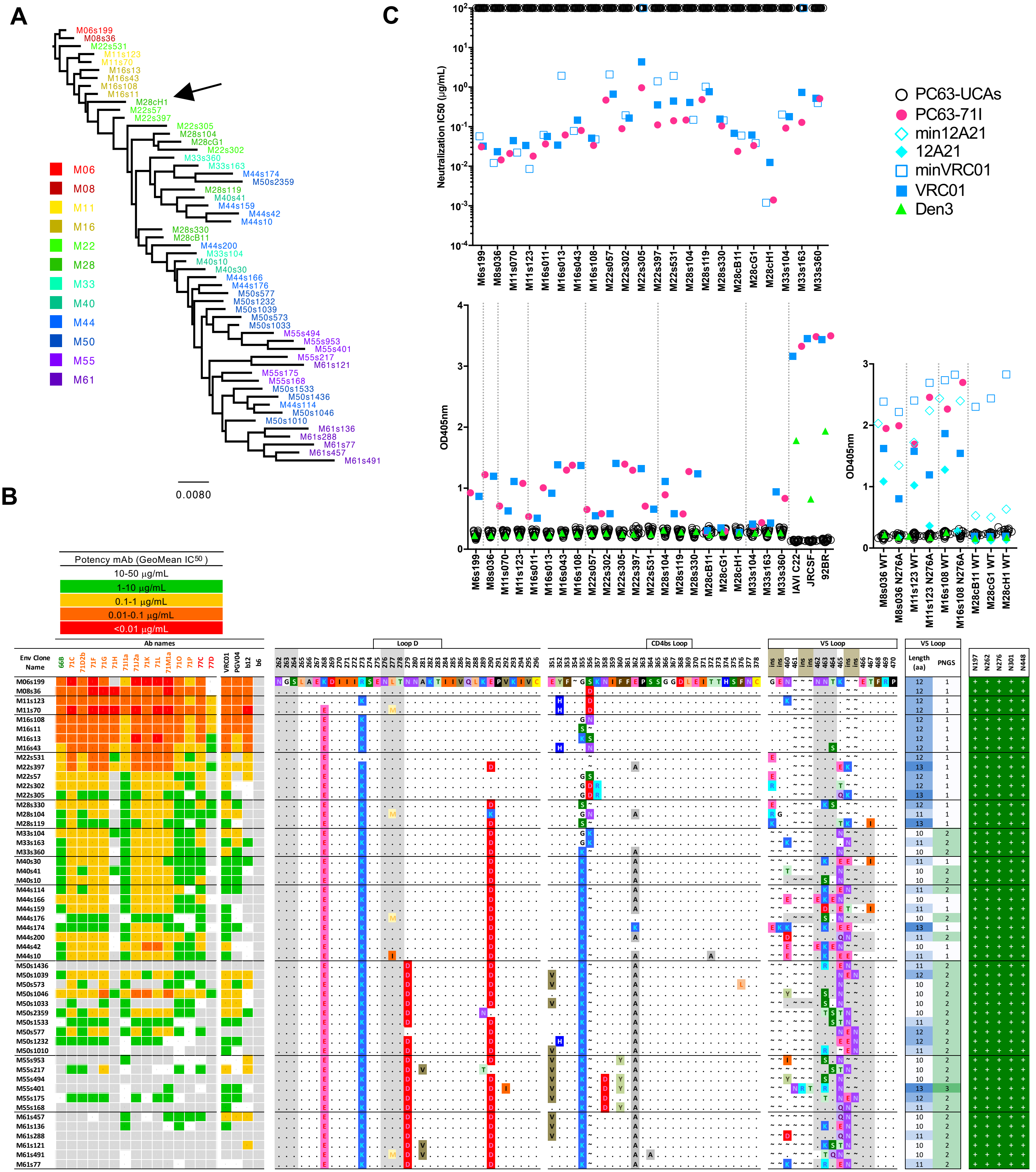

Figure S4. Elicitation of the PCIN63 lineage – Related to Figures 3 and 4.

(A) Phylogeny of PC063 HIV subtype C *env*, estimated by maximum likelihood from representative full-length *env* clonal sequences (c###) and PacBio high-quality consensus sequences (HQCS) (s###) obtained by next-generation sequencing of longitudinal plasma samples. Sequences are color-coded by time point (Months post infection, M). Clone M28cH1 is indicated with a black arrow.

(B) PCIN63 mAbs and control CD4bs Abs were tested for neutralization of pseudo-typed autologous PC063 Env clones representative of each time points (M-Months post infection, s-sequence number) as listed vertically. IC50s are tabulated and color coded as indicated in the legend. Amino-acid sequence and PNGS information of the PC063 autologous Env clones are indicated on the left. Amino-acid residues are numbered based on HxB2 alignment and color-coded as indicated in the legend. V5 loop length and number of PNGS are colored using a gradient.

(C) Putative PCIN63 UCA Abs (see Figure 3A) were tested for neutralization (Top) of representative pseudo-typed autologous Env clones (X-axis) selected from all time points prior to detection of the PCIN63 lineage in the periphery, and binding (bottom) to captured gp120 from corresponding pseudovirus stock lysates (left panel) or recombinantly expressed (right panel).

(B,C) Data are representative of at least 2 experiments.

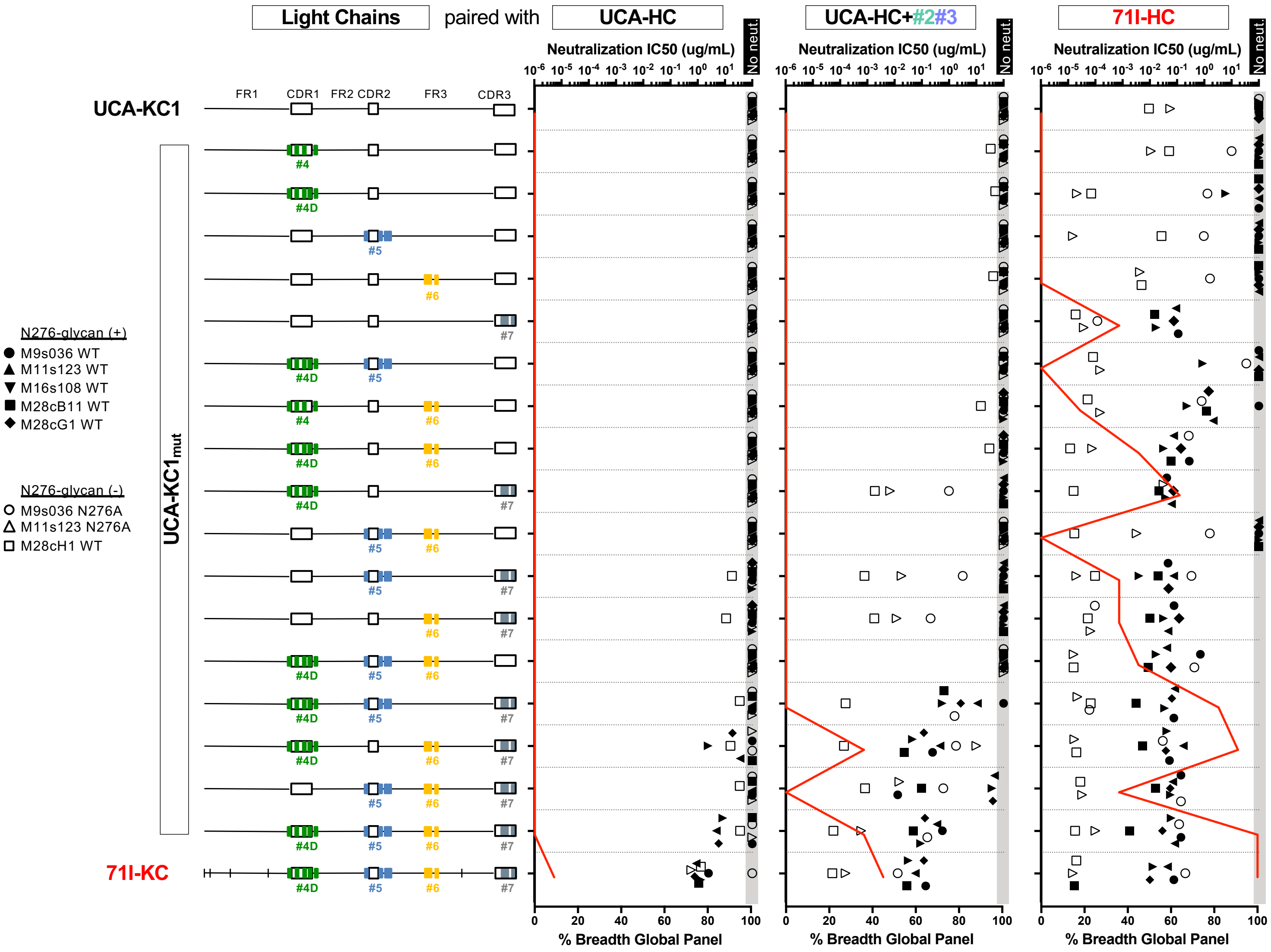

Figure S5. Functional evaluation of PCIN63 Ab lineage maturation – Related to Figure 4

PCIN63-71I-KC SHM motifs (as defined in Figure 2B) were introduced into the PCIN63-UCA-KC1, individually or in combination. Motifs are color-coded and their sequence position is indicated with a diagram. Mutated constructs were paired with WT PCIN63-UCA (left), PCIN63-UCA+Motif#2+Motif#3 (middle) or PCIN63-71I (right) HC. The chimeric Abs were tested for neutralization of the indicated WT and N276A autologous Env clones sensitive to neutralization by PCIN63-71I, as well as against the global 12-virus panel (De Camp et al., 2014). Data are representative of at least 2 experiments.

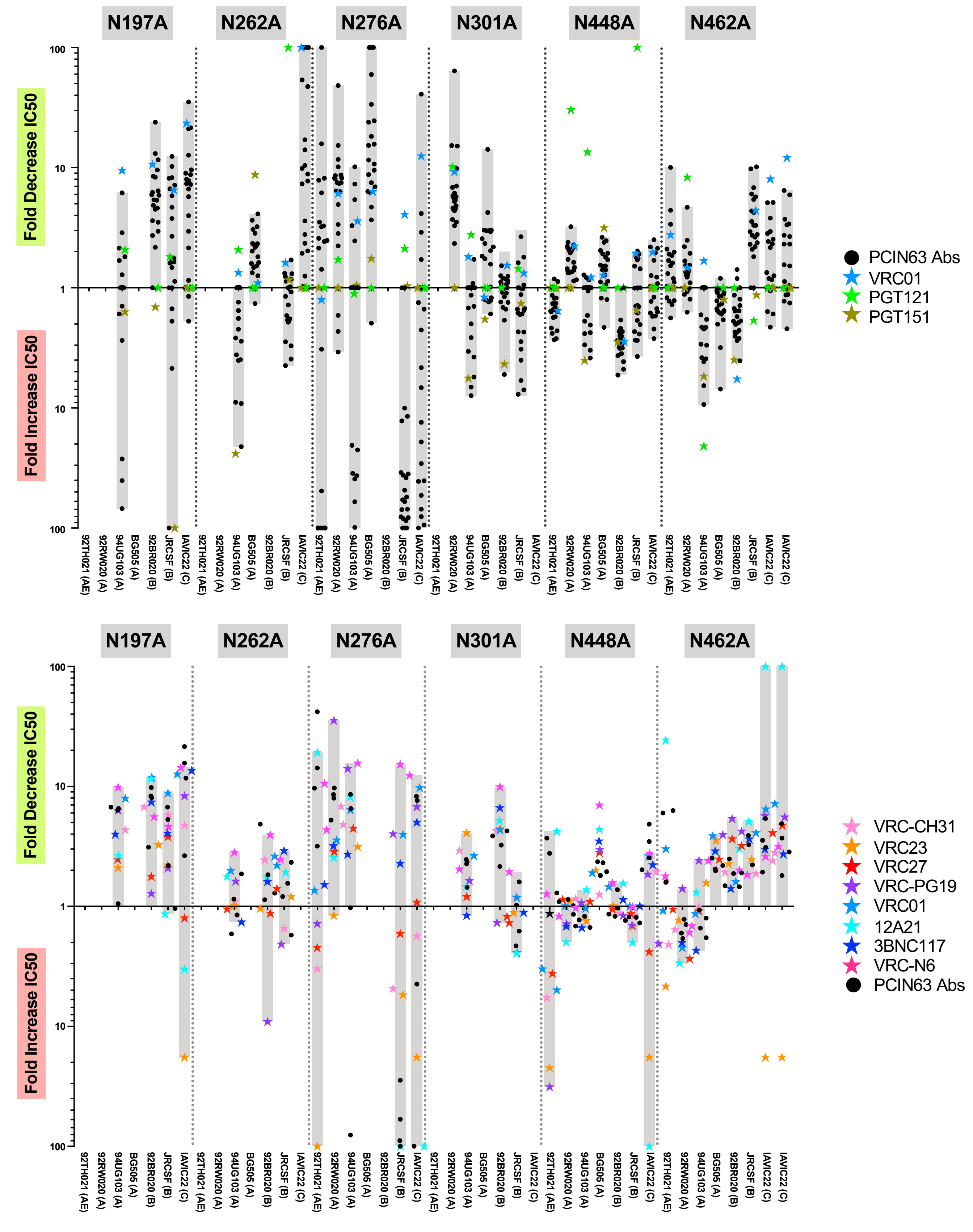

Figure S6. Effect of CD4bs N-glycan removal on neutralization by PCIN63 Abs – Related to Figure 5.
PCIN63 Abs, VRC01-class, PGT151 and PGT121 Abs were tested for neutralizing activity against the indicated pseudoviruses carrying the indicated mutation knocking down CD4bs surrounding N-glycan. The fold decrease or increase in neutralization IC_{50} is plotted. The gray bars indicate the range of variation for the PCIN63 Abs only (upper panel) or VRC01-class reference Abs (lower panel). Data are representation of at least two experiments.

**VH1-2*02**	**QVQLVQSGAEVKKPGASVKVSCKASGYTFTG~~~~~~~~~YYMHWVRQAPGQGLEWMGWINPNSGGTNYAQ~KFQGRVTM~~TR~~~~DTSIS~~~TAYMELSRLRSDDTAVYYC**	
VRC01-HC	........GQM....E.MRI..R....E.ID.........CTLN.I.L...KRP.....LK.RG.AV...R.PL...............VYSD.....FL..RS.TV......F.	TRGKNCDYN~~~~~~~~~~~WDFEH
NIH45-46-HC	..R.S...GQM....E.MRL..R....E.LN.........CPIN.I.L...RRP.....LK.RG.AV...R................VYSD.....FL..RS.T.......F.	TRGKYCTARDYYN~~~~~~~WDFEH
VRC02-HC	........GQM....E.MRI..Q....E.ID.........CTLN...L...RRP.....LK.RG.AV...R.PL...............VYSD.....FL..RS.TA........	TRGKNCDYN~~~~~~~~~~~WDFEH
VRC03-HC	.........VI.T..S...I..R....N.RD..........SI....LI.DK.F..I...K.LW.AVS..R.QL....S.....QLSQ.PDDPDWGV....F.G.TPA...E.F.	VRRGSCDYCGDFP~~~~~~~W~~QY
VRC06-HC	E....E..PVMR...S.M.I..AT...N.RD.........FSI....FNRRY.F..I...K.MW.AV...R.QL....S...S.LFSQ.LYYPDRG...L.F.G.T.A...D.F.	VRRGSSCPHCGD~~~~~~~~FHFEH
VRC07-HC	..R.S...GQM....D.MRI..R....E.IN.........CPIN.I.L...KRP.....MK.RG.AVS..R.QL...............MYSE.....FL..RS.T.......F.	TRGKYCTARDYYN~~~~~~~WDFEH
VRC08-HC	........TQM.E.....TI..VT...E.VE.........ILIN....V..R.......M..RG..V...R.Q...K............VYRD......LT..G.T.G...K.F.	VRGRSCCGGRRHCNGADCFNWDFQH
VRC18-HC	..R.....NQ.R......RI..E....K.ID.........HFI.....V..H....L.....RG..V..SRTS...KLS.TM.......NFEE......LD..K.NPG.....F.	ARGFAGY~~~~~~~~~~~~~EWSFI
VRC23-HC	...F..............R...E....S..D..........VLQ.I......RP......K.ER.AVS..P.Q....L.L.........LYTE.......HFKN.......I...	ARGVRRDAS~~~~~~~~~~~WWLQF
VRC27_HC	SQR.....PQ.R...S..RI..ET.....NA..........IL..F.....RSF......K.KF.AV...H.S....I.L.........IYRE.....FLD.TG..F........	ARDRLYDGSS~~~~~~~~~~WRLDP
VRC-CH31-HC	.........A.R......T....FAEDDDYSPYWVNPAPEHFI.FL......Q...LA.M..TN.AV...W.YLN....A.........R.MT.....FL.VKS...........	ARAQKRGRSE~~~~~~~~~~WAYAH
VRC-PG04-HC	........SG........R...WT.EDI.ER........TELI.............I..VKTVT.AV.FGSPD.RQ..SL.........RDLF.....H.DIRG.TQG...T.F.	ARQKFYTGGQG~~~~~~~~~WYFDL
VRC-PG19-HC	E.R...............R...A.......D.........FDI..L.....R.......VR.LG..VS..R.Q................FY.D.....F.DFRN.KM....L.F.	ARMGAARE~~~~~~~~~~~~WDFQY
VRC-PG20-HC	..H.M...T.M.......R.T.QT.....SD..........FI..L..V..R.F.....M..QW.QV...R.T................VYRE...V..LD.RS.TFA.....F.	ARRMRSQDRE~~~~~~~~~~WDFQH
VRC-N6-HC	RAH.....TAM.......R...QT......A.........HILF.F.....R....V...K.QY.AV.FGG.G.RD...L.........VYRE...I...DIRG.KP........	ARDRSYGDSS~~~~~~~~~~WALDA
12A12-HC	SQH.....TQ........RI..Q....S..D..........VL..W..............K.VY.AR...R.R....INF..D......IYRE...I.F.D..G.......L.F.	ARDGSGDDTS~~~~~~~~~~WHLDP
12A21-HC	SQH.....TQ........R...Q.......N..........IL..W............L.K.VF.AV...R.Q....IQL.........IYRE...I.FLD..G...........	ARDESGDDLK~~~~~~~~~~WHLHP
3BNC60-HC	..H.S....A.T......R...E....KISD.........HFI..W........Q.V.....KT.QP.NPR.Q.....SL....QASW.FDTY...SF..D.KAV......I.F.	ARQRSDF~~~~~~~~~~~~~WDFDV
3BNC117-HC	....L....A.T......R...E....NIRD..........FI..W........Q.V.....KT.QP.NPR.Q.....SL....HASW.FDTF...SF..D.KA.........F.	ARQRSDY~~~~~~~~~~~~~WDFDV
N60P25.1	H.......T...R.....RI..AS.....SN..........FI........R.......M..LR.AV..SG..................IYTE....SF.V..G.......I.FC	ARGRDGYE~~~~~~~~~~~~YGFNP
N49P7	~AD......V.....D..RI..E.Q..R.PD..........II..I.R.....P.....M..MG.QV.IPW.......S.............E.....FLD.RG.K........C	VRDRSNGSGKRFESSN~~~~WFLDL
DRVIA7	.....E..TQFRR.....R...E......IS.........SFI..I..G..........M..RH.AV...PRR...K...............D.........R........M.F.	VTSRTKDYDE~~~~~~~~~~WWFDP
PCIN63-71I	..................R..........NS.........CLI..W........Q..A....LH.AV...H.Q....I.V............D......RG.......T...T..	TRDASRDDRA~~~~~~~~~~WRLDP

**VK3-20*01**	**EIVLTQSPGTLSLSPGERATLSCRASQS~~VSSSYLAWYQQKPGQAPRLLIYGASSRATGIPDRFS~GSGSGTDFTLTISRLEPEDFAVYYC**	
VRC01-KC	.................T.II...T..Y..~~~GS......R.......V..SG.T..A..........RW.P.YN....N..SG..G....	QQYEF
NIH45-46-KC	........A........T.II...T.....~~~GS......R.......V..SG.T..A..........RW.A.YN.S..N..SG..G....	QQYEF
VRC02-KC	.................T.II...T..Y..~~~GS......R.......V..SG.T..A..........RW.P.YN...RN..SG..GL...	QQYEF
VRC03-KC	.........I.......T...F.K...G..~~GNAMT...KRR..V......DT.R..S.V....V.........F...NK.DR........	QQFEF
VRC06-KC	........A..................G..~~GNS.N...KRR..T......DT.R..SD..EK.V.........S...TKVG.........	QQFEF
VRC07b-KC	........A..........I....T..Y..~~~GS......R.......V..AG.T.............RW.AEYN....N..S...G....	QQYEF
VRC08-KC	Y.GV....AI..V.L...V....KT..A..ITPRH.V.HR..G....S.VMT.T.E..S......I.............T...A........	QCLEA
VRC18b-KC	.................T......T..G..IL.NQ...H..RR..P.......G.N..P...E..T.........V...K...RD.......	QILEF
VRC-PG04-KC	.................T.S...T.ASY..~~~GHMT...K....P.K...FAT.K..S..........QF.KQY....T.M......R...	QQLEF
3BNC60-KC	D.QM....SS..ARV.DTV.IT.Q.NGY..~~~~~.N....RR.K..K....DG.KLER.V.A.....RRW.QEYN...NN.Q...V.T.F.	QVYEF
3BNC117-KC	D.QM....SS..A.V.DTV.IT.Q.NGY..~~~~~.N....RR.K..K....DG.KLER.V.S.....RRW.QEYN...NN.Q...I.T.F.	QVYEF
**VK3-15*01**	**EIVMTQSPATLSVSPGERATLSCRASQS~~~VSSNLAWYQQKPGQAPRLLIYGASTRATGIPARFS~GSGSGTEFTLTISSLQSEDFAVYYC**	
VRC23-KC	........V.V...R.GT.........G....G.DV....H....T............S.V.E.......FHVD...S..G..P..V.I...	QQYET
**VK1-33*01**	**DIQMTQSPSSLSASVGDRVTITCQASQD~~~ISNYLNWYQQKPGKAPKLLIYDASNLETGVPSRFS~GSGSGTDFTFTISSLQPEDIATYYC**	
VRC27-KC	...........AV...G......R...G....GSD.H.....R.RP..I..HH..AR.E......G.....H.P.I...ND..LD.V.....	QVIES
VRC-CH31-KC	..............L...........RG....GKD.......A.......VS...T..G...........FHQN.SL......A..V...F.	QQYET
VRC-N6-KC	Y.HV........V.I......N..T..G...VGSD.H...H...R......HHT.SV.D...........FH.S.NL...D..AD.......	QVLQF
12A12-KC	.........................G.G....GSS.Q.............VHG....HR...........FH.T.SL...G..RD.F...F.	AVLEF
12A21-KC	.....................N...G.G....GSS.....K...R.....VHG....QR...........FH.T..L.......D.V...F.	AVFQW
**VL2-14*01**	**QSALTQ PASVSGSPGQSITISCTGTSSDVGGYNYVSWYQQHPGKAPKLMIYEVSNRPSGVSNRFS~GSKSGNTASLTISGLQAEDEADYYC**	
VRC-PG19-KC	........................~A...FR.FSS.......V..R....L.FS.NR.....I.H....................I......H.	NAYEF
VRC-PG20-KC	........P...........L....A.~~~~~~TS.A....YAD...R.IVFDGNK...DI.S......Q..G..........S....Y.H.	NAFEF
**VL2-11*01**	**QSALTQ PRSVSGSPGQSVTISCTGTSSDVGGYNYVSWYQQHPGKAPKLMIYDVSKRPSGVPDRFS~GSKSGNTASLTISGLQAEDEADYYC**	
N49P7	............A.............HN~~~~~~L...C.HQ..R....L...FN..............G..G......T...DD.D.E.F.	WAYEA
**VK1-5*01**	**DIQMTQSPSTLSASVGDRVTITCRASQS~~~ISSWLAWYQQKPGKAPKLLIYKASSLESGVPSRFS~GSGSGTEFTLTISSLQPDDFATYYC**	
DRVIA7	........V.....I............R....DN.V........R...........I.T..................S.N......V.....	QQFEE
N60P25.1	YV~........Y............S......~~G.K.................S.T.............D.....N...T..........F.	QHFET
PCIN63-71I	........A...........L....G.G....G.D.G..............H..N.IN............FH.......N............	QVFEW

Table S5. VRC01-class Abs aminoacid sequence aligment with their putative germline V-gene– Related to Figure 5 and S6

CDRH3 Sequences are highlighted in grey. Motifs (as defined in Figure 2) are highlighted in the corresponding colors.
